# Supplementary material for: Effect and safety of ethanolamine oleate in sclerotherapy in patients with difficult-to-resect venous malformations: A multicenter, single-arm study
Source: PLoS One. 2025 Jan 31;20(1):e0303130. doi: 10.1371/journal.pone.0303130 (PMC11785324; doi:10.1371/journal.pone.0303130)
Supplement: S2 Protocol — (DOCX) [file pone.0303130.s002.docx]

Appendix 1

1. 　Organization

1.1　Investigators

| Institution | Investigator | Tel |
| --- | --- | --- |
| Department of Plastic, Reconstructive and Aesthetic Surgery, Kyorin University Hospital  6-20-2 Shinkawa, Mitaka, Tokyo 181-8611, Japan | Mine Ozaki | +81-422-47-5511 |
| Department of Plastic and Reconstructive Surgery, The University of Tokyo Hospital  7-3-1 Hongo, Bunkyo-ku, Tokyo 113-8655, Japan | Masakazu Kurita | +81-3-3815-5411 |
| Department of Plastic and Reconstructive Surgery, Juntendo University Urayasu Hospital  2-1-1 Tomioka, Urayasu, Chiba 279-0021, Japan | Takeshi Nojiri | +81-47-353-3111 |
| Department of Plastic and Reconstructive Surgery, Keio University Hospital  35 Shinanomachi, Shinjuku-ku, Tokyo 160-8582, Japan | Noriko Aramaki | +81-3-3353-1211 |
| Department of Plastic and Reconstructive Surgery, Division of Sensory Organs and Morphological Surgery, National Center for Child Health and Development  2-10-1, Okura, Setagaya-ku, Tokyo 157-8535, Japan | Makoto Hikosaka | +81-3-3416-0181 |
| Department of Plastic and Reconstructive Surgery, Shinshu University Hospital  3-1-1 Asahi, Matsumoto, Nagano 390-8621, Japan | Shunsuke Yuzuriha | +81-263-35-4600 |
| Department of Diagnostic Radiology, Osaka Medical and Pharmaceutical University Hospital  2-7 Daigaku-cho, Takatsuki, Osaka 569-8686, Japan | Keigo Osuga | +81-72-683-1221 |
| Department of Plastic and Reconstructive Surgery, Kobe University Hospital  7-5-2 Kusunoki-cho, Chuo-ku, Kobe, Hyogo 650-0017, Japan | Tadashi Nomura | +81-78-382-6251 |

1.2　Coordinating Committee (Coordinating Physician)

Tadashi Nomura (Chair)

Department of Plastic and Reconstructive Surgery, Kobe University Hospital

7-5-2 Kusunoki-cho, Chuo-ku, Kobe, Hyogo 650-0017, Japan

TEL：+81-78-382-6251

Mine Ozaki

Department of Plastic, Reconstructive and Aesthetic Surgery, Kyorin University Hospital

6-20-2 Shinkawa, Mitaka, Tokyo 181-8611, Japan

TEL：+81-422-47-5511

［Scope of Work.]

Coordination of the details of the protocol among multiple sites, review and finalization of the protocol, contracting with the Coordinating Office and contract research organizations, etc., notification of the clinical trial plan, safety reporting to the Minister of Health, Labour and Welfare, coordination of questions arising during the clinical trial regarding the interpretation of the protocol, coordination among multiple sites regarding responses to situations arising during the clinical trial, advice on overall clinical trial operations, and advice on the consideration of problem cases, etc. Coordination of multicenter regarding responses to situations that arise during a clinical trial, advice on overall clinical trial operations, and advice on the review of problem cases, etc.

1.3　Coordinating Office

Akihide Konishi, Yasumasa Kakei, Tomoyuki Kodama, Naoko Kashiwagi

Clinical & Translational Research Center, Kobe University Hospital

7-5-2 Kusunoki-cho, Chuo-ku, Kobe, Hyogo 650-0017, Japan

TEL：+81-78-382-6729

FAX：+81-78-382-5827

Email：ctrcpj-eo1@med.kobe-u.ac.jp

［Scope of Work.]

Support for various clinical trial coordination tasks, including project management, to ensure that the tasks performed by the Coordinating Committee are carried out smoothly.

1.4　Efficacy and Safety Evaluation Committee

Mitsunaga Narushima (Chair)

Department of Plastic and Reconstructive Surgery, Graduate School of Medicine, Mie University

2-174 Edobashi, Tsu, Mie 514-8507, Japan

TEL：+81-59-232-1111

Akira Kitagawa

Department of Radiology, Aichi Medical University Hospital

1-1 Iwasaku-Ganmata, Nagakute, Aichi 480-1195, Japan

TEL: +81-561-62-3311

Eisuke Inoue

Research Promotion Center, Showa University

1-5-8 Hatanodai, Shinagawa-ku, Tokyo 142-8555, Japan

TEL: +81-3-3784-8863

［Scope of Work.]

To ensure the safety of subjects and to guarantee the integrity of the clinical trial as much as possible, the committee will discuss the continuation or discontinuation of the clinical trial and any major changes to the protocol, and provide appropriate advice and recommendations to the Coordinating Committee.

1.5　Efficacy and Safety Evaluation Office

Akihide Konishi, Yasumasa Kakehi, Tomoyuki Kodama, Naoko Kashiwagi

Clinical & Translational Research Center, Kobe University Hospital

7-5-2 Kusunoki-cho, Chuo-ku, Kobe, Hyogo 650-0017, Japan

TEL：+81-78-382-6729

FAX：+81-78-382-5827

Email: ctrcpj-eo1@med.kobe-u.ac.jp

［Scope of Work.]

To ensure that the work performed by the Efficacy and Safety Evaluation Committee is carried out smoothly, the office shall perform administrative tasks related to the operation of the committee, preparation and storage of materials and records, and other tasks.

1.6　Central Judging Committee

Shinpei Akiyama

Department of Diagnostic Radiology and Therapy, Kyoto Prefectural University of Medicine

465 Kaji-cho, Hirokoji-agaru, Kawaramachi-dori, Kamigyo-ku, Kyoto, 602-8566, Japan

TEL：+81-75-251-5111

Junko Ochi

Department of Diagnostic Radiology, Suita Tokushukai Hospital

21-1 Senrigaoka Nishi, Suita, Osaka 565-0814, Japan

TEL：+81-6-6878-1110

［Scope of Work.]

Based on the calculation method determined by the lesion volume calculation method review committee, the volume of venous malformations (cystic lesions and diffuse lesions) will be calculated for each case and evaluated for the primary and secondary endpoints (volume-related).

1.7　Committee on Methods of Calculating Lesion Volume

Taiki Nozaki

Department of Radiology, School of Medicine, Keio University

35 Shinanomachi, Shinjuku-ku, Tokyo 160-8582, Japan

TEL：+81-3-3353-1211

Michio Ozeki

Department of Pediatrics, Gifu University Hospital

1-1 Yanagido, Gifu, Gifu, 501-1194, Japan

TEL：+81-58-230-6000

Takeshi Hara

Department of Electrical, Electronics and Computer Engineering, Faculty of Engineering, Gifu University

1-1 Yanagido, Gifu, Gifu 501-1193, Japan

TEL：+81-58-293-2365

Shinpei Akiyama

Department of Diagnostic Radiology and Therapy, Kyoto Prefectural University of Medicine

465 Kaji-cho, Hirokoji-agaru, Kawaramachi-dori, Kamigyo-ku, Kyoto, 602-8566, Japan

TEL：+81-75-251-5111

Junko Ochi

Department of Diagnostic Radiology, Suita Tokushukai Hospital

21-1 Senrigaoka Nishi, Suita, Osaka 565-0814, Japan

TEL：+81-6-6878-1110

［Scope of Work.]

Methods of calculating the volume of each lesion of venous malformations (cystic lesions and diffuse lesions) will be discussed and presented as criteria for the Central Judging Committee.

1.8　QOL Evaluation Methodology Review Committee

Keiko Kamibeppu

Department of Maternal Nursing and Midwifery, Division of Health Sciences and Nursing, Graduate School of Medicine, The University of Tokyo

7-3-1 Hongo, Bunkyo-ku, Tokyo 113-0033, Japan

TEL：+81-3-5841-3556

Takafumi Soejima

Department of Family Nursing, Graduate School of Health Sciences, Kobe University

7-10-2 Tomogaoka, Suma-ku, Kobe, Hyogo 654-0142, Japan

TEL：+81-78-792-2555

［Scope of Work.]

Consider and determine an appropriate quality of life rating scale for each age group.

1.9　Academic Research Organization (ARO)

Akihide Konishi, Yasumasa Kakei, Tomoyuki Kodama, Naoko Kashiwagi

Clinical & Translational Research Center, Kobe University Hospital

7-5-2 Kusunoki-cho, Chuo-ku, Kobe, Hyogo 650-0017, Japan

TEL：+81-78-382-6729

［Scope of Work.]

Provide assistance in the preparation of clinical trial protocols.

1.10　Statistical Analysis Manager

Keiko Miyakoda

Clinical & Translational Research Center, Kobe University Hospital

7-5-2 Kusunoki-cho, Chuo-ku, Kobe, Hyogo 650-0017, Japan

TEL：+81-78-382-6271

［Scope of Work.]

Develop a plan for the statistical analysis work to be performed by the CRO. Review the results of statistical analyses and take responsibility for the results.

1.11　Person in charge for preparing the clinical study report

Tadashi Nomura

Department of Plastic and Reconstructive Surgery, Kobe University Hospital

7-5-2 Kusunoki-cho, Chuo-ku, Kobe, Hyogo 650-0017, Japan

TEL：+81-78-382-6251

［Scope of Work.]

Based on the results of this clinical trial, the clinical study report (CSR) shall be prepared; the contents of the CSR shall be discussed with the Coordinating Committee to obtain its opinion and, if necessary, be appropriately reflected in the CSR.

1.12　Contract Research Organization (CRO)

DOT World Corporation

2-14-1 Higashi-Shinbashi, Minato-ku, Tokyo 105-0021, Japan

TEL: +81-3-3433-6060

［Scope of Work.]

Conduct operations, coordinate between departments, manage schedules, and perform other tasks related to this clinical trial, including the following 1) to 6) below.

1) The person in charge for receiving the investigational drug:

Noriko Tomuro

Administrative Support Group, Clinical Operations Department

［Scope of Work.]

Responsible for receiving the investigational drugs from the sponsor, storing and delivering of the investigational drug. The work will be performed by an outside contractor under the supervision of the person in charge for receiving the investigational drug.

2) Data Management Officer:

Hideaki Shiraishi

DM Group, Data Science Dept.

［Scope of Work.]

Data management services.

3) Statistical analyst:

Tomonori Kimura

STAT Group, Data Science Dept.

［Scope of Work.]

Statistical analysis services.

4) Person in charge of preparing the general report:

Mutsumi Utsuno

Regulatory Science Department

［Scope of Work.]

Support for preparing CSR.

5) Monitoring Manager:

Shunichi Ohgiya

Clinical Operations Department

［Scope of Work.]

Monitoring services.

6) Audit Manager:

Yoshihisa Matsuura

Reliability Assurance Group

［Scope of Work.]

Auditing services.

1.13　Investigational drug provider

Shinya Toba

Formulation Development Department, Fuji Chemical Industry Co. Ltd.

1 Gokakizawa, Kamii-cho, Nakashinagawa-gun, Toyama 930-0405, Japan

TEL：+81-76-461-3534

［Scope of Work.]

Manufacture, provide, and deliver investigational drugs to the person in charge for receiving the investigational drugs.

Revision History

| No. | Creation/revision date | Reason for revision |
| --- | --- | --- |
| Ver. 1.01 | Jun 24, 2020 | newly enacted |
| Ver. 1.11 | Jul 17, 2020 | Change of the version number due to changes in the main body of the protocol |
| Ver. 1.21 | Sep 14, 2020 | Change of CRO person in charge, maintenance of description |
| Ver. 1.31 | Jan 6, 2021 | Specify the chairperson of the efficacy and safety evaluation committee, change of the name of the CRO organization/contact person, and improving the description |
| Ver. 1.41 | Apr 1, 2021 | Institutional name change, personnel change, position deletion, CRO organization/contact person change |
| Ver. 1.51 | Jun 18, 2021 | Change of affiliation of QOL evaluation method review committee members, and telephone number of CRO |
| Ver. 1.61 | Oct 21, 2021 | Change of CRO contact person |
| Ver. 1.62 | Feb 22, 2022 | Change of the person in charge of statistical analysis, and the name of the CRO organization/contact person |
| Ver. 1.71 | Oct 26, 2022 | Personnel changes, CRO organization name change and contact person change |
| Ver. 1.81 | Dec 7, 2022 | Change of CRO contact person |
| Ver. 1.82 | Apr 25, 2023 | Change the name of the investigator's department, the affiliation of the lesion volume calculation method review committee members, and addition of the persons in charge of the Coordinating Office, Efficacy and Safety Evaluation Office, and ARO |
