# Supplementary material for: Effect and safety of ethanolamine oleate in sclerotherapy in patients with difficult-to-resect venous malformations: A multicenter, single-arm study
Source: PLoS One. 2025 Jan 31;20(1):e0303130. doi: 10.1371/journal.pone.0303130 (PMC11785324; doi:10.1371/journal.pone.0303130)
Supplement: S3 Protocol — (DOCX) [file pone.0303130.s003.docx]

Appendix 2

Questionnaire on Quality of Life and Pain

(1) For the patient himself/herself (2-4 years old)

(2) For the patient himself/herself (5 years old)

(3) For the patient himself/herself (6-7 years old)

(4) For the patient himself/herself (8-12 years old)

(5) For the patient himself/herself (13-14 years old)

(6) For the patient himself/herself (15 years and older)

(7) For parents (0-year-old patients)

(8) For parents (1 year old patients)

(9) For parents (2-4 years old patients)

(10) For parents (5 years old patients)

(11) For parents (6-7 years old patients)

(12) For parents (8 - 14 years old patients)

1. For the patient himself/herself (2-4 years old)


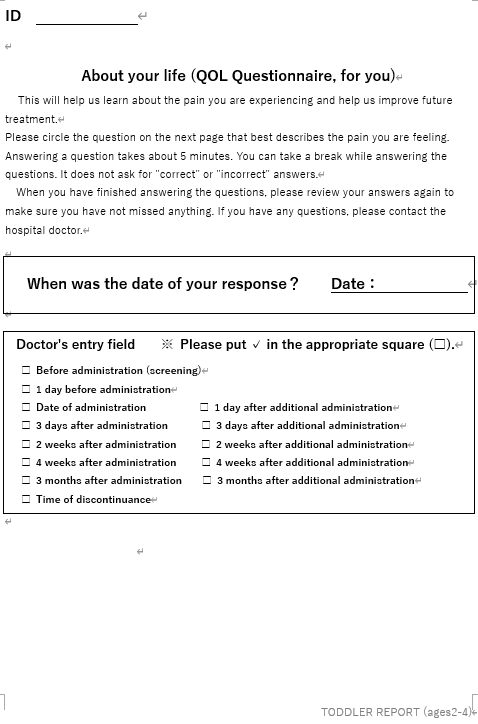


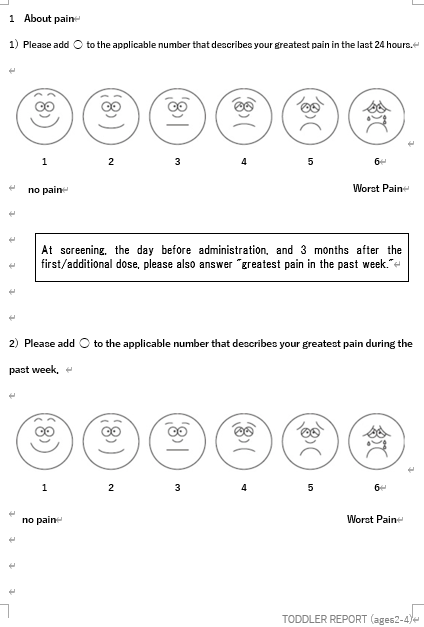


1. For the patient himself/herself (5 years old)


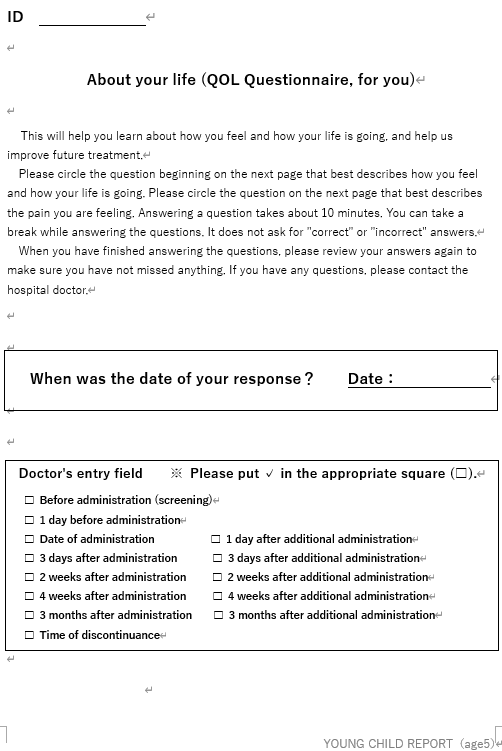


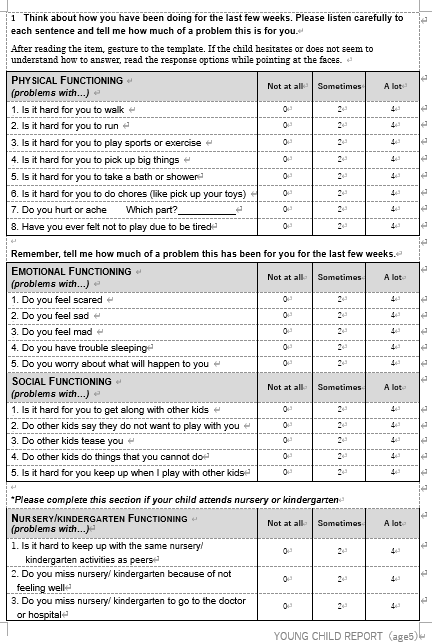


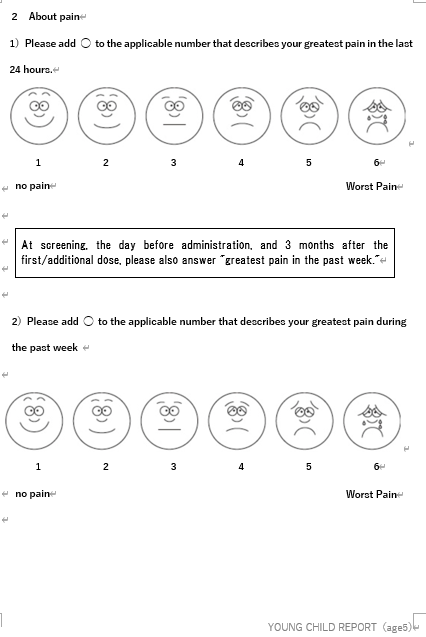


Supporting data


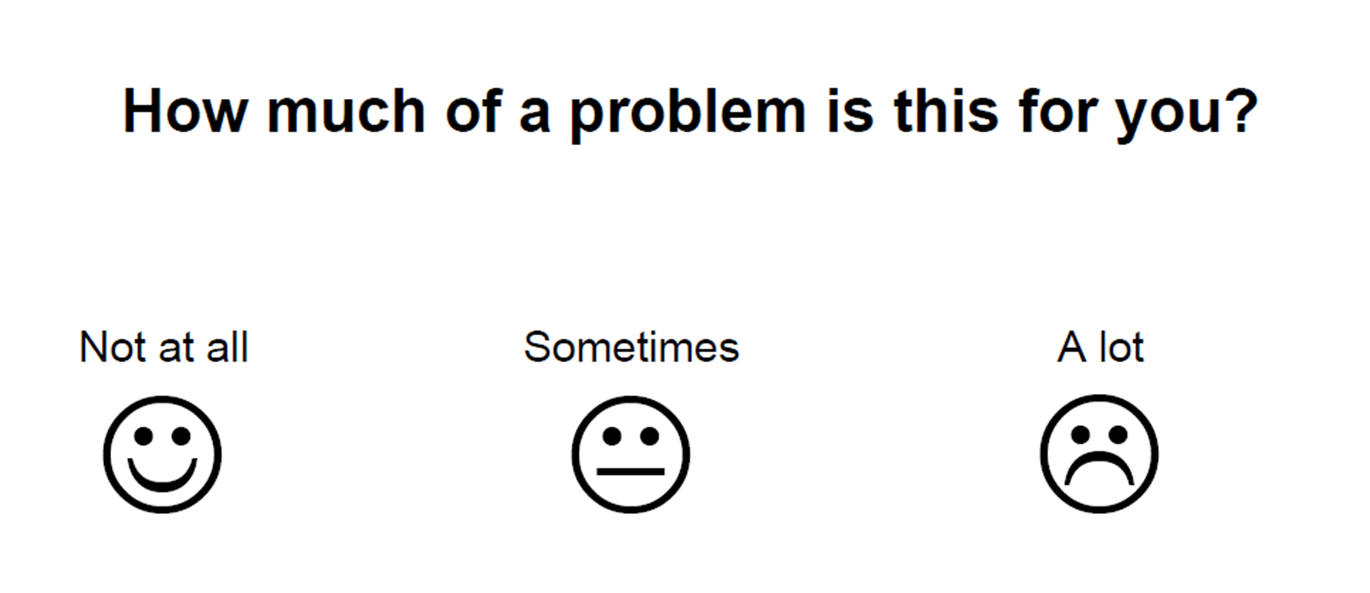


1. For the patient himself/herself (6-7 years old)


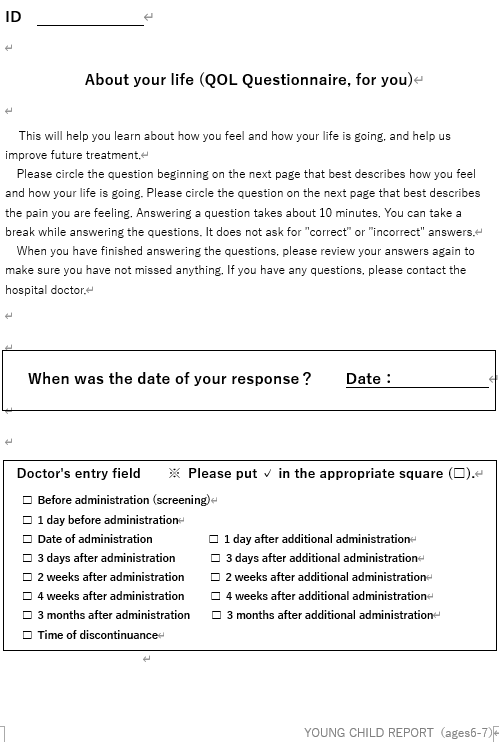


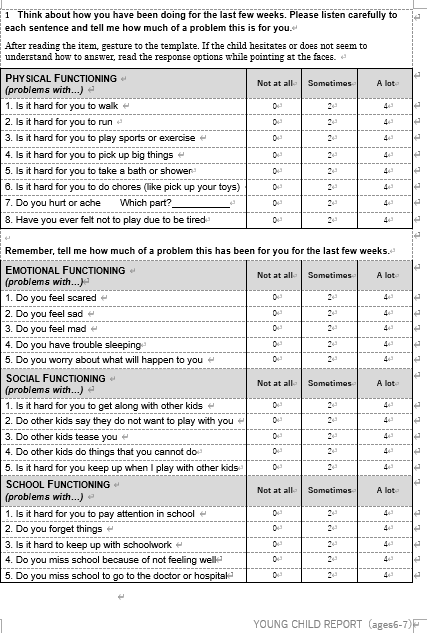


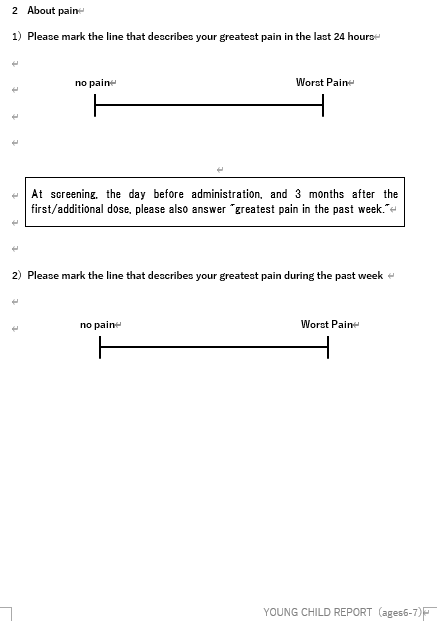


Supporting data


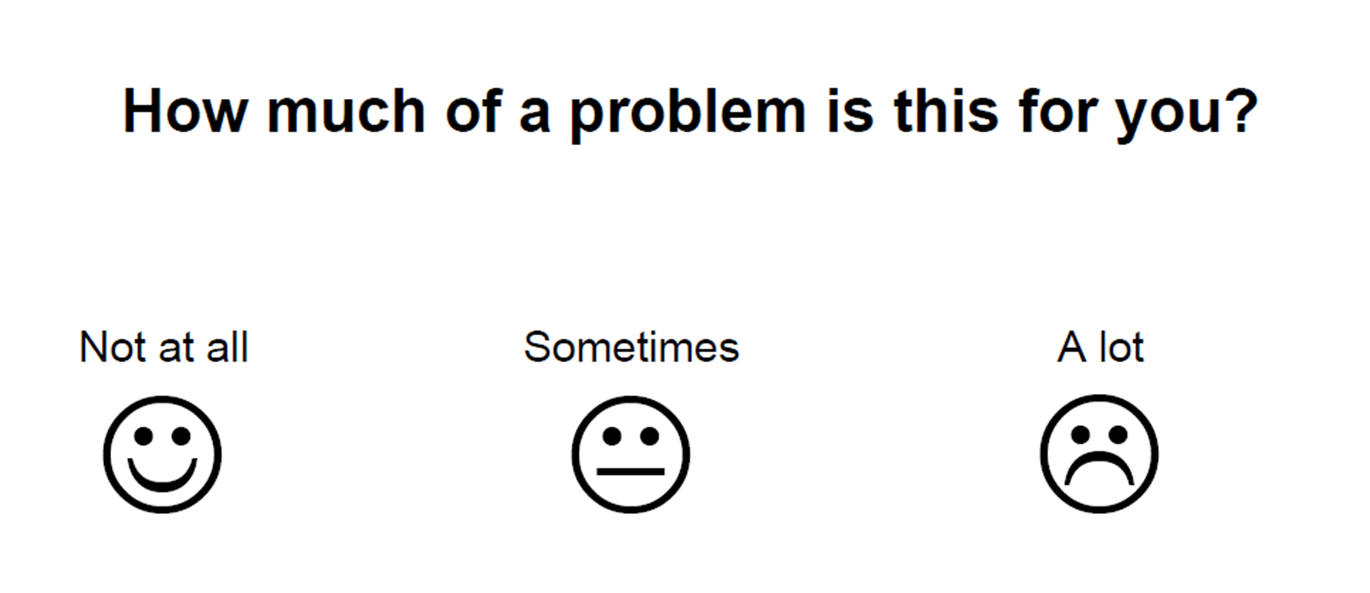


1. For the patient himself/herself (8-12 years old)

*
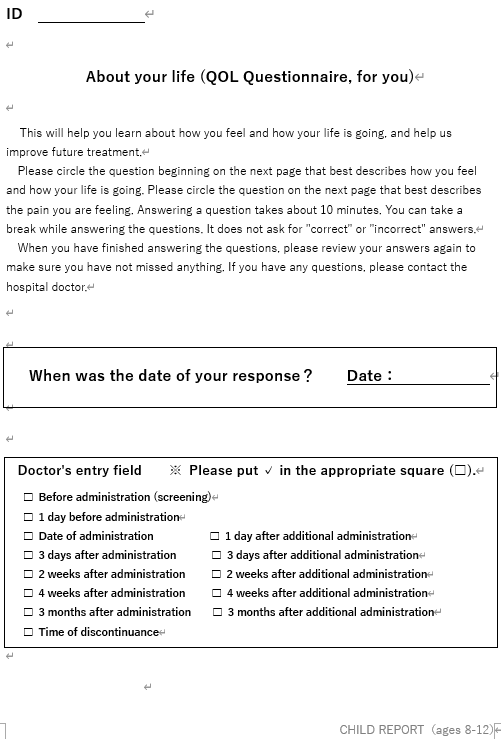
*


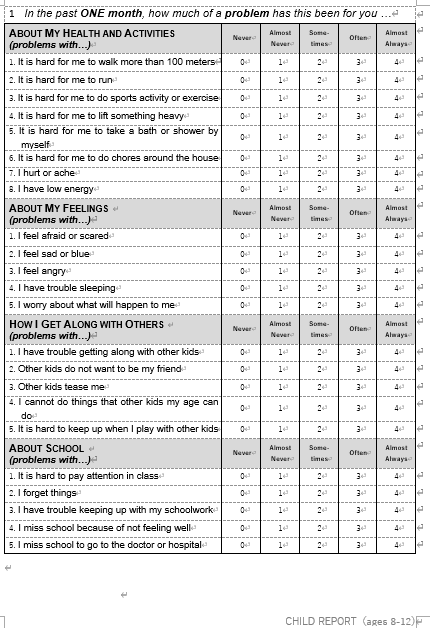


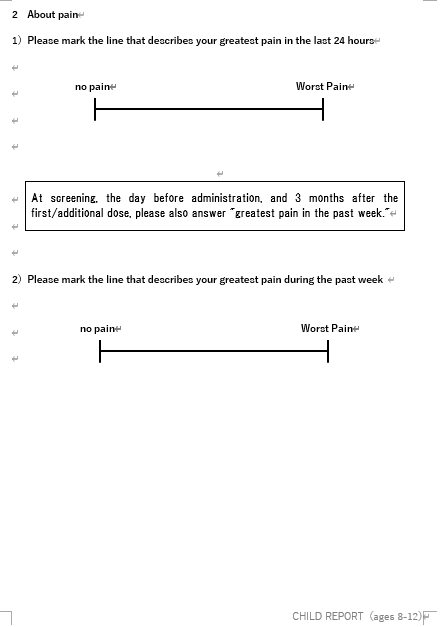


1. For the patient himself/herself (13-14 years old)


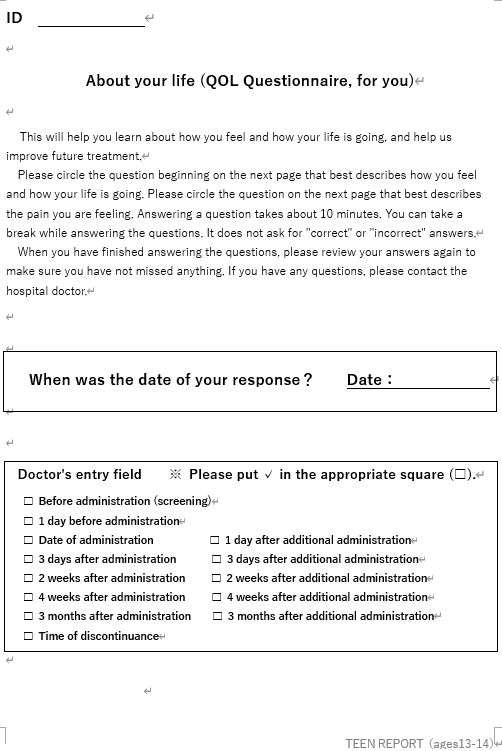


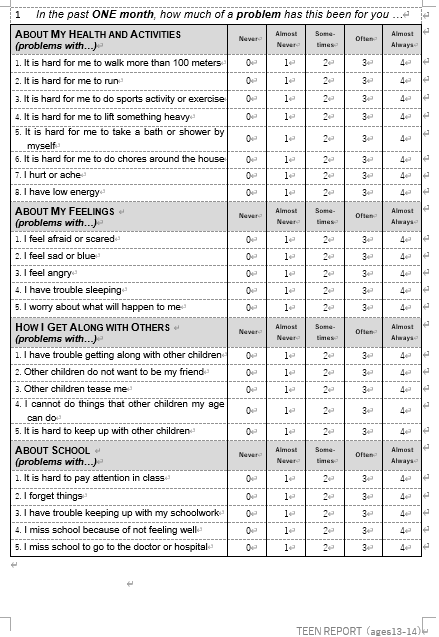


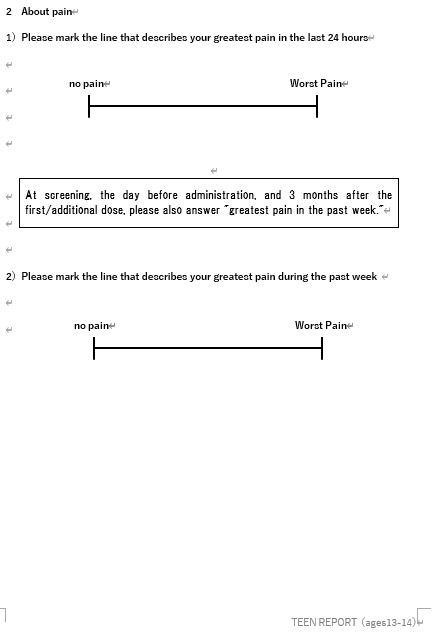


1. For the patient himself/herself (15 years and older)


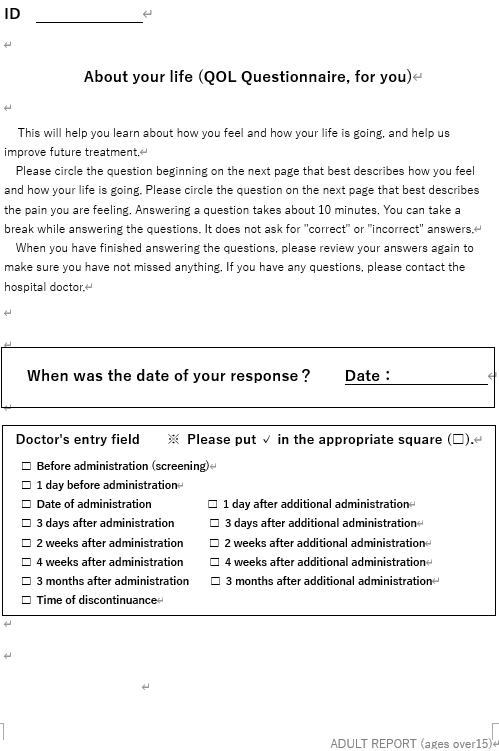


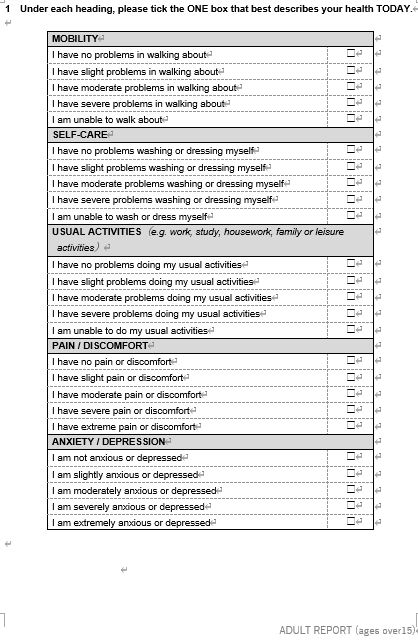


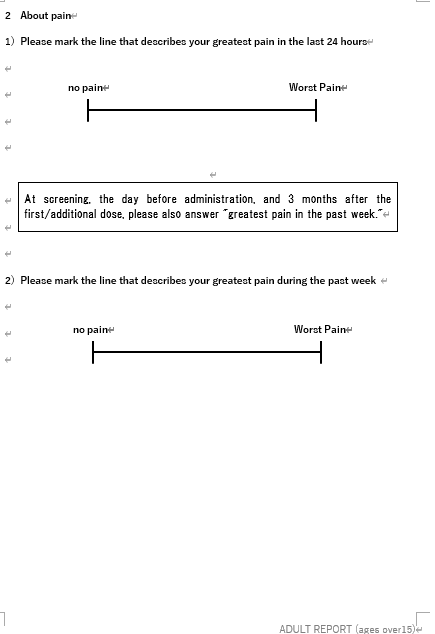


1. For parents (0-year-old patients)


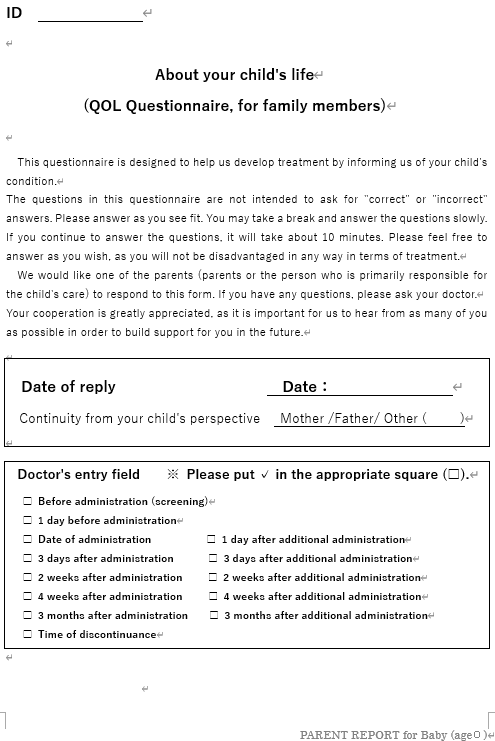


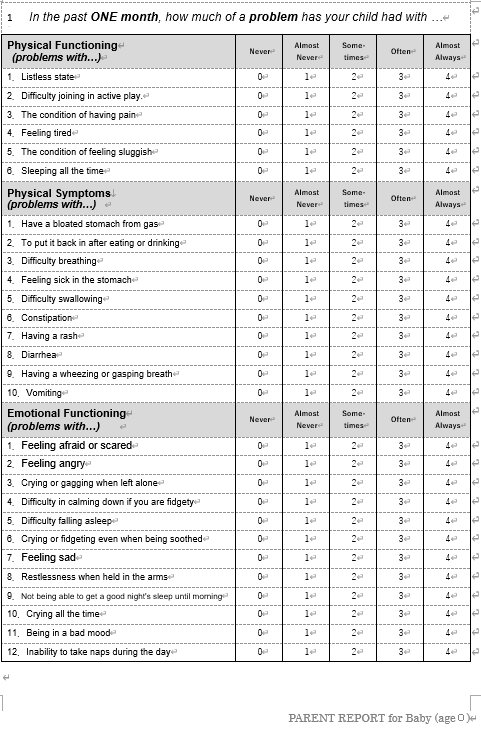


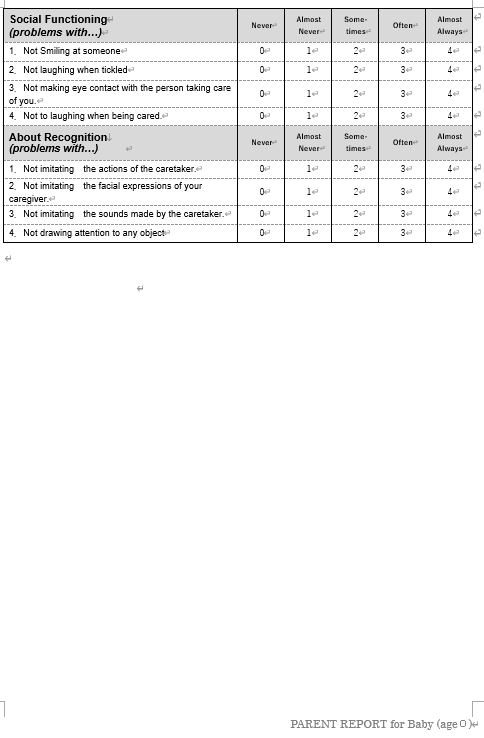


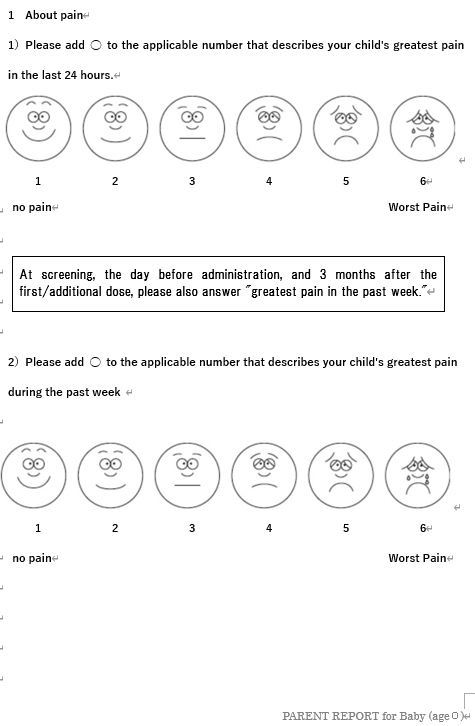


(8) For parents (1 year old patients)


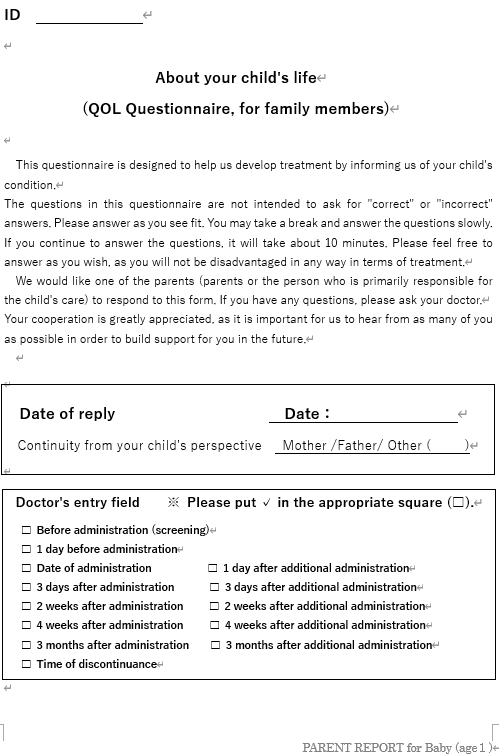


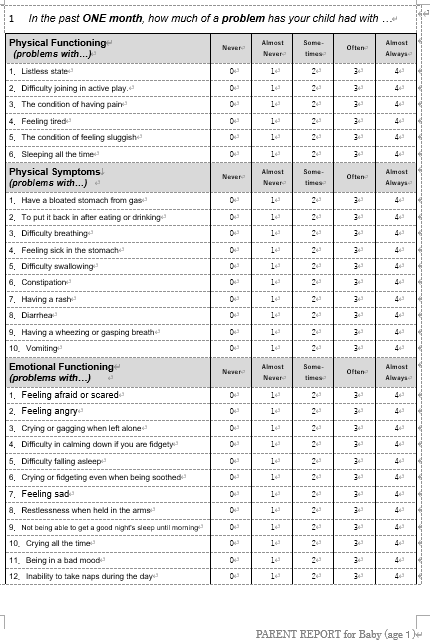


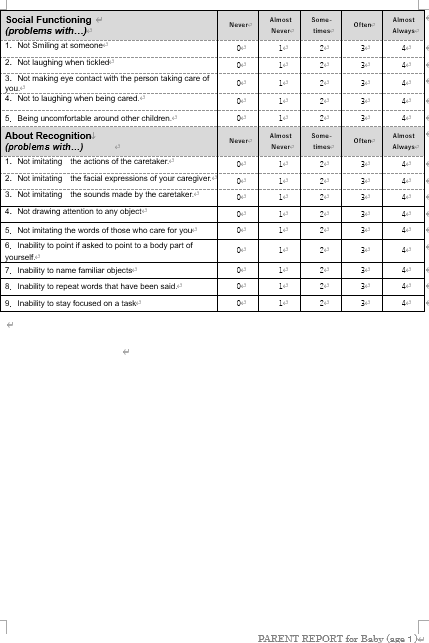


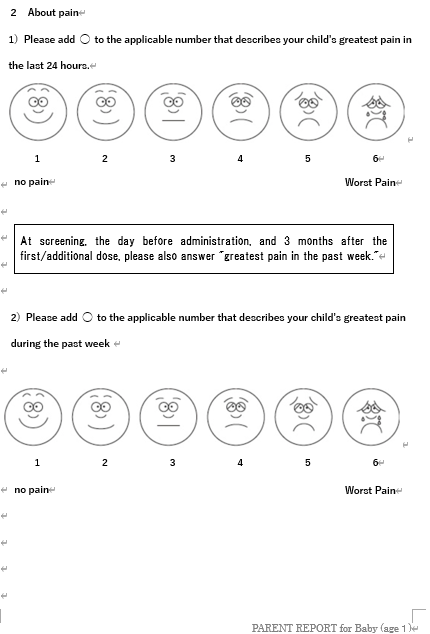


(9) For parents (2-4 years old patients)


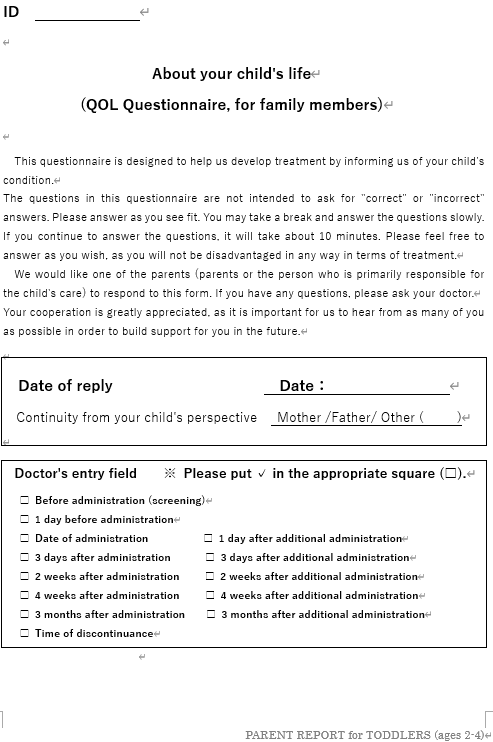


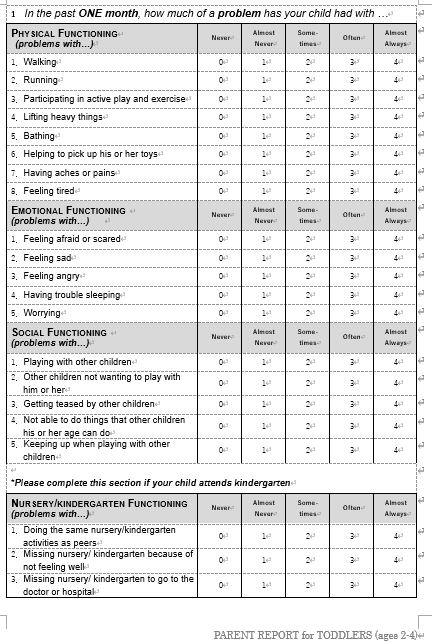


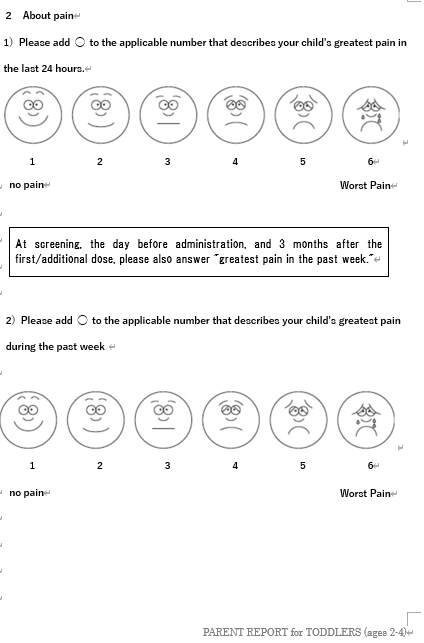


(10) For parents (5-years-old patients)


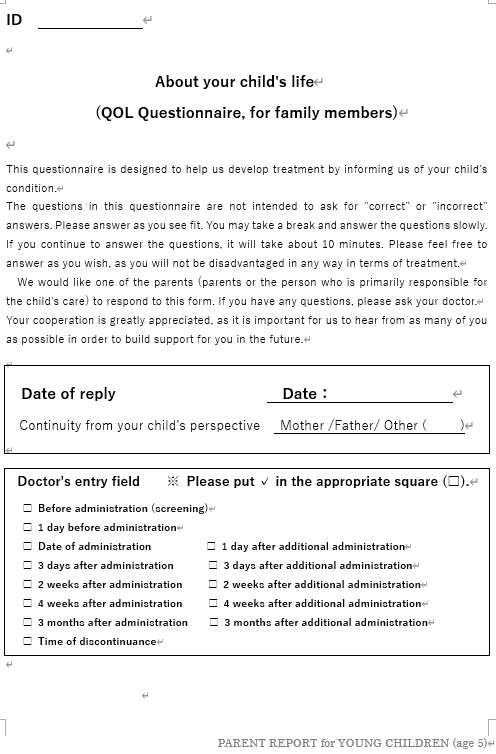


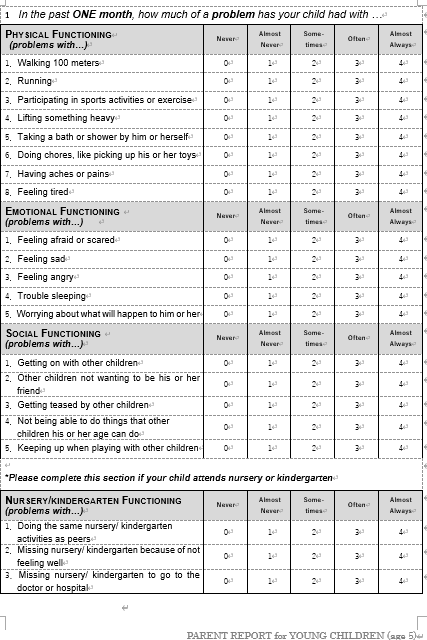


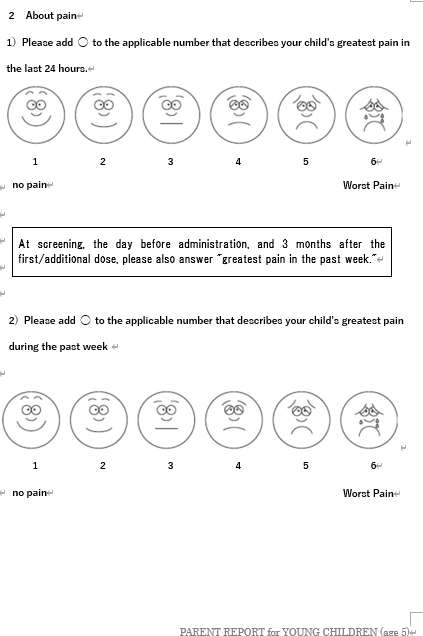


(11) For parents (6-7 years old patients)


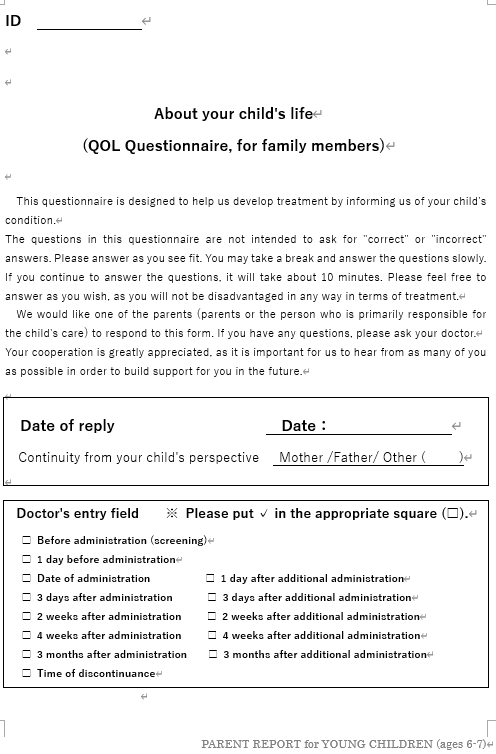


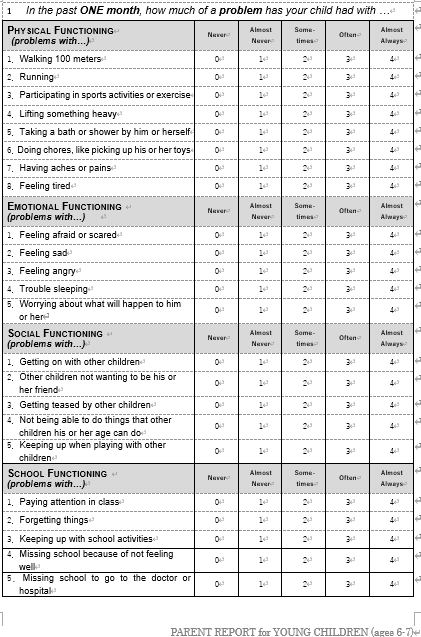


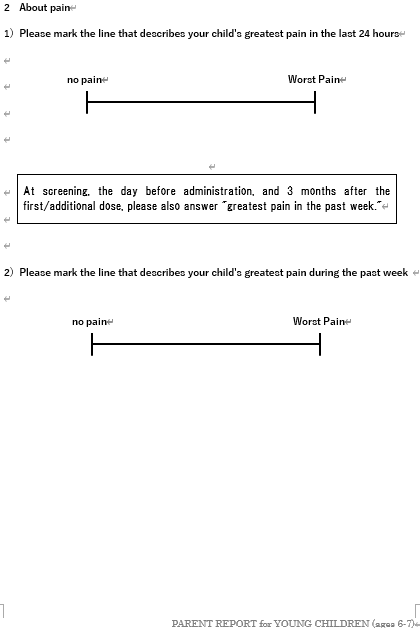


(12) For parents (patients 8-14 years old)


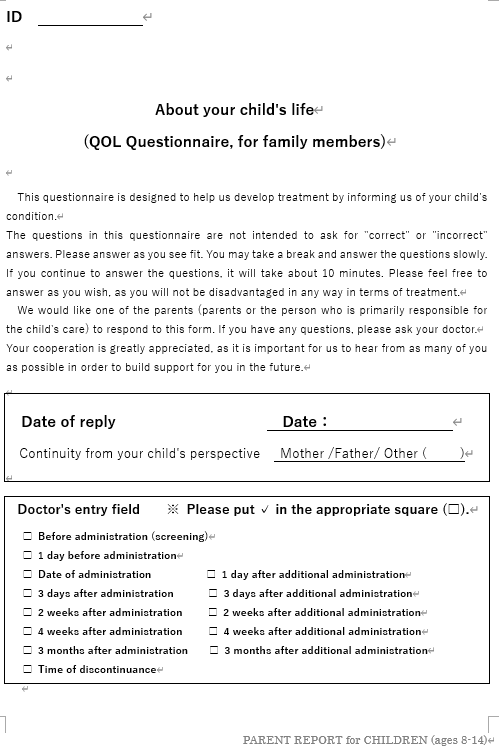


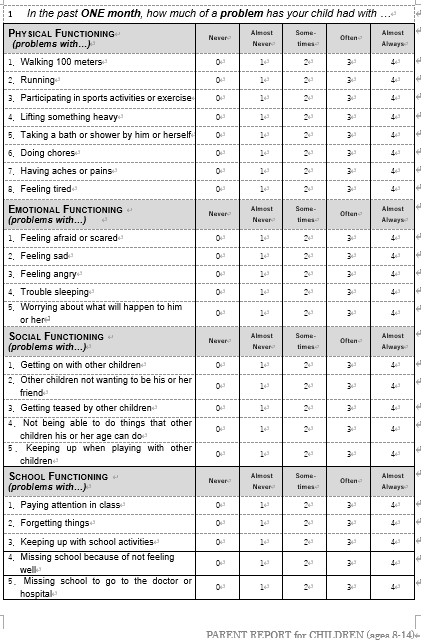


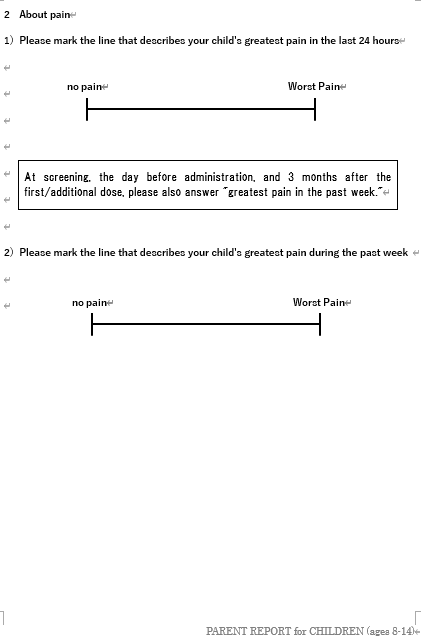


Revision History

| version number | Creation/Revision Date | Reason for Revision/Contents |
| --- | --- | --- |
| Version 1.01 | June 24, 2020 | newly enacted |
| Version 1.11 | July 17, 2020 | Change in the number of editions due to changes in the main body of the clinical trial protocol |
| Version 1.21 | September 28, 2020 | Creation of the questionnaire to be used |
| Version 1.31 | January 6, 2021 | Change in the number of editions due to changes in the main body of the clinical trial protocol |
| Version 1.41 | April 1, 2021 | Addition of "last week" survey for pain assessment, new version number |
| Version 1.51 | June 18, 2021 | Change in the number of editions due to changes in the main body of the clinical trial protocol |
| Version 1.61 | October 21, 2021 | Change in the number of editions due to changes in the main body of the clinical trial protocol |
| Version 1.71 | October 26, 2022 | Change in the number of editions due to changes in the main body of the clinical trial protocol |
| Version 1.81 | December 7, 2022 | Change in the number of editions due to changes in the main body of the clinical trial protocol |
