# Supplementary material for: Effect and safety of ethanolamine oleate in sclerotherapy in patients with difficult-to-resect venous malformations: A multicenter, single-arm study
Source: PLoS One. 2025 Jan 31;20(1):e0303130. doi: 10.1371/journal.pone.0303130 (PMC11785324; doi:10.1371/journal.pone.0303130)
Supplement: S1 Table — (PDF) [file pone.0303130.s004.pdf]

Reduction rate (%) of venous malformations
Analysis object: FAS

Lesion	Case number	PPS	Gender	Age (years)	Subgroup	Point in time	Analysis point in time	Volume (cm^3) Average value	Reduction rate (%)	Reduction rate 20% or more
Cystic lesion	Kyorin-01	Y	Male	6	6 to 11 years old	Before administration	Baseline	26.75	-	-
						3months after administration	3months after administration	19.95	25.42	Y
Cystic lesion	Kyorin-02	Y	Female	31	15 years and older	Before administration	Baseline	34.65	-	-
						3months after administration	3months after administration	9.05	73.88	Y
Cystic lesion	Kyorin-03	Y	Male	17	15 years and older	Before administration	Baseline	18.80	-	-
						3months after administration	3months after administration	7.80	58.51	Y
Cystic lesion	Junten-01	Y	Female	3	Under 5 years old	Before administration	Baseline	25.05	-	-
						3months after administration	3months after administration	24.50	2.20	N
Cystic lesion	Junten-02	Y	Male	14	12 to 14 years old	Before administration	Baseline	72.95	-	-
						3months after administration	3months after administration	44.50	39.00	Y
Cystic lesion	Junten-03	Y	Male	11	6 to 11 years old	Before administration	Baseline	34.00	-	-
						3months after administration	3months after administration	10.95	67.79	Y
Cystic lesion	Junten-04	Y	Male	8	6 to 11 years old	Before administration	Baseline	22.00	-	-
						3months after administration	3months after administration	4.30	80.45	Y
Cystic lesion	Junten-05	Y	Female	5	Under 5 years old	Before administration	Baseline	28.05	-	-
						3months after administration	3months after administration	1.00	96.43	Y
Cystic lesion	Kobe-01	Y	Female	78	15 years and older	Before administration	Baseline	19.95	-	-
						3months after administration	3months after administration	6.35	68.17	Y
Cystic lesion	Kobe-03	Y	Male	5	Under 5 years old	Before administration	Baseline	17.30	-	-
						3months after administration	3months after administration	1.05	93.93	Y
Cystic lesion	Kobe-09	Y	Male	8	6 to 11 years old	Before administration	Baseline	9.30	-	-
						3months after administration	3months after administration	1.90	79.57	Y
Cystic lesion	Kobe-11	Y	Male	44	15 years and older	Before administration	Baseline	8.70	-	-
						3months after administration	3months after administration	1.70	80.46	Y
Cystic lesion	Osaka-03	Y	Female	50	15 years and older	Before administration	Baseline	4.80	-	-
						3months after administration	3months after administration	2.45	48.96	Y
Cystic lesion	Osaka-06	Y	Male	5	Under 5 years old	Before administration	Baseline	44.80	-	-
						3months after administration	3months after administration	28.25	36.94	Y
Cystic lesion	Tokyo-01	Y	Female	26	15 years and older	Before administration	Baseline	90.30	-	-
						3months after administration	3months after administration	107.65	-19.21	N

Reduction rate (%) of venous malformations
Analysis object: FAS

Lesion	Case number	PPS	Gender	Age (years)	Subgroup	Point in time	Analysis point in time	Volume (cm^3) Average value	Reduction rate (%)	Reduction rate 20% or more
Cystic lesion	Tokyo-04	Y	Male	33	15 years and older	Before administration	Baseline	27.75	-	-
						3months after administration	3months after administration	3.50	87.39	Y
Cystic lesion	Tokyo-05	Y	Male	11	6 to 11 years old	Before administration	Baseline	2.25	-	-
						3months after administration	3months after administration	4.20	-86.67	N
Cystic lesion	Shinshu-02	Y	Female	37	15 years and older	Before administration	Baseline	34.10	-	-
						3months after administration	3months after administration	6.85	79.91	Y
Cystic lesion	Keio-01	Y	Female	27	15 years and older	Before administration	Baseline	131.20	-	-
						3months after administration	3months after administration	125.00	4.73	N
Cystic lesion	Keio-02	Y	Male	39	15 years and older	Before administration	Baseline	38.95	-	-
						3months after administration	3months after administration	43.10	-10.65	N
Cystic lesion	Keio-03	Y	Female	16	15 years and older	Before administration	Baseline	34.15	-	-
						3months after administration	3months after administration	29.05	14.93	N
Cystic lesion	Keio-04	Y	Male	22	15 years and older	Before administration	Baseline	29.95	-	-
						3months after administration	3months after administration	3.25	89.15	Y
Diffuse lesion	Kyorin-04	Y	Male	24	15 years and older	Before administration	Baseline	428.90	-	-
						3months after administration	3months after administration	466.20	-8.70	N
Diffuse lesion	Kyorin-05	Y	Female	22	15 years and older	Before administration	Baseline	34.70	-	-
						3months after administration	3months after administration	37.40	-7.78	N
Diffuse lesion	Kyorin-06	Y	Male	14	12 to 14 years old	Before administration	Baseline	96.65	-	-
						3months after administration	3months after administration	58.65	39.32	Y
Diffuse lesion	Kyorin-07	Y	Female	6	6 to 11 years old	Before administration	Baseline	62.95	-	-
						3months after administration	3months after administration	45.35	27.96	Y
Diffuse lesion	Kyorin-08	Y	Female	26	15 years and older	Before administration	Baseline	68.00	-	-
						3months after administration	3months after administration	66.20	2.65	N
Diffuse lesion	Kobe-02	Y	Female	16	15 years and older	Before administration	Baseline	407.40	-	-
						3months after administration	3months after administration	357.25	12.31	N
Diffuse lesion	Kobe-04	Y	Male	32	15 years and older	Before administration	Baseline	44.95	-	-
						3months after administration	3months after administration	39.75	11.57	N
Diffuse lesion	Kobe-05	Y	Female	56	15 years and older	Before administration	Baseline	3.85	-	-
						3months after administration	3months after administration	1.60	58.44	Y

Reduction rate (%) of venous malformations
Analysis object: FAS

Lesion	Case number	PPS	Gender	Age (years)	Subgroup	Point in time	Analysis point in time	Volume (cm^3) Average value	Reduction rate (%)	Reduction rate 20% or more
Diffuse lesion	Kobe-06	Y	Female	53	15 years and older	Before administration	Baseline	11.05	−	−
						3months after administration	3months after administration	7.50	32.13	Y
Diffuse lesion	Kobe-08	Y	Female	12	12 to 14 years old	Before administration	Baseline	10.75	−	−
						3months after administration	3months after administration	6.90	35.81	Y
Diffuse lesion	Kobe-10	Y	Male	13	12 to 14 years old	Before administration	Baseline	61.25	−	−
						3months after administration	3months after administration	43.60	28.82	Y
Diffuse lesion	Seiiku-01	Y	Male	5	Under 5 years old	Before administration	Baseline	28.15	−	−
						3months after administration	3months after administration	28.55	-1.42	N
Diffuse lesion	Osaka-01	Y	Male	25	15 years and older	Before administration	Baseline	297.75	−	−
						3months after administration	3months after administration	286.30	3.85	N
Diffuse lesion	Osaka-02	Y	Male	6	6 to 11 years old	Before administration	Baseline	123.70	−	−
						3months after administration	3months after administration	106.75	13.70	N
Diffuse lesion	Osaka-04	Y	Male	25	15 years and older	Before administration	Baseline	74.20	−	−
						3months after administration	3months after administration	51.45	30.66	Y
Diffuse lesion	Osaka-05	Y	Female	17	15 years and older	Before administration	Baseline	35.15	−	−
						3months after administration	3months after administration	26.20	25.46	Y
Diffuse lesion	Tokyo-02	Y	Female	16	15 years and older	Before administration	Baseline	21.55	−	−
						3months after administration	3months after administration	8.35	61.25	Y
Diffuse lesion	Tokyo-03	Y	Female	59	15 years and older	Before administration	Baseline	93.00	−	−
						3months after administration	3months after administration	117.40	-26.24	N
Diffuse lesion	Shinshu-01	Y	Female	14	12 to 14 years old	Before administration	Baseline	143.80	−	−
						3months after administration	3months after administration	118.55	17.56	N
Diffuse lesion	Shinshu-03	Y	Female	52	15 years and older	Before administration	Baseline	5.60	−	−
						3months after administration	3months after administration	7.10	-26.79	N
Diffuse lesion	Shinshu-04	Y	Male	10	6 to 11 years old	Before administration	Baseline	91.00	−	−
						3months after administration	3months after administration	91.85	-0.93	N
Diffuse lesion	Shinshu-05	Y	Female	10	6 to 11 years old	Before administration	Baseline	6.70	−	−
						3months after administration	3months after administration	1.20	82.09	Y
