# Supplementary material for: Effect and safety of ethanolamine oleate in sclerotherapy in patients with difficult-to-resect venous malformations: A multicenter, single-arm study
Source: PLoS One. 2025 Jan 31;20(1):e0303130. doi: 10.1371/journal.pone.0303130 (PMC11785324; doi:10.1371/journal.pone.0303130)
Supplement: S2 Table — (PDF) [file pone.0303130.s005.pdf]

Target lesion volume
Analysis object: FAS

												Remeasurement			
								Volume of venous malformation (cm^3)				Volume of venous malformation			
Lesion	Case number	PPS	Gender	Age (years)	Subgroup	Point in time	Analysis point in time	Central Judgment Committee Member 1	Central Judgment Committee Member 2	Difference (%)	Mean value	Central Judgment Committee Member 1	Central Judgment Committee Member 2	Difference (%)	Mean value
Cystic lesion	Kyorin-01	Y	Male	6	6 to 11 years old	Before administration	Baseline	41.5	27.4	34.0	34.45	27.0	26.5	1.9	26.75
						3 months after administration	3 months after administration	57.7	19.2	66.7	38.45	20.3	19.6	3.4	19.95
Cystic lesion	Kyorin-02	Y	Female	31	15 years and older	Before administration	Baseline	35.6	31.6	11.2	33.60	35.2	34.1	3.1	34.65
						3 months after administration	3 months after administration	9.3	8.8	5.4	9.05	－	－	－	－
Cystic lesion	Kyorin-03	Y	Male	17	15 years and older	Before administration	Baseline	19.6	18.0	8.2	18.80	－	－	－	－
						3 months after administration	3 months after administration	8.2	7.4	9.8	7.80	－	－	－	－
Cystic lesion	Junten-01	Y	Female	3	Under 5 years old	Before administration	Baseline	25.2	24.9	1.2	25.05	－	－	－	－
						3 months after administration	3 months after administration	24.6	24.4	0.8	24.50	－	－	－	－
Cystic lesion	Junten-02	Y	Male	14	12 to 14 years old	Before administration	Baseline	73.6	72.3	1.8	72.95	－	－	－	－
						3 months after administration	3 months after administration	44.9	44.1	1.8	44.50	－	－	－	－
Cystic lesion	Junten-03	Y	Male	11	6 to 11 years old	Before administration	Baseline	34.8	33.2	4.6	34.00	－	－	－	－
						3 months after administration	3 months after administration	11.4	10.5	7.9	10.95	－	－	－	－
Cystic lesion	Junten-04	Y	Male	8	6 to 11 years old	Before administration	Baseline	22.0	22.0	0.0	22.00	－	－	－	－
						3 months after administration	3 months after administration	4.3	4.3	0.0	4.30	－	－	－	－
Cystic lesion	Junten-05	Y	Female	5	Under 5 years old	Before administration	Baseline	28.4	27.7	2.5	28.05	－	－	－	－
						3 months after administration	3 months after administration	1.0	0.9	10.0	0.95	1.0	1.0	0.0	1.00
Cystic lesion	Kobe-01	Y	Female	78	15 years and older	Before administration	Baseline	20.5	19.4	5.4	19.95	－	－	－	－
						3 months after administration	3 months after administration	6.4	6.3	1.6	6.35	－	－	－	－
Cystic lesion	Kobe-03	Y	Male	5	Under 5 years old	Before administration	Baseline	17.5	17.1	2.3	17.30	－	－	－	－
						3 months after administration	3 months after administration	1.1	1.0	9.1	1.05	－	－	－	－
Cystic lesion	Kobe-09	Y	Male	8	6 to 11 years old	Before administration	Baseline	9.4	9.2	2.1	9.30	－	－	－	－
						3 months after administration	3 months after administration	1.9	1.9	0.0	1.90	－	－	－	－
Cystic lesion	Kobe-11	Y	Male	44	15 years and older	Before administration	Baseline	8.9	8.5	4.5	8.70	－	－	－	－
						3 months after administration	3 months after administration	1.7	1.7	0.0	1.70	－	－	－	－
Cystic lesion	Osaka-03	Y	Female	50	15 years and older	Before administration	Baseline	4.9	4.7	4.1	4.80	－	－	－	－
						3 months after administration	3 months after administration	2.5	2.4	4.0	2.45	－	－	－	－
Cystic lesion	Osaka-06	Y	Male	5	Under 5 years old	Before administration	Baseline	44.8	44.8	0.0	44.80	－	－	－	－
						3 months after administration	3 months after administration	28.3	28.2	0.4	28.25	－	－	－	－
Cystic lesion	Tokyo-01	Y	Female	26	15 years and older	Before administration	Baseline	97.4	86.5	11.2	91.95	93.8	86.8	7.5	90.30
						3 months after administration	3 months after administration	108.4	106.9	1.4	107.65	－	－	－	－

Target lesion volume
Analysis object: FAS

Lesion	Case number	PPS	Gender	Age (years)	Subgroup	Point in time	Analysis point in time	Volume of venous malformation (cm^3) Central Judgment Committee Member 1	Central Judgment Committee Member 2	Difference (%)	Mean value	Remeasurement Volume of venous malformation Central Judgment Committee Member 1	Central Judgment Committee Member 2	Difference (%)	Mean value
Cystic lesion	Tokyo-04	Y	Male	33	15 years and older	Before administration	Baseline	27.9	27.6	1.1	27.75	–	–	–	–
						3 months after administration	3 months after administration	3.6	3.4	5.6	3.50	–	–	–	–
Cystic lesion	Tokyo-05	Y	Male	11	6 to 11 years old	Before administration	Baseline	2.3	2.2	4.3	2.25	–	–	–	–
						3 months after administration	3 months after administration	4.2	4.2	0.0	4.20	–	–	–	–
Cystic lesion	Shinshu-02	Y	Female	37	15 years and older	Before administration	Baseline	34.2	34.0	0.6	34.10	–	–	–	–
						3 months after administration	3 months after administration	7.1	6.6	7.0	6.85	–	–	–	–
Cystic lesion	Keio-01	Y	Female	27	15 years and older	Before administration	Baseline	133.5	128.9	3.4	131.20	–	–	–	–
						3 months after administration	3 months after administration	128.0	122.0	4.7	125.00	–	–	–	–
Cystic lesion	Keio-02	Y	Male	39	15 years and older	Before administration	Baseline	41.8	32.0	23.4	36.90	39.8	38.1	4.3	38.95
						3 months after administration	3 months after administration	43.6	42.6	2.3	43.10	–	–	–	–
Cystic lesion	Keio-03	Y	Female	16	15 years and older	Before administration	Baseline	34.2	34.1	0.3	34.15	–	–	–	–
						3 months after administration	3 months after administration	29.1	29.0	0.3	29.05	–	–	–	–
Cystic lesion	Keio-04	Y	Male	22	15 years and older	Before administration	Baseline	30.0	29.9	0.3	29.95	–	–	–	–
						3 months after administration	3 months after administration	3.3	3.2	3.0	3.25	–	–	–	–
Diffuse lesion	Kyorin-04	Y	Male	24	15 years and older	Before administration	Baseline	437.8	420.0	4.1	428.90	–	–	–	–
						3 months after administration	3 months after administration	476.3	456.1	4.2	466.20	–	–	–	–
Diffuse lesion	Kyorin-05	Y	Female	22	15 years and older	Before administration	Baseline	35.6	33.8	5.1	34.70	–	–	–	–
						3 months after administration	3 months after administration	38.9	35.9	7.7	37.40	–	–	–	–
Diffuse lesion	Kyorin-06	Y	Male	14	12 to 14 years old	Before administration	Baseline	98.7	94.6	4.2	96.65	–	–	–	–
						3 months after administration	3 months after administration	59.2	58.1	1.9	58.65	–	–	–	–
Diffuse lesion	Kyorin-07	Y	Female	6	6 to 11 years old	Before administration	Baseline	63.0	62.9	0.2	62.95	–	–	–	–
						3 months after administration	3 months after administration	45.8	44.9	2.0	45.35	–	–	–	–
Diffuse lesion	Kyorin-08	Y	Female	26	15 years and older	Before administration	Baseline	69.0	67.0	2.9	68.00	–	–	–	–
						3 months after administration	3 months after administration	66.5	65.9	0.9	66.20	–	–	–	–
Diffuse lesion	Kobe-02	Y	Female	16	15 years and older	Before administration	Baseline	412.1	402.7	2.3	407.40	–	–	–	–
						3 months after administration	3 months after administration	365.1	349.4	4.3	357.25	–	–	–	–
Diffuse lesion	Kobe-04	Y	Male	32	15 years and older	Before administration	Baseline	45.8	44.1	3.7	44.95	–	–	–	–
						3 months after administration	3 months after administration	39.9	39.6	0.8	39.75	–	–	–	–
Diffuse lesion	Kobe-05	Y	Female	56	15 years and older	Before administration	Baseline	4.7	4.0	14.9	4.35	3.9	3.8	2.6	3.85
						3 months after administration	3 months after administration	1.7	1.5	11.8	1.60	–	–	–	–

Target lesion volume
Analysis object: FAS

Lesion	Case number	PPS	Gender	Age (years)	Subgroup	Point in time	Analysis point in time	Volume of venous malformation (cm^3) Central Judgment Committee Member 1	Central Judgment Committee Member 2	Difference (%)	Mean value	Remeasurement Volume of venous malformation Central Judgment Committee Member 1	Central Judgment Committee Member 2	Difference (%)	Mean value
Diffuse lesion	Kobe-06	Y	Female	53	15 years and older	Before administration	Baseline	11.5	10.6	7.8	11.05	–	–	–	–
						3 months after administration	3 months after administration	7.7	7.3	5.2	7.50	–	–	–	–
Diffuse lesion	Kobe-08	Y	Female	12	12 to 14 years old	Before administration	Baseline	10.9	10.6	2.8	10.75	–	–	–	–
						3 months after administration	3 months after administration	6.9	6.9	0.0	6.90	–	–	–	–
Diffuse lesion	Kobe-10	Y	Male	13	12 to 14 years old	Before administration	Baseline	64.0	58.5	8.6	61.25	–	–	–	–
						3 months after administration	3 months after administration	43.8	43.4	0.9	43.60	–	–	–	–
Diffuse lesion	Seiiku-01	Y	Male	5	Under 5 years old	Before administration	Baseline	28.5	27.8	2.5	28.15	–	–	–	–
						3 months after administration	3 months after administration	29.3	27.8	5.1	28.55	–	–	–	–
Diffuse lesion	Osaka-01	Y	Male	25	15 years and older	Before administration	Baseline	302.0	293.5	2.8	297.75	–	–	–	–
						3 months after administration	3 months after administration	287.1	285.5	0.6	286.30	–	–	–	–
Diffuse lesion	Osaka-02	Y	Male	6	6 to 11 years old	Before administration	Baseline	124.1	123.3	0.6	123.70	–	–	–	–
						3 months after administration	3 months after administration	107.6	105.9	1.6	106.75	–	–	–	–
Diffuse lesion	Osaka-04	Y	Male	25	15 years and older	Before administration	Baseline	74.9	73.5	1.9	74.20	–	–	–	–
						3 months after administration	3 months after administration	52.6	50.3	4.4	51.45	–	–	–	–
Diffuse lesion	Osaka-05	Y	Female	17	15 years and older	Before administration	Baseline	35.2	35.1	0.3	35.15	–	–	–	–
						3 months after administration	3 months after administration	27.1	25.3	6.6	26.20	–	–	–	–
Diffuse lesion	Tokyo-02	Y	Female	16	15 years and older	Before administration	Baseline	21.7	21.4	1.4	21.55	–	–	–	–
						3 months after administration	3 months after administration	8.7	8.0	8.0	8.35	–	–	–	–
Diffuse lesion	Tokyo-03	Y	Female	59	15 years and older	Before administration	Baseline	94.6	91.4	3.4	93.00	–	–	–	–
						3 months after administration	3 months after administration	119.7	115.1	3.8	117.40	–	–	–	–
Diffuse lesion	Shinshu-01	Y	Female	14	12 to 14 years old	Before administration	Baseline	145.4	142.2	2.2	143.80	–	–	–	–
						3 months after administration	3 months after administration	121.5	115.6	4.9	118.55	–	–	–	–
Diffuse lesion	Shinshu-03	Y	Female	52	15 years and older	Before administration	Baseline	5.7	5.5	3.5	5.60	–	–	–	–
						3 months after administration	3 months after administration	7.3	6.9	5.5	7.10	–	–	–	–
Diffuse lesion	Shinshu-04	Y	Male	10	6 to 11 years old	Before administration	Baseline	92.6	89.4	3.5	91.00	–	–	–	–
						3 months after administration	3 months after administration	93.2	90.5	2.9	91.85	–	–	–	–
Diffuse lesion	Shinshu-05	Y	Female	10	6 to 11 years old	Before administration	Baseline	7.0	6.4	8.6	6.70	–	–	–	–
						3 months after administration	3 months after administration	1.2	1.2	0.0	1.20	–	–	–	–
