# Supplementary material for: Effect and safety of ethanolamine oleate in sclerotherapy in patients with difficult-to-resect venous malformations: A multicenter, single-arm study
Source: PLoS One. 2025 Jan 31;20(1):e0303130. doi: 10.1371/journal.pone.0303130 (PMC11785324; doi:10.1371/journal.pone.0303130)
Supplement: S3 Table — (PDF) [file pone.0303130.s006.pdf]

Pain scores associated with target lesion
Analysis object: FAS

										The patient him/herself						guardian					
												Maximum pain in 24 hours		Maximum pain for 1 week				Maximum pain in 24 hours		Maximum pain for 1 week	
Lesion	Case number	PPS	Gender	Age (years)	Subgroup	Guardian	Point in time	Analysis point in time	Rating Scale	Implementation Yes or	Date of reply	Measured value	(−1)x Amount of change	Measured value	(−1)x Amount of change	Implementation Yes or No	Date of reply	Measured value	(−1)x Amount of change	Measured value	(−1)x Amount of change
Cystic lesion	Kyorin−01	Y	Male	6	6 to 11 years old	mother	Before administration	screening	VAS	Yes	2021−01−15	0	−	−	−	Yes	2021−01−15	0	−	−	−
							1 day before administration	baseline		Yes	2021−02−08	21	−	−	−	Yes	2021−02−08	18	−	−	−
							1 day after administration	1 day after administration		Yes	2021−02−10	81	−60	−	−	Yes	2021−02−10	100	−82	−	−
							3 days after administration	3 days after administration		Yes	2021−02−11	11	10	−	−	Yes	2021−02−11	9	9	−	−
							2 weeks after administration	2 weeks after administration		Yes	2021−02−19	0	21	−	−	Yes	2021−02−19	0	18	−	−
							4 weeks after administration	4 weeks after administration		Yes	2021−03−12	0	21	−	−	Yes	2021−03−12	0	18	−	−
							3 months after administration	3 months after administration		Yes	2021−05−07	0	21	−	−	Yes	2021−05−07	0	18	−	−
Cystic lesion	Kyorin−02	Y	Female	31	15 years and older	−	Before administration	screening	VAS	Yes	2021−03−05	0	−	−	−	−	−	−	−	−	−
							1 day before administration	baseline		Yes	2021−03−24	0	−	−	−	−	−	−	−	−	−
							1 day after administration	1 day after administration		Yes	2021−03−26	65	−65	−	−	−	−	−	−	−	−
							3 days after administration	3 days after administration		Yes	2021−03−27	67	−67	−	−	−	−	−	−	−	−
							2 weeks after administration	2 weeks after administration		Yes	2021−04−09	29	−29	−	−	−	−	−	−	−	−
							4 weeks after administration	4 weeks after administration		Yes	2021−04−22	2	−2	−	−	−	−	−	−	−	−
							3 months after administration	3 months after administration		Yes	2021−06−18	0	0	0	−	−	−	−	−	−	−
Cystic lesion	Kyorin−03	Y	Male	17	15 years and older	−	Before administration	screening	VAS	Yes	2021−07−09	0	−	0	−	−	−	−	−	−	−
							1 day before administration	baseline		Yes	2021−07−19	0	−	0	−	−	−	−	−	−	−
							1 day after administration	1 day after administration		Yes	2021−07−21	34	−34	−	−	−	−	−	−	−	−
							3 days after administration	3 days after administration		Yes	2021−07−22	3	−3	−	−	−	−	−	−	−	−
							2 weeks after administration	2 weeks after administration		Yes	2021−08−06	2	−2	−	−	−	−	−	−	−	−
							4 weeks after administration	4 weeks after administration		Yes	2021−08−20	0	0	−	−	−	−	−	−	−	−
							3 months after administration	3 months after administration		Yes	2021−10−15	0	0	0	0	−	−	−	−	−	−
Cystic lesion	Junten−01	Y	Female	3	Under 5 years old	mother	Before administration	screening	Face scale	No	−	−	−	−	−	Yes	2021−02−18	2	−	−	−
							1 day before administration	baseline		No	−	−	−	−	−	Yes	2021−03−16	3	−	−	−
							1 day after administration	1 day after administration		No	−	−	−	−	−	Yes	2021−03−18	6	−3	−	−
							3 days after administration	3 days after administration		No	−	−	−	−	−	Yes	2021−03−19	6	−3	−	−
							2 weeks after administration	2 weeks after administration		No	−	−	−	−	−	Yes	2021−04−02	2	1	−	−
							4 weeks after administration	4 weeks after administration		No	−	−	−	−	−	Yes	2021−04−13	2	1	−	−
							3 months after administration	3 months after administration		No	−	−	−	−	−	Yes	2021−06−08	1	2	2	−
Cystic lesion	Junten−02	Y	Male	14	12 to 14 years old	mother	Before administration	screening	VAS	Yes	2021−09−07	11	−	12	−	Yes	2021−09−07	11	−	12	−
							1 day before administration	baseline		Yes	2021−09−29	3	−	3	−	Yes	2021−09−29	4	−	6	−
							1 day after administration	1 day after administration		Yes	2021−10−01	26	−23	−	−	Yes	2021−10−01	52	−48	−	−
							3 days after administration	3 days after administration		Yes	2021−10−02	4	−1	−	−	Yes	2021−10−02	34	−30	−	−
							2 weeks after administration	2 weeks after administration		Yes	2021−10−12	2	1	−	−	Yes	2021−10−12	0	4	−	−
							4 weeks after administration	4 weeks after administration		Yes	2021−10−26	1	2	−	−	Yes	2021−10−26	0	4	−	−
							3 months after administration	3 months after administration		Yes	2021−12−24	1	2	2	1	Yes	2021−12−24	0	4	0	6
Cystic lesion	Junten−03	Y	Male	11	6 to 11 years old	mother	Before administration	screening	VAS	Yes	2022−02−01	2	−	2	−	Yes	2022−02−01	0	−	0	−
							1 day before administration	baseline		Yes	2022−02−16	2	−	2	−	Yes	2022−02−16	0	−	0	−
							1 day after administration	1 day after administration		Yes	2022−02−18	23	−21	−	−	Yes	2022−02−18	46	−46	−	−
							3 days after administration	3 days after administration		Yes	2022−02−19	12	−10	−	−	Yes	2022−02−19	11	−11	−	−
							2 weeks after administration	2 weeks after administration		Yes	2022−03−04	2	0	−	−	Yes	2022−03−04	3	−3	−	−
							4 weeks after administration	4 weeks after administration		Yes	2022−03−15	2	0	−	−	Yes	2022−03−15	2	−2	−	−
							3 months after administration	3 months after administration		Yes	2022−05−17	0	2	0	2	Yes	2022−05−17	0	0	0	0

Pain scores associated with target lesion
Analysis object: FAS

										The patient him/herself						guardian					
												Maximum pain in 24 hours		Maximum pain for 1 week				Maximum pain in 24 hours		Maximum pain for 1 week	
Lesion	Case number	PPS	Gender	Age (years)	Subgroup	Guardian	Point in time	Analysis point in time	Rating Scale	Implementation Yes or	Date of reply	Measured value	(−1)x Amount of change	Measured value	(−1)x Amount of change	Implementation Yes or No	Date of reply	Measured value	(−1)x Amount of change	Measured value	(−1)x Amount of change
Cystic lesion	Junten−04	Y	Male	8	6 to 11 years old	mother	Before administration	screening	VAS	Yes	2022−07−05	0	−	0	−	Yes	2022−07−05	0	−	0	−
							1 day before administration	baseline		Yes	2022−07−27	0	−	0	−	Yes	2022−07−27	0	−	0	−
							1 day after administration	1 day after administration		Yes	2022−07−29	18	−18	−	−	Yes	2022−07−29	100	−100	−	−
							3 days after administration	3 days after administration		Yes	2022−07−30	14	−14	−	−	Yes	2022−07−30	31	−31	−	−
							2 weeks after administration	2 weeks after administration		Yes	2022−08−16	0	0	−	−	Yes	2022−08−16	0	0	−	−
							4 weeks after administration	4 weeks after administration		Yes	2022−08−30	0	0	−	−	Yes	2022−08−30	0	0	−	−
							3 months after administration	3 months after administration		Yes	2022−10−15	13	−13	9	−9	Yes	2022−10−15	6	−6	7	−7
Cystic lesion	Junten−05	Y	Female	5	Under 5 years old	mother	Before administration	screening	Face scale	Yes	2022−07−12	1	−	1	−	Yes	2022−07−12	1	−	2	−
							1 day before administration	baseline		Yes	2022−07−27	1	−	2	−	Yes	2022−07−27	1	−	3	−
							1 day after administration	1 day after administration		Yes	2022−07−29	3	−2	−	−	Yes	2022−07−29	5	−4	−	−
							3 days after administration	3 days after administration		Yes	2022−07−30	1	0	−	−	Yes	2022−07−30	1	0	−	−
							2 weeks after administration	2 weeks after administration		Yes	2022−08−16	1	0	−	−	Yes	2022−08−16	1	0	−	−
							4 weeks after administration	4 weeks after administration		Yes	2022−08−30	1	0	−	−	Yes	2022−08−30	1	0	−	−
							3 months after administration	3 months after administration		Yes	2022−10−15	1	0	1	1	Yes	2022−10−15	1	0	1	2
Cystic lesion	Kobe−01	Y	Female	78	15 years and older	−	Before administration	screening	VAS	Yes	2021−02−15	6	−	−	−	−	−	−	−	−	−
							1 day before administration	baseline		Yes	2021−03−02	8	−	−	−	−	−	−	−	−	−
							1 day after administration	1 day after administration		Yes	2021−03−04	83	−75	−	−	−	−	−	−	−	−
							3 days after administration	3 days after administration		Yes	2021−03−05	13	−5	−	−	−	−	−	−	−	−
							2 weeks after administration	2 weeks after administration		Yes	2021−03−16	11	−3	−	−	−	−	−	−	−	−
							4 weeks after administration	4 weeks after administration		Yes	2021−03−30	0	8	−	−	−	−	−	−	−	−
							3 months after administration	3 months after administration		Yes	2021−05−24	0	8	0	−	−	−	−	−	−	−
Cystic lesion	Kobe−03	Y	Male	5	Under 5 years old	mother	Before administration	screening	Face scale	Yes	2021−07−20	1	−	1	−	Yes	2021−07−20	1	−	1	−
							1 day before administration	baseline		Yes	2021−08−10	1	−	1	−	Yes	2021−08−10	1	−	1	−
							1 day after administration	1 day after administration		Yes	2021−08−12	6	−5	−	−	Yes	2021−08−12	6	−5	−	−
							3 days after administration	3 days after administration	VAS	Yes	2021−08−13	100	−	−	−	Yes	2021−08−13	100	−	−	−
							2 weeks after administration	2 weeks after administration		Yes	2021−08−24	13	−	−	−	Yes	2021−08−24	13	−	−	−
							4 weeks after administration	4 weeks after administration		Yes	2021−09−07	6	−	−	−	Yes	2021−09−07	3	−	−	−
							3 months after administration	3 months after administration	Face scale	Yes	2021−11−02	1	0	1	0	Yes	2021−11−02	1	0	1	0
Cystic lesion	Kobe−09	Y	Male	8	6 to 11 years old	mother	Before administration	screening	VAS	Yes	2022−07−26	63	−	65	−	Yes	2022−07−26	37	−	36	−
							1 day before administration	baseline		Yes	2022−08−09	0	−	19	−	Yes	2022−08−09	0	−	4	−
							1 day after administration	1 day after administration		Yes	2022−08−11	87	−87	−	−	Yes	2022−08−11	95	−95	−	−
							3 days after administration	3 days after administration		Yes	2022−08−12	64	−64	−	−	Yes	2022−08−12	70	−70	−	−
							2 weeks after administration	2 weeks after administration		Yes	2022−08−23	0	0	−	−	Yes	2022−08−23	0	0	−	−
							4 weeks after administration	4 weeks after administration		Yes	2022−09−06	0	0	−	−	Yes	2022−09−06	0	0	−	−
							3 months after administration	3 months after administration		Yes	2022−11−01	0	0	0	19	Yes	2022−11−01	0	0	0	4
Cystic lesion	Kobe−11	Y	Male	44	15 years and older	−	Before administration	screening	VAS	Yes	2023−01−10	35	−	54	−	−	−	−	−	−	−
							1 day before administration	baseline		Yes	2023−01−24	25	−	35	−	−	−	−	−	−	−
							1 day after administration	1 day after administration		Yes	2023−01−26	58	−33	−	−	−	−	−	−	−	−
							3 days after administration	3 days after administration		Yes	2023−01−27	36	−11	−	−	−	−	−	−	−	−
							2 weeks after administration	2 weeks after administration		Yes	2023−02−06	16	9	−	−	−	−	−	−	−	−
							4 weeks after administration	4 weeks after administration		Yes	2023−02−27	1	24	−	−	−	−	−	−	−	−
							3 months after administration	3 months after administration		Yes	2023−04−18	0	25	0	35	−	−	−	−	−	−

Pain scores associated with target lesion
Analysis object: FAS

												The patient him/herself				guardian					
												Maximum pain in 24 hours		Maximum pain for 1 week				Maximum pain in 24 hours		Maximum pain for 1 week	
Lesion	Case number	PPS	Gender	Age (years)	Subgroup	Guardian	Point in time	Analysis point in time	Rating Scale	Implementation Yes or	Date of reply	Measured value	(−1)x Amount of change	Measured value	(−1)x Amount of change	Implementation Yes or No	Date of reply	Measured value	(−1)x Amount of change	Measured value	(−1)x Amount of change
Cystic lesion	Osaka−03	Y	Female	50	15 years and older	−	Before administration	screening	VAS	Yes	2021−09−22	4	−	4	−	−	−	−	−	−	−
							1 day before administration	baseline		Yes	2021−10−18	0	−	0	−	−	−	−	−	−	−
							1 day after administration	1 day after administration		Yes	2021−10−20	31	−31	−	−	−	−	−	−	−	−
							3 days after administration	3 days after administration		Yes	2021−10−22	45	−45	−	−	−	−	−	−	−	−
							2 weeks after administration	2 weeks after administration		Yes	2021−11−04	14	−14	−	−	−	−	−	−	−	−
							4 weeks after administration	4 weeks after administration		Yes	2021−11−17	2	−2	−	−	−	−	−	−	−	−
							3 months after administration	3 months after administration		Yes	2022−01−12	0	0	0	0	−	−	−	−	−	−
Cystic lesion	Osaka−06	Y	Male	5	Under 5 years old	mother	Before administration	screening	Face scale	Yes	2022−04−28	1	−	1	−	Yes	2022−04−28	1	−	1	−
							1 day before administration	baseline		No	−	−	−	−	−	Yes	2022−05−23	1	−	1	−
							1 day after administration	1 day after administration		No	−	−	−	−	−	Yes	2022−05−25	4	−3	−	−
							3 days after administration	3 days after administration		No	−	−	−	−	−	Yes	2022−05−27	1	0	−	−
							2 weeks after administration	2 weeks after administration		No	−	−	−	−	−	Yes	2022−06−08	1	0	−	−
							4 weeks after administration	4 weeks after administration		No	−	−	−	−	−	Yes	2022−06−22	1	0	−	−
							3 months after administration	3 months after administration		No	−	−	−	−	−	Yes	2022−08−18	1	0	1	0
Cystic lesion	Tokyo−01	Y	Female	26	15 years and older	−	Before administration	screening	VAS	Yes	2021−02−25	24	−	−	−	−	−	−	−	−	−
							1 day before administration	baseline		Yes	2021−03−09	32	−	−	−	−	−	−	−	−	−
							1 day after administration	1 day after administration		Yes	2021−03−11	34	−2	−	−	−	−	−	−	−	−
							3 days after administration	3 days after administration		Yes	2021−03−12	37	−5	−	−	−	−	−	−	−	−
							2 weeks after administration	2 weeks after administration		Yes	2021−03−25	9	23	−	−	−	−	−	−	−	−
							4 weeks after administration	4 weeks after administration		Yes	2021−04−08	0	32	−	−	−	−	−	−	−	−
							3 months after administration	3 months after administration		Yes	2021−06−10	0	32	0	−	−	−	−	−	−	−
Cystic lesion	Tokyo−04	Y	Male	33	15 years and older	−	Before administration	screening	VAS	Yes	2022−05−12	0	−	0	−	−	−	−	−	−	−
							1 day before administration	baseline		Yes	2022−05−26	0	−	0	−	−	−	−	−	−	−
							1 day after administration	1 day after administration		Yes	2022−05−28	27	−27	−	−	−	−	−	−	−	−
							3 days after administration	3 days after administration		Yes	2022−05−30	0	0	−	−	−	−	−	−	−	−
							2 weeks after administration	2 weeks after administration		Yes	2022−06−09	0	0	−	−	−	−	−	−	−	−
							4 weeks after administration	4 weeks after administration		Yes	2022−06−23	0	0	−	−	−	−	−	−	−	−
							3 months after administration	3 months after administration		Yes	2022−08−18	0	0	0	0	−	−	−	−	−	−
Cystic lesion	Tokyo−05	Y	Male	11	6 to 11 years old	mother	Before administration	screening	VAS	Yes	2022−10−06	47	−	60	−	Yes	2022−10−06	57	−	81	−
							1 day before administration	baseline		Yes	2022−10−18	64	−	64	−	Yes	2022−10−18	57	−	59	−
							1 day after administration	1 day after administration		Yes	2022−10−20	79	−15	−	−	Yes	2022−10−20	98	−41	−	−
							3 days after administration	3 days after administration		Yes	2022−10−21	100	−36	−	−	Yes	2022−10−21	91	−34	−	−
							2 weeks after administration	2 weeks after administration		Yes	2022−11−04	3	61	−	−	Yes	2022−11−04	0	57	−	−
							4 weeks after administration	4 weeks after administration		Yes	2022−11−17	0	64	−	−	Yes	2022−11−17	2	55	−	−
							3 months after administration	3 months after administration		Yes	2023−01−05	0	64	0	64	Yes	2023−01−05	0	57	0	59
Cystic lesion	Shinshu−02	Y	Female	37	15 years and older	−	Before administration	screening	VAS	Yes	2021−07−27	81	−	92	−	−	−	−	−	−	−
							1 day before administration	baseline		Yes	2021−08−17	35	−	51	−	−	−	−	−	−	−
							1 day after administration	1 day after administration		Yes	2021−08−19	98	−63	−	−	−	−	−	−	−	−
							3 days after administration	3 days after administration		Yes	2021−08−20	44	−9	−	−	−	−	−	−	−	−
							2 weeks after administration	2 weeks after administration		Yes	2021−08−31	24	11	−	−	−	−	−	−	−	−
							4 weeks after administration	4 weeks after administration		Yes	2021−09−14	2	33	−	−	−	−	−	−	−	−
							3 months after administration	3 months after administration		Yes	2021−11−09	0	35	0	51	−	−	−	−	−	−

Pain scores associated with target lesion
Analysis object: FAS

										The patient him/herself						guardian					
												Maximum pain in 24 hours		Maximum pain for 1 week				Maximum pain in 24 hours		Maximum pain for 1 week	
Lesion	Case number	PPS	Gender	Age (years)	Subgroup	Guardian	Point in time	Analysis point in time	Rating Scale	Implementation Yes or	Date of reply	Measured value	(−1)x Amount of change	Measured value	(−1)x Amount of change	Implementation Yes or No	Date of reply	Measured value	(−1)x Amount of change	Measured value	(−1)x Amount of change
Cystic lesion	Keio-01	Y	Female	27	15 years and older	–	Before administration	screening	VAS	Yes	2021-03-29	0	–	–	–	–	–	–	–	–	–
							1 day before administration	baseline		Yes	2021-04-07	0	–	–	–	–	–	–	–	–	–
							1 day after administration	1 day after administration		Yes	2021-04-09	51	-51	–	–	–	–	–	–	–	–
							3 days after administration	3 days after administration		Yes	2021-04-10	15	-15	–	–	–	–	–	–	–	–
							2 weeks after administration	2 weeks after administration		Yes	2021-04-21	10	-10	–	–	–	–	–	–	–	–
							4 weeks after administration	4 weeks after administration		Yes	2021-05-06	0	0	–	–	–	–	–	–	–	–
							3 months after administration	3 months after administration		Yes	2021-06-30	0	0	0	–	–	–	–	–	–	–
Cystic lesion	Keio-02	Y	Male	39	15 years and older	–	Before administration	screening	VAS	Yes	2021-11-04	51	–	82	–	–	–	–	–	–	–
							1 day before administration	baseline		Yes	2021-11-16	77	–	100	–	–	–	–	–	–	–
							1 day after administration	1 day after administration		Yes	2021-11-18	86	-9	–	–	–	–	–	–	–	–
							3 days after administration	3 days after administration		Yes	2021-11-19	75	2	–	–	–	–	–	–	–	–
							2 weeks after administration	2 weeks after administration		Yes	2021-12-02	76	1	–	–	–	–	–	–	–	–
							4 weeks after administration	4 weeks after administration		Yes	2021-12-16	87	-10	–	–	–	–	–	–	–	–
							3 months after administration	3 months after administration		Yes	2022-02-03	100	-23	100	0	–	–	–	–	–	–
Cystic lesion	Keio-03	Y	Female	16	15 years and older	–	Before administration	screening	VAS	Yes	2021-12-09	64	–	66	–	–	–	–	–	–	–
							1 day before administration	baseline		Yes	2021-12-21	62	–	65	–	–	–	–	–	–	–
							1 day after administration	1 day after administration		Yes	2021-12-23	100	-38	–	–	–	–	–	–	–	–
							3 days after administration	3 days after administration		Yes	2021-12-24	86	-24	–	–	–	–	–	–	–	–
							2 weeks after administration	2 weeks after administration		Yes	2022-01-05	90	-28	–	–	–	–	–	–	–	–
							4 weeks after administration	4 weeks after administration		Yes	2022-01-19	67	-5	–	–	–	–	–	–	–	–
							3 months after administration	3 months after administration		Yes	2022-03-23	30	32	26	39	–	–	–	–	–	–
Cystic lesion	Keio-04	Y	Male	22	15 years and older	–	Before administration	screening	VAS	Yes	2022-12-15	20	–	64	–	–	–	–	–	–	–
							1 day before administration	baseline		Yes	2023-01-10	29	–	71	–	–	–	–	–	–	–
							1 day after administration	1 day after administration		Yes	2023-01-12	31	-2	–	–	–	–	–	–	–	–
							3 days after administration	3 days after administration		Yes	2023-01-13	45	-16	–	–	–	–	–	–	–	–
							2 weeks after administration	2 weeks after administration		Yes	2023-01-23	18	11	–	–	–	–	–	–	–	–
							4 weeks after administration	4 weeks after administration		Yes	2023-02-14	21	8	–	–	–	–	–	–	–	–
							3 months after administration	3 months after administration		Yes	2023-03-30	16	13	18	53	–	–	–	–	–	–
Diffuse lesion	Kyorin-04	Y	Male	24	15 years and older	–	Before administration	screening	VAS	Yes	2021-11-05	36	–	68	–	–	–	–	–	–	–
							1 day before administration	baseline		Yes	2021-11-24	19	–	73	–	–	–	–	–	–	–
							1 day after administration	1 day after administration		Yes	2021-11-26	86	-67	–	–	–	–	–	–	–	–
							3 days after administration	3 days after administration		Yes	2021-11-27	63	-44	–	–	–	–	–	–	–	–
							2 weeks after administration	2 weeks after administration		Yes	2021-12-10	21	-2	–	–	–	–	–	–	–	–
							4 weeks after administration	4 weeks after administration		Yes	2021-12-24	8	11	–	–	–	–	–	–	–	–
							3 months after administration	3 months after administration		Yes	2022-02-18	18	1	29	44	–	–	–	–	–	–
Diffuse lesion	Kyorin-05	Y	Female	22	15 years and older	–	Before administration	screening	VAS	Yes	2022-03-04	55	–	66	–	–	–	–	–	–	–
							1 day before administration	baseline		Yes	2022-03-16	11	–	58	–	–	–	–	–	–	–
							1 day after administration	1 day after administration		Yes	2022-03-18	92	-81	–	–	–	–	–	–	–	–
							3 days after administration	3 days after administration		Yes	2022-03-19	20	-9	–	–	–	–	–	–	–	–
							2 weeks after administration	2 weeks after administration		Yes	2022-04-01	68	-57	–	–	–	–	–	–	–	–
							4 weeks after administration	4 weeks after administration		Yes	2022-04-15	58	-47	–	–	–	–	–	–	–	–
							3 months after administration	3 months after administration		Yes	2022-06-10	45	-34	42	16	–	–	–	–	–	–

Pain scores associated with target lesion
Analysis object: FAS

											The patient him/herself					guardian					
												Maximum pain in 24 hours		Maximum pain for 1 week				Maximum pain in 24 hours		Maximum pain for 1 week	
Lesion	Case number	PPS	Gender	Age (years)	Subgroup	Guardian	Point in time	Analysis point in time	Rating Scale	Implementation Yes or	Date of reply	Measured value	(−1)x Amount of change	Measured value	(−1)x Amount of change	Implementation Yes or No	Date of reply	Measured value	(−1)x Amount of change	Measured value	(−1)x Amount of change
Diffuse lesion	Kyorin−06	Y	Male	14	12 to 14 years old	mother	Before administration	screening	VAS	Yes	2022−03−04	41	−	86	−	Yes	2022−03−04	34	−	97	−
							1 day before administration	baseline		Yes	2022−03−28	5	−	75	−	Yes	2022−03−28	5	−	79	−
							1 day after administration	1 day after administration		Yes	2022−03−30	100	−95	−	−	Yes	2022−03−30	100	−95	−	−
							3 days after administration	3 days after administration		Yes	2022−03−31	56	−51	−	−	Yes	2022−03−31	56	−51	−	−
							2 weeks after administration	2 weeks after administration		Yes	2022−04−15	28	−23	−	−	Yes	2022−04−15	39	−34	−	−
							4 weeks after administration	4 weeks after administration		Yes	2022−04−28	7	−2	−	−	Yes	2022−04−28	8	−3	−	−
							3 months after administration	3 months after administration		Yes	2022−06−24	0	5	0	75	Yes	2022−06−24	0	5	0	79
Diffuse lesion	Kyorin−07	Y	Female	6	6 to 11 years old	father	Before administration	screening	VAS	Yes	2022−07−01	18	−	16	−	Yes	2022−07−01	20	−	14	−
							1 day before administration	baseline		Yes	2022−07−25	26	−	26	−	Yes	2022−07−25	9	−	9	−
							1 day after administration	1 day after administration		Yes	2022−07−27	73	−47	−	−	Yes	2022−07−27	96	−87	−	−
							3 days after administration	3 days after administration		Yes	2022−07−28	18	8	−	−	Yes	2022−07−28	31	−22	−	−
							2 weeks after administration	2 weeks after administration		Yes	2022−08−12	2	24	−	−	Yes	2022−08−12	2	7	−	−
							4 weeks after administration	4 weeks after administration		Yes	2022−09−02	4	22	−	−	Yes	2022−09−02	1	8	−	−
							3 months after administration	3 months after administration		Yes	2022−10−21	3	23	3	23	Yes	2022−10−21	0	9	0	9
Diffuse lesion	Kyorin−08	Y	Female	26	15 years and older	−	Before administration	screening	VAS	Yes	2022−10−14	69	−	85	−	−	−	−	−	−	−
							1 day before administration	baseline		Yes	2022−10−26	85	−	86	−	−	−	−	−	−	−
							1 day after administration	1 day after administration		Yes	2022−10−28	54	31	−	−	−	−	−	−	−	−
							3 days after administration	3 days after administration		Yes	2022−10−29	23	62	−	−	−	−	−	−	−	−
							2 weeks after administration	2 weeks after administration		Yes	2022−11−09	14	71	−	−	−	−	−	−	−	−
							4 weeks after administration	4 weeks after administration		Yes	2022−11−25	4	81	−	−	−	−	−	−	−	−
							3 months after administration	3 months after administration		Yes	2023−01−20	1	84	1	85	−	−	−	−	−	−
Diffuse lesion	Kobe−02	Y	Female	16	15 years and older	−	Before administration	screening	VAS	Yes	2021−04−20	31	−	−	−	−	−	−	−	−	−
							1 day before administration	baseline		Yes	2021−05−11	13	−	57	−	−	−	−	−	−	−
							1 day after administration	1 day after administration		Yes	2021−05−13	76	−63	−	−	−	−	−	−	−	−
							3 days after administration	3 days after administration		Yes	2021−05−14	42	−29	−	−	−	−	−	−	−	−
							2 weeks after administration	2 weeks after administration		Yes	2021−05−31	65	−52	−	−	−	−	−	−	−	−
							4 weeks after administration	4 weeks after administration		Yes	2021−06−14	39	−26	−	−	−	−	−	−	−	−
							3 months after administration	3 months after administration		Yes	2021−08−02	77	−64	86	−29	−	−	−	−	−	−
Diffuse lesion	Kobe−04	Y	Male	32	15 years and older	−	Before administration	screening	VAS	Yes	2021−07−26	47	−	45	−	−	−	−	−	−	−
							1 day before administration	baseline		Yes	2021−08−17	48	−	61	−	−	−	−	−	−	−
							1 day after administration	1 day after administration		Yes	2021−08−19	100	−52	−	−	−	−	−	−	−	−
							3 days after administration	3 days after administration		Yes	2021−08−20	83	−35	−	−	−	−	−	−	−	−
							2 weeks after administration	2 weeks after administration		Yes	2021−08−31	16	32	−	−	−	−	−	−	−	−
							4 weeks after administration	4 weeks after administration		Yes	2021−09−14	4	44	−	−	−	−	−	−	−	−
							3 months after administration	3 months after administration		Yes	2021−11−08	26	22	24	37	−	−	−	−	−	−
Diffuse lesion	Kobe−05	Y	Female	56	15 years and older	−	Before administration	screening	VAS	Yes	2021−09−21	27	−	50	−	−	−	−	−	−	−
							1 day before administration	baseline		Yes	2021−10−12	51	−	51	−	−	−	−	−	−	−
							1 day after administration	1 day after administration		Yes	2021−10−14	100	−49	−	−	−	−	−	−	−	−
							3 days after administration	3 days after administration		Yes	2021−10−15	71	−20	−	−	−	−	−	−	−	−
							2 weeks after administration	2 weeks after administration		Yes	2021−10−26	24	27	−	−	−	−	−	−	−	−
							4 weeks after administration	4 weeks after administration		Yes	2021−11−09	11	40	−	−	−	−	−	−	−	−
							3 months after administration	3 months after administration		Yes	2022−01−11	17	34	46	5	−	−	−	−	−	−

Pain scores associated with target lesion
Analysis object: FAS

										The patient him/herself						guardian					
												Maximum pain in 24 hours		Maximum pain for 1 week				Maximum pain in 24 hours		Maximum pain for 1 week	
Lesion	Case number	PPS	Gender	Age (years)	Subgroup	Guardian	Point in time	Analysis point in time	Rating Scale	Implementation Yes or	Date of reply	Measured value	(−1)x Amount of change	Measured value	(−1)x Amount of change	Implementation Yes or No	Date of reply	Measured value	(−1)x Amount of change	Measured value	(−1)x Amount of change
Diffuse lesion	Kobe−06	Y	Female	53	15 years and older	−	Before administration	screening	VAS	Yes	2021−12−21	18	−	22	−	−	−	−	−	−	−
							1 day before administration	baseline		Yes	2022−01−18	20	−	34	−	−	−	−	−	−	−
							1 day after administration	1 day after administration		Yes	2022−01−20	78	−58	−	−	−	−	−	−	−	−
							3 days after administration	3 days after administration		Yes	2022−01−21	23	−3	−	−	−	−	−	−	−	−
							2 weeks after administration	2 weeks after administration		Yes	2022−02−01	11	9	−	−	−	−	−	−	−	−
							4 weeks after administration	4 weeks after administration		Yes	2022−02−21	3	17	−	−	−	−	−	−	−	−
							3 months after administration	3 months after administration		Yes	2022−04−12	0	20	0	34	−	−	−	−	−	−
Diffuse lesion	Kobe−08	Y	Female	12	12 to 14 years old	mother	Before administration	screening	VAS	Yes	2022−07−11	3	−	6	−	Yes	2022−07−11	2	−	2	−
							1 day before administration	baseline		Yes	2022−07−26	4	−	5	−	Yes	2022−07−26	8	−	6	−
							1 day after administration	1 day after administration		Yes	2022−07−28	84	−80	−	−	No	−	−	−	−	−
							3 days after administration	3 days after administration		Yes	2022−07−29	85	−81	−	−	Yes	2022−07−29	90	−82	−	−
							2 weeks after administration	2 weeks after administration		Yes	2022−08−08	9	−5	−	−	Yes	2022−08−08	51	−43	−	−
							4 weeks after administration	4 weeks after administration		Yes	2022−08−22	3	1	−	−	Yes	2022−08−22	0	8	−	−
							3 months after administration	3 months after administration		Yes	2022−10−25	3	1	2	3	Yes	2022−10−25	0	8	1	5
Diffuse lesion	Kobe−10	Y	Male	13	12 to 14 years old	mother	Before administration	screening	VAS	Yes	2022−08−25	1	−	6	−	Yes	2022−08−25	4	−	9	−
							1 day before administration	baseline		Yes	2022−09−06	2	−	28	−	Yes	2022−09−06	1	−	38	−
							1 day after administration	1 day after administration		Yes	2022−09−08	93	−91	−	−	No	−	−	−	−	−
							3 days after administration	3 days after administration		Yes	2022−09−09	32	−30	−	−	Yes	2022−09−09	45	−44	−	−
							2 weeks after administration	2 weeks after administration		Yes	2022−09−20	25	−23	−	−	Yes	2022−09−20	7	−6	−	−
							4 weeks after administration	4 weeks after administration		Yes	2022−10−04	1	1	−	−	Yes	2022−10−04	0	1	−	−
							3 months after administration	3 months after administration		Yes	2022−12−06	0	2	0	28	Yes	2022−12−06	0	1	0	38
Diffuse lesion	Seiiku−01	Y	Male	5	Under 5 years old	mother	Before administration	screening	Face scale	Yes	2021−12−20	1	−	1	−	Yes	2021−12−20	1	−	1	−
							1 day before administration	baseline		Yes	2022−01−17	1	−	1	−	Yes	2022−01−17	1	−	1	−
							1 day after administration	1 day after administration		Yes	2022−01−19	4	−3	−	−	Yes	2022−01−19	2	−1	−	−
							3 days after administration	3 days after administration		Yes	2022−01−20	4	−3	−	−	Yes	2022−01−20	2	−1	−	−
							2 weeks after administration	2 weeks after administration		Yes	2022−02−02	1	0	−	−	Yes	2022−02−02	2	−1	−	−
							4 weeks after administration	4 weeks after administration		Yes	2022−02−16	1	0	−	−	Yes	2022−02−16	1	0	−	−
							3 months after administration	3 months after administration		Yes	2022−04−11	1	0	1	0	Yes	2022−04−11	1	0	1	0
Diffuse lesion	Osaka−01	Y	Male	25	15 years and older	−	Before administration	screening	VAS	Yes	2021−01−25	5	−	−	−	−	−	−	−	−	−
							1 day before administration	baseline		Yes	2021−02−01	9	−	−	−	−	−	−	−	−	−
							1 day after administration	1 day after administration		Yes	2021−02−03	6	3	−	−	−	−	−	−	−	−
							3 days after administration	3 days after administration		Yes	2021−02−05	5	4	−	−	−	−	−	−	−	−
							2 weeks after administration	2 weeks after administration		Yes	2021−02−17	76	−67	−	−	−	−	−	−	−	−
							4 weeks after administration	4 weeks after administration		Yes	2021−03−03	39	−30	−	−	−	−	−	−	−	−
							3 months after administration	3 months after administration		Yes	2021−04−28	25	−16	−	−	−	−	−	−	−	−
Diffuse lesion	Osaka−02	Y	Male	6	6 to 11 years old	mother	Before administration	screening	VAS	Yes	2021−06−30	25	−	65	−	Yes	2021−06−30	31	−	63	−
							1 day before administration	baseline		Yes	2021−07−26	8	−	26	−	Yes	2021−07−26	14	−	36	−
							1 day after administration	1 day after administration		Yes	2021−07−28	36	−28	−	−	Yes	2021−07−28	49	−35	−	−
							3 days after administration	3 days after administration		Yes	2021−07−30	68	−60	−	−	Yes	2021−07−30	76	−62	−	−
							2 weeks after administration	2 weeks after administration		Yes	2021−08−11	4	4	−	−	Yes	2021−08−11	30	−16	−	−
							4 weeks after administration	4 weeks after administration		Yes	2021−08−25	1	7	−	−	Yes	2021−08−25	15	−1	−	−
							3 months after administration	3 months after administration		Yes	2021−10−20	2	6	1	25	Yes	2021−10−20	19	−5	36	0

Pain scores associated with target lesion
Analysis object: FAS

											The patient him/herself						guardian				
												Maximum pain in 24 hours		Maximum pain for 1 week				Maximum pain in 24 hours		Maximum pain for 1 week	
Lesion	Case number	PPS	Gender	Age (years)	Subgroup	Guardian	Point in time	Analysis point in time	Rating Scale	Implementation Yes or	Date of reply	Measured value	(−1)x Amount of change	Measured value	(−1)x Amount of change	Implementation Yes or No	Date of reply	Measured value	(−1)x Amount of change	Measured value	(−1)x Amount of change
Diffuse lesion	Osaka−04	Y	Male	25	15 years and older	−	Before administration	screening	VAS	Yes	2021−10−20	60	−	85	−	−	−	−	−	−	−
							1 day before administration	baseline		Yes	2021−11−15	82	−	81	−	−	−	−	−	−	−
							1 day after administration	1 day after administration		Yes	2021−11−17	61	21	−	−	−	−	−	−	−	−
							3 days after administration	3 days after administration		Yes	2021−11−19	45	37	−	−	−	−	−	−	−	−
							2 weeks after administration	2 weeks after administration		Yes	2021−12−01	64	18	−	−	−	−	−	−	−	−
							4 weeks after administration	4 weeks after administration		Yes	2021−12−15	17	65	−	−	−	−	−	−	−	−
							3 months after administration	3 months after administration		Yes	2022−02−09	8	74	13	68	−	−	−	−	−	−
Diffuse lesion	Osaka−05	Y	Female	17	15 years and older	−	Before administration	screening	VAS	Yes	2021−11−24	26	−	48	−	−	−	−	−	−	−
							1 day before administration	baseline		Yes	2021−12−20	47	−	53	−	−	−	−	−	−	−
							1 day after administration	1 day after administration		Yes	2021−12−22	97	−50	−	−	−	−	−	−	−	−
							3 days after administration	3 days after administration		Yes	2021−12−24	41	6	−	−	−	−	−	−	−	−
							2 weeks after administration	2 weeks after administration		Yes	2022−01−05	11	36	−	−	−	−	−	−	−	−
							4 weeks after administration	4 weeks after administration		Yes	2022−01−19	8	39	−	−	−	−	−	−	−	−
							3 months after administration	3 months after administration		Yes	2022−03−16	8	39	19	34	−	−	−	−	−	−
Diffuse lesion	Tokyo−02	Y	Female	16	15 years and older	−	Before administration	screening	VAS	Yes	2021−07−15	55	−	85	−	−	−	−	−	−	−
							1 day before administration	baseline		Yes	2021−07−27	41	−	71	−	−	−	−	−	−	−
							1 day after administration	1 day after administration		Yes	2021−07−29	100	−59	−	−	−	−	−	−	−	−
							3 days after administration	3 days after administration		Yes	2021−07−30	59	−18	−	−	−	−	−	−	−	−
							2 weeks after administration	2 weeks after administration		Yes	2021−08−12	62	−21	−	−	−	−	−	−	−	−
							4 weeks after administration	4 weeks after administration		Yes	2021−08−26	45	−4	−	−	−	−	−	−	−	−
							3 months after administration	3 months after administration		Yes	2021−10−14	28	13	42	29	−	−	−	−	−	−
Diffuse lesion	Tokyo−03	Y	Female	59	15 years and older	−	Before administration	screening	VAS	Yes	2021−12−02	37	−	37	−	−	−	−	−	−	−
							1 day before administration	baseline		Yes	2021−12−21	59	−	64	−	−	−	−	−	−	−
							1 day after administration	1 day after administration		Yes	2021−12−23	97	−38	−	−	−	−	−	−	−	−
							3 days after administration	3 days after administration		Yes	2021−12−24	87	−28	−	−	−	−	−	−	−	−
							2 weeks after administration	2 weeks after administration		Yes	2022−01−06	75	−16	−	−	−	−	−	−	−	−
							4 weeks after administration	4 weeks after administration		Yes	2022−01−20	74	−15	−	−	−	−	−	−	−	−
							3 months after administration	3 months after administration		Yes	2022−03−17	24	35	34	30	−	−	−	−	−	−
Diffuse lesion	Shinshu−01	Y	Female	14	12 to 14 years old	mother	Before administration	screening	VAS	Yes	2021−07−27	64	−	65	−	Yes	2021−07−27	50	−	60	−
							1 day before administration	baseline		Yes	2021−08−10	61	−	61	−	Yes	2021−08−10	59	−	64	−
							1 day after administration	1 day after administration		Yes	2021−08−12	65	−4	−	−	−	−	−	−	−	−
							3 days after administration	3 days after administration		Yes	2021−08−13	63	−2	−	−	−	−	−	−	−	−
							2 weeks after administration	2 weeks after administration		Yes	2021−08−24	47	14	−	−	−	−	−	−	−	−
							4 weeks after administration	4 weeks after administration		Yes	2021−09−07	21	40	−	−	−	−	−	−	−	−
							3 months after administration	3 months after administration		Yes	2021−11−09	30	31	30	31	No	−	−	−	−	−
Diffuse lesion	Shinshu−03	Y	Female	52	15 years and older	−	Before administration	screening	VAS	Yes	2022−02−08	25	−	40	−	−	−	−	−	−	−
							1 day before administration	baseline		Yes	2022−02−15	18	−	38	−	−	−	−	−	−	−
							1 day after administration	1 day after administration		Yes	2022−02−17	63	−45	−	−	−	−	−	−	−	−
							3 days after administration	3 days after administration		Yes	2022−02−18	57	−39	−	−	−	−	−	−	−	−
							2 weeks after administration	2 weeks after administration		Yes	2022−03−01	42	−24	−	−	−	−	−	−	−	−
							4 weeks after administration	4 weeks after administration		Yes	2022−03−15	49	−31	−	−	−	−	−	−	−	−
							3 months after administration	3 months after administration		Yes	2022−05−13	11	7	20	18	−	−	−	−	−	−

Pain scores associated with target lesion
Analysis object: FAS

										The patient him/herself						guardian					
												Maximum pain in 24 hours		Maximum pain for 1 week				Maximum pain in 24 hours		Maximum pain for 1 week	
Lesion	Case number	PPS	Gender	Age (years)	Subgroup	Guardian	Point in time	Analysis point in time	Rating Scale	Implementation Yes or	Date of reply	Measured value	(−1)x Amount of change	Measured value	(−1)x Amount of change	Implementation Yes or No	Date of reply	Measured value	(−1)x Amount of change	Measured value	(−1)x Amount of change
Diffuse lesion	Shinshu−04	Y	Male	10	6 to 11 years old	father	Before administration	screening	VAS	Yes	2022−08−09	2	−	21	−	Yes	2022−08−09	0	−	22	−
							1 day before administration	baseline		Yes	2022−08−23	0	−	0	−	Yes	2022−08−23	0	−	0	−
							1 day after administration	1 day after administration		Yes	2022−08−25	47	−47	−	−	No	−	−	−	−	−
							3 days after administration	3 days after administration		Yes	2022−08−26	35	−35	−	−	No	−	−	−	−	−
							2 weeks after administration	2 weeks after administration		Yes	2022−09−06	22	−22	−	−	Yes	2022−09−06	42	−42	−	−
							4 weeks after administration	4 weeks after administration		Yes	2022−09−20	13	−13	−	−	Yes	2022−09−20	23	−23	−	−
							3 months after administration	3 months after administration		Yes	2022−11−15	5	−5	14	−14	Yes	2022−11−15	3	−3	3	−3
Diffuse lesion	Shinshu−05	Y	Female	10	6 to 11 years old	mother	Before administration	screening	VAS	Yes	2022−11−15	0	−	4	−	Yes	2022−11−15	0	−	0	−
							1 day before administration	baseline		Yes	2022−12−06	0	−	0	−	Yes	2022−12−06	0	−	0	−
							1 day after administration	1 day after administration		Yes	2022−12−08	89	−89	−	−	Yes	2022−12−08	100	−100	−	−
							3 days after administration	3 days after administration		Yes	2022−12−09	71	−71	−	−	Yes	2022−12−09	83	−83	−	−
							2 weeks after administration	2 weeks after administration		Yes	2022−12−20	3	−3	−	−	Yes	2022−12−20	5	−5	−	−
							4 weeks after administration	4 weeks after administration		Yes	2023−01−17	2	−2	−	−	Yes	2023−01−17	14	−14	−	−
							3 months after administration	3 months after administration		Yes	2023−02−28	0	0	0	0	Yes	2023−02−28	0	0	8	−8
