# Supplementary material for: Effect and safety of ethanolamine oleate in sclerotherapy in patients with difficult-to-resect venous malformations: A multicenter, single-arm study
Source: PLoS One. 2025 Jan 31;20(1):e0303130. doi: 10.1371/journal.pone.0303130 (PMC11785324; doi:10.1371/journal.pone.0303130)
Supplement: S4 Table — (PDF) [file pone.0303130.s007.pdf]

All adverse events
Analysis Subject: SAS
*1 1: Hemoglobinuria, 2: Blistering, 3: Swelling, 4: Other
*2 Number of days from the date of the first administration

Lesion	Case number	Gender	Age (years)	Presence or absence of onset	No.	Classification *1	Condition listed by Physician	System Organ Class	Preferred Term	Significant adverse events	Date of onset	Date of confirmation/ disappearance date/death	Number of days*2	Duration	Outcome	Severity	Seriousness
Cystic lesion	Kyorin-01	Male	6	having	1	1	Hemoglobinuria	Renal and urinary tract disorders	Hemoglobinuria	-	2021-02-09	2021-02-10	1 day	2 days	Recovery	Mild	Non-serious
					2	3	Swelling	General and systemic disorders and conditions at the site of administration	Swelling	-	2021-02-09	2021-03-12	1 day	32 days	Recovery	Mild	Non-serious
					3	4	Pain	General and systemic disorders and conditions at the site of administration	Pain	-	2021-02-09	2021-02-19	1 day	11 days	Recovery	Mild	Non-serious
					4	4	Rubefaction (reddening of the skin)	Skin and subcutaneous tissue disorders	Erythema	-	2021-02-09	2021-02-19	1 day	11 days	Recovery	Mild	Non-serious
					5	4	Vomiting	Gastrointestinal disorder	Vomiting	-	2021-02-09	2021-02-09	1 day	1 day	Recovery	Mild	Non-serious
					6	4	CK high	Clinical examination	Creatine phosphokinase increased	-	2021-02-10	2021-02-19	2 days	10 days	Recovery	Mild	Non-serious
					7	4	D-dimer high	Clinical examination	Fibrin D-dimer increased	-	2021-02-10	2021-02-19	2 days	10 days	Recovery	Moderate	Non-serious
					8	4	Amylase high	Clinical examination	Amylase increased	-	2021-02-10	2021-02-19	2 days	10 days	Recovery	Mild	Non-serious
					9	4	Positive urine ketone	Clinical examination	Positive urinary ketone	-	2021-02-10	2021-02-11	2 days	2 days	Recovery	Mild	Non-serious
					10	4	High FDP	Clinical examination	Fibrin degradation products increased	-	2021-02-10	2021-02-19	2 days	10 days	Recovery	Mild	Non-serious
Cystic lesion	Kyorin-02	Female	31	having	1	3	Swelling	General and systemic disorders and conditions at the site of administration	Swelling	-	2021-03-26	2021-04-09	2 days	15 days	Recovery	Mild	Non-serious
					2	4	Pain	General and systemic disorders and conditions at the site of administration	Pain	-	2021-03-26	2021-04-22	2 days	28 days	Recovery	Mild	Non-serious
					3	4	Rubefaction (reddening of the skin)	Skin and subcutaneous tissue disorders	Erythema	-	2021-03-26	2021-04-09	2 days	15 days	Recovery	Mild	Non-serious
					4	4	CK high	Clinical examination	Creatine phosphokinase increased	-	2021-03-26	2021-04-09	2 days	15 days	Recovery	Mild	Non-serious
					5	4	CRP increased	Clinical examination	C-reactive protein increased	-	2021-03-27	2021-04-09	3 days	14 days	Recovery	Mild	Non-serious
					6	4	Positive urine ketone	Clinical examination	Positive urinary ketone	-	2021-03-26	2021-03-27	2 days	2 days	Recovery	Mild	Non-serious

All adverse events
Analysis Subject: SAS
*1 1: Hemoglobinuria, 2: Blistering, 3: Swelling, 4: Other
*2 Number of days from the date of the first administration

MedDRA/J Ver. 26.0

Lesion	Case number	Gender	Age (years)	Presence or absence of onset	No.	Classification *1	Condition listed by Physician	System Organ Class	Preferred Term	Investigational drug treatment	Other treatment	Causal relationship	Reasons for no causal relationship	Other details	Comment
Cystic lesion	Kyorin-01	Male	6	having	1	1	Hemoglobinuria	Renal and urinary tract disorders	Hemoglobinuria	Not applicable	Treatment implementation	Causal relationship exists	-	-	-
					2	3	Swelling	General and systemic disorders and conditions at the site of administration	Swelling	Not applicable	No treatment	Causal relationship exists	-	-	-
					3	4	Pain	General and systemic disorders and conditions at the site of administration	Pain	Not applicable	Treatment implementation	Causal relationship exists	-	-	-
					4	4	Rubefaction (reddening of the skin)	Skin and subcutaneous tissue disorders	Erythema	Not applicable	No treatment	Causal relationship exists	-	-	-
					5	4	Vomiting	Gastrointestinal disorder	Vomiting	Not applicable	No treatment	No causal relationship	Due to anesthesia for sclerotherapy	-	-
					6	4	CK high	Clinical examination	Creatine phosphokinase increased	Not applicable	No treatment	Causal relationship exists	-	-	-
					7	4	D-dimer high	Clinical examination	Fibrin D-dimer increased	Not applicable	No treatment	Causal relationship exists	-	-	-
					8	4	Amylase high	Clinical examination	Amylase increased	Not applicable	No treatment	Causal relationship exists	-	-	-
					9	4	Positive urine ketone	Clinical examination	Positive urinary ketone	Not applicable	No treatment	No causal relationship	Other	Postoperative Vomiting and fasting effects	-
					10	4	High FDP	Clinical examination	Fibrin degradation products increased	Not applicable	No treatment	Causal relationship exists	-	-	-
Cystic lesion	Kyorin-02	Female	31	having	1	3	Swelling	General and systemic disorders and conditions at the site of administration	Swelling	Not applicable	No treatment	Causal relationship exists	-	-	-
					2	4	Pain	General and systemic disorders and conditions at the site of administration	Pain	Not applicable	Treatment implementation	Causal relationship exists	-	-	-
					3	4	Rubefaction (reddening of the skin)	Skin and subcutaneous tissue disorders	Erythema	Not applicable	No treatment	Causal relationship exists	-	-	-
					4	4	CK high	Clinical examination	Creatine phosphokinase increased	Not applicable	No treatment	Causal relationship exists	-	-	-
					5	4	CRP increased	Clinical examination	C-reactive protein increased	Not applicable	No treatment	Causal relationship exists	-	-	-
					6	4	Positive urine ketone	Clinical examination	Positive urinary ketone	Not applicable	No treatment	No causal relationship	Other	Due to starvation from fasting following surgery	-

All adverse events
Analysis Subject: SAS
*1 1: Hemoglobinuria, 2: Blistering, 3: Swelling, 4: Other
*2 Number of days from the date of the first administration

Lesion	Case number	Gender	Age (years)	Presence or absence of onset	No.	Classification *1	Condition listed by Physician	System Organ Class	Preferred Term	Significant adverse events	Date of onset	Date of confirmation/ disappearance date/death	Number of days*2	Duration	Outcome	Severity	Seriousness
Cystic lesion	Kyorin-03	Male	17	having	1	4	Pain	General and systemic disorders and conditions at the site of administration	Pain	–	2021-07-20	2021-08-06	1 day	18 days	Recovery	Mild	Non-serious
					2	4	Serum bilirubin increased	Clinical examination	Serum bilirubin increased	–	2021-07-21	2021-08-06	2 days	17 days	Recovery	Mild	Non-serious
					3	4	Fever	General and systemic disorders and conditions at the site of administration	Fever	–	2021-10-07	2021-10-08	80 days	2 days	Recovery	Mild	Non-serious
Cystic lesion	Junten-01	Female	3	having	1	4	CPK increased	Clinical examination	Creatine phosphokinase increased	–	2021-03-18	2021-04-02	2 days	16 days	Recovery	Mild	Non-serious
Cystic lesion	Junten-02	Male	14	having	1	1	Hemoglobinuria	Renal and urinary tract disorders	Hemoglobinuria	–	2021-09-30	2021-10-01	1 day	2 days	Recovery	Mild	Non-serious
Cystic lesion	Junten-03	Male	11	having	1	4	COVID-19 PCR positive	Infectious and parasitic diseases	COVID-19	–	2022-02-17	2022-02-19	1 day	3 days	Recovery	Mild	Non-serious
Cystic lesion	Junten-04	Male	8	nil	–	–	–	–	–	–	–	–	–	–	–	–	–
Cystic lesion	Junten-05	Female	5	nil	–	–	–	–	–	–	–	–	–	–	–	–	–
Cystic lesion	Kobe-01	Female	78	having	1	4	Postoperative Pain	General and systemic disorders and conditions at the site of administration	Pain	–	2021-03-03	2021-03-05	1 day	3 days	Recovery	Mild	Non-serious
					2	4	Nausea	Gastrointestinal disorder	Nausea	–	2021-03-03	2021-03-04	1 day	2 days	Recovery	Mild	Non-serious
					3	3	Swelling	General and systemic disorders and conditions at the site of administration	Swelling	–	2021-03-03	2021-03-16	1 day	14 days	Recovery	Mild	Non-serious
					4	4	Urine occult blood positive	Clinical examination	Urine occult blood positive	–	2021-02-15	2021-05-24	-16days	99 days	Unrecovered	Mild	Non-serious
Cystic lesion	Kobe-03	Male	5	having	1	4	Fever	General and systemic disorders and conditions at the site of administration	Fever	–	2021-08-12	2021-08-20	2 days	9 days	Recovery	Mild	Non-serious
					2	4	Pain	General and systemic disorders and conditions at the site of administration	Pain	–	2021-08-11	2021-08-20	1 day	10 days	Recovery	Mild	Non-serious

All adverse events
Analysis Subject: SAS
*1 1: Hemoglobinuria, 2: Blistering, 3: Swelling, 4: Other
*2 Number of days from the date of the first administration

MedDRA/J Ver. 26.0

Lesion	Case number	Gender	Age (years)	Presence or absence of onset	No.	Classification *1	Condition listed by Physician	System Organ Class	Preferred Term	Investigational drug treatment	Other treatment	Causal relationship	Reasons for no causal relationship	Other details	Comment
Cystic lesion	Kyorin-03	Male	17	having	1	4	Pain	General and systemic disorders and conditions at the site of administration	Pain	Not applicable	Treatment implementation	Causal relationship exists	-	-	-
					2	4	Serum bilirubin increased	Clinical examination	Serum bilirubin increased	Not applicable	No treatment	Causal relationship exists	-	-	-
					3	4	Fever	General and systemic disorders and conditions at the site of administration	Fever	Not applicable	Treatment implementation	No causal relationship	Due to concomittreatment implementation medications	-	-
Cystic lesion	Junten-01	Female	3	having	1	4	CPK increased	Clinical examination	Creatine phosphokinase increased	Not applicable	No treatment	Causal relationship exists	-	-	-
Cystic lesion	Junten-02	Male	14	having	1	1	Hemoglobinuria	Renal and urinary tract disorders	Hemoglobinuria	Not applicable	Treatment implementation	Causal relationship exists	-	-	-
Cystic lesion	Junten-03	Male	11	having	1	4	COVID-19 PCR positive	Infectious and parasitic diseases	COVID-19	Not applicable	No treatment	No causal relationship	Accidental	-	-
Cystic lesion	Junten-04	Male	8	nil	-	-	-	-	-	-	-	-	-	-	-
Cystic lesion	Junten-05	Female	5	nil	-	-	-	-	-	-	-	-	-	-	-
Cystic lesion	Kobe-01	Female	78	having	1	4	Postoperative Pain	General and systemic disorders and conditions at the site of administration	Pain	Not applicable	Treatment implementation	Causal relationship exists	-	-	-
					2	4	Nausea	Gastrointestinal disorder	Nausea	Not applicable	Treatment implementation	No causal relationship	Due to anesthesia for sclerotherapy	-	-
					3	3	Swelling	General and systemic disorders and conditions at the site of administration	Swelling	Not applicable	No treatment	Causal relationship exists	-	-	-
					4	4	Urine occult blood positive	Clinical examination	Urine occult blood positive	Not applicable	No treatment	No causal relationship	Other	Appeared prior to administration of study drug	No follow-up is necessary because the patient does not have abnormal laboratory values Causal relationship exists to the clinical trial and will be followed up by her family physician in the future.
Cystic lesion	Kobe-03	Male	5	having	1	4	Fever	General and systemic disorders and conditions at the site of administration	Fever	Not applicable	Treatment implementation	Causal relationship exists	-	-	-
					2	4	Pain	General and systemic disorders and conditions at the site of administration	Pain	Not applicable	Treatment implementation	Causal relationship exists	-	-	-

All adverse events
Analysis Subject: SAS
*1 1: Hemoglobinuria, 2: Blistering, 3: Swelling, 4: Other
*2 Number of days from the date of the first administration

Lesion	Case number	Gender	Age (years)	Presence or absence of onset	No.	Classification *1	Condition listed by Physician	System Organ Class	Preferred Term	Significant adverse events	Date of onset	Date of confirmation/ disappearance date/death	Number of days*2	Duration	Outcome	Severity	Seriousness
Cystic lesion	Kobe-09	Male	8	having	1	4	Pain	General and systemic disorders and conditions at the site of administration	Pain	-	2022-08-10	2022-08-15	1 day	6 days	Recovery	Mild	Non-serious
					2	4	Urine occult blood positive	Clinical examination	Urine occult blood positive	-	2022-08-11	2022-08-12	2 days	2 days	Recovery	Mild	Non-serious
					3	4	Upper respiratory tract infection	Infectious and parasitic diseases	Upper respiratory tract infection	-	2022-08-20	2022-08-21	11 days	2 days	Recovery	Mild	Non-serious
					4	4	Fever	General and systemic disorders and conditions at the site of administration	Fever	-	2022-08-10	2022-08-12	1 day	3 days	Recovery	Mild	Non-serious
Cystic lesion	Kobe-11	Male	44	having	1	4	Pain	General and systemic disorders and conditions at the site of administration	Pain	-	2023-01-25	2023-02-01	1 day	8 days	Recovery	Mild	Non-serious
Cystic lesion	Osaka-03	Female	50	having	1	3	Swelling	General and systemic disorders and conditions at the site of administration	Swelling	-	2021-10-19	2021-11-17	1 day	30 days	Recovery	Moderate	Non-serious
					2	4	Pain	General and systemic disorders and conditions at the site of administration	Pain	-	2021-10-19	2022-01-12	1 day	86 days	Recovery	Mild	Non-serious
					3	4	Constipation	Gastrointestinal disorder	Constipation	-	2021-10-25	2021-11-17	7 days	24 days	Recovery	Mild	Non-serious
Cystic lesion	Osaka-06	Male	5	having	1	1	Hemoglobinuria	Renal and urinary tract disorders	Hemoglobinuria	-	2022-05-24	2022-05-27	1 day	4 days	Recovery	Moderate	Non-serious
					2	4	Subcutaneous hemorrhage	Skin and subcutaneous tissue disorders	Subcutaneous hemorrhage	-	2022-05-25	2022-06-08	2 days	15 days	Recovery	Mild	Non-serious
					3	4	Pain	General and systemic disorders and conditions at the site of administration	Pain	-	2022-05-24	2022-05-25	1 day	2 days	Recovery	Mild	Non-serious
Cystic lesion	Tokyo-01	Female	26	having	1	1	Hemoglobinuria	Renal and urinary tract disorders	Hemoglobinuria	-	2021-03-10	2021-03-10	1 day	1 day	Recovery	Mild	Non-serious
Cystic lesion	Tokyo-04	Male	33	having	1	1	Hemoglobinuria	Renal and urinary tract disorders	Hemoglobinuria	-	2022-05-27	2022-05-27	1 day	1 day	Recovery	Mild	Non-serious
					2	4	Eczema (lower leg)	Skin and subcutaneous tissue disorders	Eczema	-	2022-05-30	2022-06-04	4 days	six days	Recovery	Mild	Non-serious
Cystic lesion	Tokyo-05	Male	11	having	1	4	Headache	Nervous System Disorders	Headache	-	2022-11-07	2022-11-13	20 days	7 days	Recovery	Mild	Non-serious
					2	4	Postoperative Pain	General and systemic disorders and conditions at the site of administration	Pain	-	2022-10-21	2022-10-30	3 days	10 days	Recovery	Mild	Non-serious

All adverse events
Analysis Subject: SAS
*1 1: Hemoglobinuria, 2: Blistering, 3: Swelling, 4: Other
*2 Number of days from the date of the first administration

MedDRA/J Ver. 26.0

Lesion	Case number	Gender	Age (years)	Presence or absence of onset	No.	Classification *1	Condition listed by Physician	System Organ Class	Preferred Term	Investigational drug treatment	Other treatment	Causal relationship	Reasons for no causal relationship	Other details	Comment
Cystic lesion	Kobe-09	Male	8	having	1	4	Pain	General and systemic disorders and conditions at the site of administration	Pain	Not applicable	Treatment implementation	Causal relationship exists	–	–	–
					2	4	Urine occult blood positive	Clinical examination	Urine occult blood positive	Not applicable	No treatment	Causal relationship exists	–	–	–
					3	4	Upper respiratory tract infection	Infectious and parasitic diseases	Upper respiratory tract infection	Not applicable	No treatment	No causal relationship	Accidental	–	–
					4	4	Fever	General and systemic disorders and conditions at the site of administration	Fever	Not applicable	No treatment	Causal relationship exists	–	–	–
Cystic lesion	Kobe-11	Male	44	having	1	4	Pain	General and systemic disorders and conditions at the site of administration	Pain	Not applicable	Treatment implementation	Causal relationship exists	–	–	–
Cystic lesion	Osaka-03	Female	50	having	1	3	Swelling	General and systemic disorders and conditions at the site of administration	Swelling	Not applicable	No treatment	Causal relationship exists	–	–	–
					2	4	Pain	General and systemic disorders and conditions at the site of administration	Pain	Not applicable	Treatment implementation	Causal relationship exists	–	–	–
					3	4	Constipation	Gastrointestinal disorder	Constipation	Not applicable	Treatment implementation	No causal relationship	Other	Depends on patient predisposition	–
Cystic lesion	Osaka-06	Male	5	having	1	1	Hemoglobinuria	Renal and urinary tract disorders	Hemoglobinuria	Not applicable	Treatment implementation	Causal relationship exists	–	–	–
					2	4	Subcutaneous hemorrhage	Skin and subcutaneous tissue disorders	Subcutaneous hemorrhage	Not applicable	No treatment	Causal relationship exists	–	–	–
					3	4	Pain	General and systemic disorders and conditions at the site of administration	Pain	Not applicable	No treatment	Causal relationship exists	–	–	–
Cystic lesion	Tokyo-01	Female	26	having	1	1	Hemoglobinuria	Renal and urinary tract disorders	Hemoglobinuria	Not applicable	Treatment implementation	Causal relationship exists	–	–	–
Cystic lesion	Tokyo-04	Male	33	having	1	1	Hemoglobinuria	Renal and urinary tract disorders	Hemoglobinuria	Not applicable	Treatment implementation	Causal relationship exists	–	–	–
					2	4	Eczema (lower leg)	Skin and subcutaneous tissue disorders	Eczema	Not applicable	No treatment	No causal relationship	Accidental	–	–
Cystic lesion	Tokyo-05	Male	11	having	1	4	Headache	Nervous System Disorders	Headache	Not applicable	Treatment implementation	No causal relationship	Accidental	–	–
					2	4	Postoperative Pain	General and systemic disorders and conditions at the site of administration	Pain	Not applicable	Treatment implementation	Causal relationship exists	–	–	–

All adverse events
Analysis Subject: SAS
*1 1: Hemoglobinuria, 2: Blistering, 3: Swelling, 4: Other
*2 Number of days from the date of the first administration

Lesion	Case number	Gender	Age (years)	Presence or absence of onset	No.	Classification *1	Condition listed by Physician	System Organ Class	Preferred Term	Significant adverse events	Date of onset	Date of confirmation/ disappearance date/death	Number of days*2	Duration	Outcome	Severity	Seriousness
Cystic lesion	Shinshu-02	Female	37	having	1	1	Hemoglobinuria	Renal and urinary tract disorders	Hemoglobinuria	-	2021-08-18	2021-09-14	1 day	28 days	Recovery	Mild	Non-serious
					2	4	Postoperative Pain	General and systemic disorders and conditions at the site of administration	Pain	-	2021-08-18	2021-09-14	1 day	28 days	Recovery	Severe	Non-serious
					3	3	Swelling	General and systemic disorders and conditions at the site of administration	Swelling	-	2021-08-19	2021-08-31	2 days	13 days	Recovery	Mild	Non-serious
					4	4	Subcutaneous hemorrhage	Skin and subcutaneous tissue disorders	Subcutaneous hemorrhage	-	2021-08-19	2021-08-31	2 days	13 days	Recovery	Mild	Non-serious
Cystic lesion	Keio-01	Female	27	having	1	1	Hemoglobinuria	Renal and urinary tract disorders	Hemoglobinuria	-	2021-04-08	2021-04-09	1 day	2 days	Recovery	Mild	Non-serious
Cystic lesion	Keio-02	Male	39	having	1	1	Hemoglobinuria	Renal and urinary tract disorders	Hemoglobinuria	-	2021-11-17	2021-11-17	1 day	1 day	Recovery	Mild	Non-serious
					2	4	Numbness	Nervous System Disorders	Sensory paralysis	-	2021-11-17	2021-11-17	1 day	1 day	Recovery	Mild	Non-serious
Cystic lesion	Keio-03	Female	16	having	1	1	Hemoglobinuria	Renal and urinary tract disorders	Hemoglobinuria	-	2021-12-22	2022-01-05	1 day	15 days	Recovery	Mild	Non-serious
					2	4	Pain	General and systemic disorders and conditions at the site of administration	Pain	-	2021-12-22	2022-03-23	1 day	92 days	Recovery	Mild	Non-serious
Cystic lesion	Keio-04	Male	22	having	1	4	High urinary urobilinogen levels	Clinical examination	Urinary urobilinogen increased	-	2022-12-15	2023-02-14	-27days	62 days	Recovery	Mild	Non-serious
					2	4	Headache	Nervous System Disorders	Headache	-	2023-01-12	2023-01-13	2 days	2 days	Recovery	Mild	Non-serious
					3	4	Fever	General and systemic disorders and conditions at the site of administration	Fever	-	2023-01-12	2023-01-22	2 days	11 days	Recovery	Mild	Non-serious

All adverse events
Analysis Subject: SAS
*1 1: Hemoglobinuria, 2: Blistering, 3: Swelling, 4: Other
*2 Number of days from the date of the first administration

MedDRA/J Ver. 26.0

Lesion	Case number	Gender	Age (years)	Presence or absence of onset	No.	Classification *1	Condition listed by Physician	System Organ Class	Preferred Term	Investigational drug treatment	Other treatment	Causal relationship	Reasons for no causal relationship	Other details	Comment
Cystic lesion	Shinshu-02	Female	37	having	1	1	Hemoglobinuria	Renal and urinary tract disorders	Hemoglobinuria	Not applicable	Treatment implementation	Causal relationship exists	–	–	–
					2	4	Postoperative Pain	General and systemic disorders and conditions at the site of administration	Pain	Not applicable	Treatment implementation	Causal relationship exists	–	–	–
					3	3	Swelling	General and systemic disorders and conditions at the site of administration	Swelling	Not applicable	No treatment	Causal relationship exists	–	–	–
					4	4	Subcutaneous hemorrhage	Skin and subcutaneous tissue disorders	Subcutaneous hemorrhage	Not applicable	No treatment	Causal relationship exists	–	–	–
Cystic lesion	Keio-01	Female	27	having	1	1	Hemoglobinuria	Renal and urinary tract disorders	Hemoglobinuria	Not applicable	Treatment implementation	Causal relationship exists	–	–	–
Cystic lesion	Keio-02	Male	39	having	1	1	Hemoglobinuria	Renal and urinary tract disorders	Hemoglobinuria	Not applicable	Treatment implementation	Causal relationship exists	–	–	–
					2	4	Numbness	Nervous System Disorders	Sensory paralysis	Not applicable	No treatment	Causal relationship exists	–	–	–
Cystic lesion	Keio-03	Female	16	having	1	1	Hemoglobinuria	Renal and urinary tract disorders	Hemoglobinuria	Not applicable	Treatment implementation	Causal relationship exists	–	–	–
					2	4	Pain	General and systemic disorders and conditions at the site of administration	Pain	Not applicable	Treatment implementation	Causal relationship exists	–	–	–
Cystic lesion	Keio-04	Male	22	having	1	4	High urinary urobilinogen levels	Clinical examination	Urinary urobilinogen increased	Not applicable	No treatment	No causal relationship	Accidental	–	–
					2	4	Headache	Nervous System Disorders	Headache	Not applicable	Treatment implementation	Causal relationship exists	–	–	–
					3	4	Fever	General and systemic disorders and conditions at the site of administration	Fever	Not applicable	Treatment implementation	Causal relationship exists	–	–	–

All adverse events
Analysis Subject: SAS
*1 1: Hemoglobinuria, 2: Blistering, 3: Swelling, 4: Other
*2 Number of days from the date of the first administration

Lesion	Case number	Gender	Age (years)	Presence or absence of onset	No.	Classification *1	Condition listed by Physician	System Organ Class	Preferred Term	Significant adverse events	Date of onset	Date of confirmation/ disappearance date/death	Number of days*2	Duration	Outcome	Severity	Seriousness
Diffuse lesion	Kyorin-04	Male	24	having	1	1	Hemoglobinuria	Renal and urinary tract disorders	Hemoglobinuria	-	2021-11-25	2021-11-26	1 day	2 days	Recovery	Mild	Non-serious
					2	4	Pain	General and systemic disorders and conditions at the site of administration	Pain	-	2021-11-25	2021-11-27	1 day	3 days	Recovery	Mild	Non-serious
					3	4	CK high	Clinical examination	Creatine phosphokinase increased	-	2021-11-26	2021-12-10	2 days	15 days	Recovery	Moderate	Non-serious
Diffuse lesion	Kyorin-05	Female	22	having	1	1	Hemoglobinuria	Renal and urinary tract disorders	Hemoglobinuria	-	2022-03-17	2022-03-17	1 day	1 day	Recovery	Mild	Non-serious
					2	4	Drug rash	Skin and subcutaneous tissue disorders	Drug rash	-	2022-03-17	2022-03-17	1 day	1 day	Recovery	Mild	Non-serious
					3	4	Pain Exacerbation	General and systemic disorders and conditions at the site of administration	Pain	-	2022-03-17	2022-03-19	1 day	3 days	Recovery	Mild	Non-serious
					4	4	Numbness	Nervous System Disorders	Sensory paralysis	-	2022-03-17	2022-04-15	1 day	30 days	Recovery	Mild	Non-serious
Diffuse lesion	Kyorin-06	Male	14	having	1	1	Hemoglobinuria	Renal and urinary tract disorders	Hemoglobinuria	-	2022-03-29	2022-03-31	1 day	3 days	Recovery	Mild	Non-serious
					2	4	Pain	General and systemic disorders and conditions at the site of administration	Pain	-	2022-03-29	2022-06-24	1 day	88 days	Recovery	Moderate	Non-serious
					3	4	Rubefaction (reddening of the skin)	Skin and subcutaneous tissue disorders	Erythema	-	2022-03-30	2022-04-15	2 days	17 days	Recovery	Mild	Non-serious
					4	3	Swelling	General and systemic disorders and conditions at the site of administration	Swelling	-	2022-03-30	2022-04-15	2 days	17 days	Recovery	Mild	Non-serious
					5	4	Numbness	Nervous system disorders	Sensory paralysis	-	2022-03-30	2022-06-24	2 days	87 days	Light	Mild	Non-serious

All adverse events
Analysis Subject: SAS
*1 1: Hemoglobinuria, 2: Blistering, 3: Swelling, 4: Other
*2 Number of days from the date of the first administration

MedDRA/J Ver. 26.0

Lesion	Case number	Gender	Age (years)	Presence or absence of onset	No.	Classification *1	Condition listed by Physician	System Organ Class	Preferred Term	Investigational drug treatment	Other treatment	Causal relationship	Reasons for no causal relationship	Other details	Comment
Diffuse lesion	Kyorin-04	Male	24	having	1	1	Hemoglobinuria	Renal and urinary tract disorders	Hemoglobinuria	Not applicable	Treatment implementation	Causal relationship exists	–	–	–
					2	4	Pain	General and systemic disorders and conditions at the site of administration	Pain	Not applicable	Treatment implementation	Causal relationship exists	–	–	–
					3	4	CK high	Clinical examination	Creatine phosphokinase increased	Not applicable	No treatment	Causal relationship exists	–	–	–
Diffuse lesion	Kyorin-05	Female	22	having	1	1	Hemoglobinuria	Renal and urinary tract disorders	Hemoglobinuria	Not applicable	Treatment implementation	Causal relationship exists	–	–	–
					2	4	Drug rash	Skin and subcutaneous tissue disorders	Drug rash	Unchanged	No treatment	No causal relationship	Other	During preoperative administration of intravenous levofloxacin (Implementationibiotic), there was a rash on the Implementationerior thoracic region, which resolved when the drug	–
					3	4	Pain Exacerbation	General and systemic disorders and conditions at the site of administration	Pain	Not applicable	Treatment implementation	Causal relationship exists	–	–	Inflammation due to local injection of the sclerosing agent, which is Causal relationship exists.
					4	4	Numbness	Nervous System Disorders	Sensory paralysis	Not applicable	No treatment	Causal relationship exists	–	–	–
Diffuse lesion	Kyorin-06	Male	14	having	1	1	Hemoglobinuria	Renal and urinary tract disorders	Hemoglobinuria	Not applicable	Treatment implementation	Causal relationship exists	–	–	–
					2	4	Pain	General and systemic disorders and conditions at the site of administration	Pain	Not applicable	Treatment implementation	Causal relationship exists	–	–	–
					3	4	Rubefaction (reddening of the skin)	Skin and subcutaneous tissue disorders	Erythema	Not applicable	Treatment implementation	Causal relationship exists	–	–	–
					4	3	Swelling	General and systemic disorders and conditions at the site of administration	Swelling	Not applicable	Treatment implementation	Causal relationship exists	–	–	–
					5	4	Numbness	Nervous system disorders	Sensory paralysis	Not applicable	Treatment implementation	Causal relationship exists	–	–	Since the Numbness is clearly improving, it is judged that the patient will fully recover with the remaining months of oral methycobal.

All adverse events
Analysis Subject: SAS
*1 1: Hemoglobinuria, 2: Blistering, 3: Swelling, 4: Other
*2 Number of days from the date of the first administration

Lesion	Case number	Gender	Age (years)	Presence or absence of onset	No.	Classification *1	Condition listed by Physician	System Organ Class	Preferred Term	Significant adverse events	Date of onset	Date of confirmation/ disappearance date/death	Number of days*2	Duration	Outcome	Severity	Seriousness
Diffuse lesion	Kyorin-07	Female	6	having	1	4	Pain	General and systemic disorders and conditions at the site of administration	Pain	-	2022-07-26	2022-08-12	1 day	18 days	Recovery	Mild	Non-serious
					2	3	Swelling	General and systemic disorders and conditions at the site of administration	Swelling	-	2022-07-27	2022-08-12	2 days	17 days	Recovery	Mild	Non-serious
					3	4	Numbness	Nervous System Disorders	Sensory paralysis	-	2022-07-27	2022-08-12	2 days	17 days	Recovery	Mild	Non-serious
Diffuse lesion	Kyorin-08	Female	26	having	1	1	Hemoglobinuria	Renal and urinary tract disorders	Hemoglobinuria	-	2022-10-27	2022-10-28	1 day	2 days	Recovery	Mild	Non-serious
					2	4	CK high	Clinical examination	Creatine phosphokinase increased	-	2022-10-28	2022-11-25	2 days	29 days	Recovery	Mild	Non-serious
					3	4	COVID-19 PCR positive	Infectious and parasitic diseases	COVID-19	-	2022-11-26	2022-12-05	31 days	10 days	Recovery	Mild	Non-serious
Diffuse lesion	Kobe-02	Female	16	having	1	4	Pain	General and systemic disorders and conditions at the site of administration	Pain	-	2021-05-12	2021-08-02	1 day	83 days	Recovery	Mild	Non-serious
					2	4	Nausea	Gastrointestinal disorder	Nausea	-	2021-05-12	2021-05-12	1 day	1 day	Recovery	Mild	Non-serious
					3	3	Swelling	General and systemic disorders and conditions at the site of administration	Swelling	-	2021-05-13	2021-08-02	2 days	82 days	Recovery	Mild	Non-serious
					4	4	Urine occult blood positive	Clinical examination	Urine occult blood positive	-	2021-05-13	2021-05-14	2 days	2 days	Recovery	Mild	Non-serious

All adverse events
Analysis Subject: SAS
*1 1: Hemoglobinuria, 2: Blistering, 3: Swelling, 4: Other
*2 Number of days from the date of the first administration

MedDRA/J Ver. 26.0

Lesion	Case number	Gender	Age (years)	Presence or absence of onset	No.	Classification *1	Condition listed by Physician	System Organ Class	Preferred Term	Investigational drug treatment	Other treatment	Causal relationship	Reasons for no causal relationship	Other details	Comment
Diffuse lesion	Kyorin-07	Female	6	having	1	4	Pain	General and systemic disorders and conditions at the site of administration	Pain	Not applicable	Treatment implementation	Causal relationship exists	-	-	-
					2	3	Swelling	General and systemic disorders and conditions at the site of administration	Swelling	Not applicable	Treatment implementation	Causal relationship exists	-	-	Kalonal fine granules 20%.
					3	4	Numbness	Nervous System Disorders	Sensory paralysis	Not applicable	No treatment	Causal relationship exists	-	-	-
Diffuse lesion	Kyorin-08	Female	26	having	1	1	Hemoglobinuria	Renal and urinary tract disorders	Hemoglobinuria	Not applicable	Treatment implementation	Causal relationship exists	-	-	-
					2	4	CK high	Clinical examination	Creatine phosphokinase increased	Not applicable	No treatment	Causal relationship exists	-	-	-
					3	4	COVID-19 PCR positive	Infectious and parasitic diseases	COVID-19	Not applicable	Treatment implementation	No causal relationship	Accidental	-	Infectious diseases are unavoidable.
Diffuse lesion	Kobe-02	Female	16	having	1	4	Pain	General and systemic disorders and conditions at the site of administration	Pain	Not applicable	Treatment implementation	Causal relationship exists	-	-	-
					2	4	Nausea	Gastrointestinal disorder	Nausea	Not applicable	Treatment implementation	No causal relationship	Due to anesthesia for sclerotherapy	-	-
					3	3	Swelling	General and systemic disorders and conditions at the site of administration	Swelling	Not applicable	No treatment	Causal relationship exists	-	-	-
					4	4	Urine occult blood positive	Clinical examination	Urine occult blood positive	Not applicable	No treatment	Causal relationship exists	-	-	-

All adverse events
Analysis Subject: SAS
*1 1: Hemoglobinuria, 2: Blistering, 3: Swelling, 4: Other
*2 Number of days from the date of the first administration

Lesion	Case number	Gender	Age (years)	Presence or absence of onset	No.	Classification *1	Condition listed by Physician	System Organ Class	Preferred Term	Significant adverse events	Date of onset	Date of confirmation/ disappearance date/death	Number of days*2	Duration	Outcome	Severity	Seriousness
Diffuse lesion	Kobe−04	Male	32	having	1	1	Hemoglobinuria	Renal and urinary tract disorders	Hemoglobinuria	−	2021−08−18	2021−08−19	1 day	2 days	Recovery	Mild	Non−serious
					2	4	Uulnar nerve palsy	Nervous System Disorders	Uulnar nerve palsy	−	2021−08−19	2021−11−08	2 days	82 days	Light	Moderate	Non−serious
					3	4	Pain	General and systemic disorders and conditions at the site of administration	Pain	−	2021−08−18	2021−09−14	1 day	28 days	Recovery	Mild	Non−serious
					4	4	Epigastric Pain	Gastrointestinal disorder	Epigastric Pain	−	2021−08−29	2021−09−02	12 days	5 days	Recovery	Mild	Non−serious
					5	4	Lightheadedness	Nervous System Disorders	Floating dizziness	−	2021−09−01	2021−09−01	15 days	1 day	Recovery	Mild	Non−serious
Diffuse lesion	Kobe−05	Female	56	having	1	4	Pain	General and systemic disorders and conditions at the site of administration	Pain	−	2021−10−13	2021−10−26	1 day	14 days	Recovery	Mild	Non−serious
Diffuse lesion	Kobe−06	Female	53	having	1	4	Pain	General and systemic disorders and conditions at the site of administration	Pain	−	2022−01−19	2022−02−10	1 day	23 days	Recovery	Mild	Non−serious
					2	4	Urine occult blood positive	Clinical examination	Urine occult blood positive	−	2022−01−20	2022−02−01	2 days	13 days	Recovery	Mild	Non−serious
					3	4	Fever	General and systemic disorders and conditions at the site of administration	Fever	−	2022−02−15	2022−02−15	28 days	1 day	Recovery	Mild	Non−serious
Diffuse lesion	Kobe−08	Female	12	having	1	4	Pain	General and systemic disorders and conditions at the site of administration	Pain	−	2022−07−27	2022−07−29	1 day	3 days	Recovery	Mild	Non−serious
					2	2	Blister	Skin and subcutaneous tissue disorders	Blister	−	2022−07−29	2022−08−08	3 days	11 days	Recovery	Mild	Non−serious
					3	4	Urine occult blood positive	Clinical examination	Urine occult blood positive	−	2022−07−28	2022−07−29	2 days	2 days	Recovery	Mild	Non−serious
Diffuse lesion	Kobe−10	Male	13	having	1	4	Pain	General and systemic disorders and conditions at the site of administration	Pain	−	2022−09−07	2022−09−16	1 day	10 days	Recovery	Mild	Non−serious
					2	1	Hemoglobinuria	Renal and urinary tract disorders	Hemoglobinuria	−	2022−09−07	2022−09−07	1 day	1 day	Recovery	Mild	Non−serious
					3	4	Urine output decreased	Clinical examination	Urine output decreased	−	2022−09−07	2022−09−08	1 day	2 days	Recovery	Mild	Non−serious

All adverse events
Analysis Subject: SAS
*1 1: Hemoglobinuria, 2: Blistering, 3: Swelling, 4: Other
*2 Number of days from the date of the first administration

MedDRA/J Ver. 26.0

Lesion	Case number	Gender	Age (years)	Presence or absence of onset	No.	Classification *1	Condition listed by Physician	System Organ Class	Preferred Term	Investigational drug treatment	Other treatment	Causal relationship	Reasons for no causal relationship	Other details	Comment
Diffuse lesion	Kobe-04	Male	32	having	1	1	Hemoglobinuria	Renal and urinary tract disorders	Hemoglobinuria	Not applicable	Treatment implementation	Causal relationship exists	–	–	–
					2	4	Uulnar nerve palsy	Nervous System Disorders	Uulnar nerve palsy	Not applicable	Treatment implementation	Causal relationship exists	–	–	No further follow-up is needed as the patient will be followed up on an outpatient basis from now on.
					3	4	Pain	General and systemic disorders and conditions at the site of administration	Pain	Not applicable	Treatment implementation	Causal relationship exists	–	–	–
					4	4	Epigastric Pain	Gastrointestinal disorder	Epigastric Pain	Not applicable	Treatment implementation	No causal relationship	Due to concomittreatment implementation medications	–	–
					5	4	Lightheadedness	Nervous System Disorders	Floating dizziness	Not applicable	No treatment	No causal relationship	Due to concomitImplementation medications	–	–
Diffuse lesion	Kobe-05	Female	56	having	1	4	Pain	General and systemic disorders and conditions at the site of administration	Pain	Not applicable	Treatment implementation	Causal relationship exists	–	–	–
Diffuse lesion	Kobe-06	Female	53	having	1	4	Pain	General and systemic disorders and conditions at the site of administration	Pain	Not applicable	Treatment implementation	Causal relationship exists	–	–	–
					2	4	Urine occult blood positive	Clinical examination	Urine occult blood positive	Not applicable	No treatment	Causal relationship exists	–	–	–
					3	4	Fever	General and systemic disorders and conditions at the site of administration	Fever	Not applicable	Treatment implementation	No causal relationship	Accidental	–	–
Diffuse lesion	Kobe-08	Female	12	having	1	4	Pain	General and systemic disorders and conditions at the site of administration	Pain	Not applicable	Treatment implementation	Causal relationship exists	–	–	–
					2	2	Blister	Skin and subcutaneous tissue disorders	Blister	Not applicable	Treatment implementation	Causal relationship exists	–	–	–
					3	4	Urine occult blood positive	Clinical examination	Urine occult blood positive	Not applicable	No treatment	Causal relationship exists	–	–	–
Diffuse lesion	Kobe-10	Male	13	having	1	4	Pain	General and systemic disorders and conditions at the site of administration	Pain	Not applicable	Treatment implementation	Causal relationship exists	–	–	–
					2	1	Hemoglobinuria	Renal and urinary tract disorders	Hemoglobinuria	Not applicable	Treatment implementation	Causal relationship exists	–	–	–
					3	4	Urine output decreased	Clinical examination	Urine output decreased	Not applicable	Treatment implementation	Causal relationship exists	–	–	–

All adverse events
Analysis Subject: SAS
*1 1: Hemoglobinuria, 2: Blistering, 3: Swelling, 4: Other
*2 Number of days from the date of the first administration

Lesion	Case number	Gender	Age (years)	Presence or absence of onset	No.	Classification *1	Condition listed by Physician	System Organ Class	Preferred Term	Significant adverse events	Date of onset	Date of confirmation/ disappearance date/death	Number of days*2	Duration	Outcome	Severity	Seriousness
Diffuse lesion	Seiiku-01	Male	5	having	1	4	Upper respiratory inflammation	Respiratory, thoracic and mediastinal disorders	Inflammation of the upper respiratory tract	-	2021-12-20	2021-12-23	-29 days	4 days	Recovery	Mild	Non-serious
					2	4	Haematoma	Vascular disorder	Haematoma	-	2021-12-22	2021-12-29	-27 days	8 days	Recovery	Mild	Non-serious
					3	1	Hemoglobinuria	Renal and urinary tract disorders	Hemoglobinuria	-	2022-01-18	2022-01-19	1 day	2 days	Recovery	Mild	Non-serious
Diffuse lesion	Osaka-01	Male	25	having	1	4	Pain	General and systemic disorders and conditions at the site of administration	Pain	-	2021-02-02	2021-04-28	1 day	86 days	Recovery	Moderate	Non-serious
					2	1	Hemoglobinuria	Renal and urinary tract disorders	Hemoglobinuria	-	2021-02-02	2021-02-05	1 day	4 days	Recovery	Mild	Non-serious
					3	4	Urinary sugar	Clinical examination	Urinary glucose	-	2021-02-03	2021-02-05	2 days	3 days	Recovery	Mild	Non-serious
					4	4	Subcutaneous hemorrhage	Skin and subcutaneous tissue disorders	Subcutaneous hemorrhage	-	2021-02-03	2021-04-28	2 days	85 days	Recovery	Mild	Non-serious
					5	3	Swelling	General and systemic disorders and conditions at the site of administration	Swelling	-	2021-02-02	2021-03-03	1 day	30 days	Recovery	Mild	Non-serious
Diffuse lesion	Osaka-02	Male	6	having	1	4	Enlargement of the tonsils	Respiratory, thoracic and mediastinal disorders	Enlargement of the tonsils	-	2021-07-21	2021-07-27	-6 days	7 days	Recovery	Mild	Non-serious
					2	1	Hemoglobinuria	Renal and urinary tract disorders	Hemoglobinuria	-	2021-07-27	2021-07-30	1 day	4 days	Recovery	Moderate	Non-serious
					3	4	Pain	General and systemic disorders and conditions at the site of administration	Pain	-	2021-07-27	2021-08-25	1 day	30 days	Recovery	Mild	Non-serious
					4	4	Nausea	Gastrointestinal disorder	Nausea	-	2021-07-27	2021-07-27	1 day	1 day	Recovery	Mild	Non-serious
					5	4	Vomiting	Gastrointestinal disorder	Vomiting	-	2021-07-27	2021-07-27	1 day	1 day	Recovery	Mild	Non-serious

All adverse events
Analysis Subject: SAS
*1 1: Hemoglobinuria, 2: Blistering, 3: Swelling, 4: Other
*2 Number of days from the date of the first administration

MedDRA/J Ver. 26.0

Lesion	Case number	Gender	Age (years)	Presence or absence of onset	No.	Classification *1	Condition listed by Physician	System Organ Class	Preferred Term	Investigational drug treatment	Other treatment	Causal relationship	Reasons for no causal relationship	Other details	Comment
Diffuse lesion	Seiiku-01	Male	5	having	1	4	Upper respiratory inflammation	Respiratory, thoracic and mediastinal disorders	Inflammation of the upper respiratory tract	Not applicable	No treatment	No causal relationship	Accidental	–	–
					2	4	Haematoma	Vascular disorder	Haematoma	Not applicable	No treatment	No causal relationship	Accidental	–	–
					3	1	Hemoglobinuria	Renal and urinary tract disorders	Hemoglobinuria	Not applicable	Treatment implementation	Causal relationship exists	–	–	Haptoglobin was administered.
Diffuse lesion	Osaka-01	Male	25	having	1	4	Pain	General and systemic disorders and conditions at the site of administration	Pain	Not applicable	Treatment implementation	Causal relationship exists	–	–	–
					2	1	Hemoglobinuria	Renal and urinary tract disorders	Hemoglobinuria	Not applicable	Treatment implementation	Causal relationship exists	–	–	–
					3	4	Urinary sugar	Clinical examination	Urinary glucose	Not applicable	No treatment	Causal relationship exists	–	–	–
					4	4	Subcutaneous hemorrhage	Skin and subcutaneous tissue disorders	Subcutaneous hemorrhage	Not applicable	No treatment	Causal relationship exists	–	–	–
					5	3	Swelling	General and systemic disorders and conditions at the site of administration	Swelling	Not applicable	No treatment	Causal relationship exists	–	–	–
Diffuse lesion	Osaka-02	Male	6	having	1	4	Enlargement of the tonsils	Respiratory, thoracic and mediastinal disorders	Enlargement of the tonsils	Not applicable	Treatment implementation	No causal relationship	Accidental	–	–
					2	1	Hemoglobinuria	Renal and urinary tract disorders	Hemoglobinuria	Not applicable	Treatment implementation	Causal relationship exists	–	–	–
					3	4	Pain	General and systemic disorders and conditions at the site of administration	Pain	Not applicable	Treatment implementation	Causal relationship exists	–	–	–
					4	4	Nausea	Gastrointestinal disorder	Nausea	Unchanged	No treatment	No causal relationship	Due to anesthesia for sclerotherapy	–	–
					5	4	Vomiting	Gastrointestinal disorder	Vomiting	Unchanged	No treatment	No causal relationship	Due to anesthesia for sclerotherapy	–	–

All adverse events
Analysis Subject: SAS
*1 1: Hemoglobinuria, 2: Blistering, 3: Swelling, 4: Other
*2 Number of days from the date of the first administration

Lesion	Case number	Gender	Age (years)	Presence or absence of onset	No.	Classification *1	Condition listed by Physician	System Organ Class	Preferred Term	Significant adverse events	Date of onset	Date of confirmation/ disappearance date/death	Number of days*2	Duration	Outcome	Severity	Seriousness
Diffuse lesion	Osaka-04	Male	25	having	1	4	Postoperative Pain	General and systemic disorders and conditions at the site of administration	Pain	-	2021-11-16	2022-02-09	1 day	86 days	Recovery	Mild	Non-serious
Diffuse lesion	Osaka-05	Female	17	having	1	4	Postoperative Pain	General and systemic disorders and conditions at the site of administration	Pain	-	2021-12-21	2022-01-05	1 day	16 days	Recovery	Moderate	Non-serious
					2	4	Subcutaneous hemorrhage	Skin and subcutaneous tissue disorders	Subcutaneous hemorrhage	-	2021-12-23	2022-01-05	3 days	14 days	Recovery	Mild	Non-serious
Diffuse lesion	Tokyo-02	Female	16	having	1	4	Impairment of liver function	Hepatobiliary System Disorders	Abnormal liver function	-	2021-08-02	2021-08-12	6 days	11 days	Recovery	Mild	Non-serious
					2	4	Elevated CK	Clinical examination	Creatine phosphokinase increased	-	2021-07-29	2021-08-02	2 days	5 days	Recovery	Mild	Non-serious
					3	4	Postoperative Pain	General and systemic disorders and conditions at the site of administration	Pain	-	2021-07-28	2021-08-09	1 day	13 days	Recovery	Mild	Non-serious
Diffuse lesion	Tokyo-03	Female	59	having	1	1	Hemoglobinuria	Renal and urinary tract disorders	Hemoglobinuria	-	2021-12-22	2021-12-23	1 day	2 days	Recovery	Mild	Non-serious
Diffuse lesion	Shinshu-01	Female	14	having	1	1	Hemoglobinuria	Renal and urinary tract disorders	Hemoglobinuria	-	2021-08-11	2021-08-14	1 day	4 days	Recovery	Mild	Non-serious
					2	4	Postoperative Pain	General and systemic disorders and conditions at the site of administration	Pain	-	2021-08-11	2021-08-14	1 day	4 days	Recovery	Mild	Non-serious
					3	4	Residual subcutaneous hemorrhage	Skin and subcutaneous tissue disorders	Subcutaneous hemorrhage	-	2021-08-24	2021-11-09	14 days	78 days	Light	Mild	Non-serious
Diffuse lesion	Shinshu-03	Female	52	having	1	4	Postoperative Pain	General and systemic disorders and conditions at the site of administration	Pain	-	2022-02-18	2022-03-08	3 days	19 days	Recovery	Mild	Non-serious
					2	1	Hemoglobinuria	Renal and urinary tract disorders	Hemoglobinuria	-	2022-02-16	2022-02-18	1 day	3 days	Recovery	Mild	Non-serious

All adverse events
Analysis Subject: SAS
*1 1: Hemoglobinuria, 2: Blistering, 3: Swelling, 4: Other
*2 Number of days from the date of the first administration

MedDRA/J Ver. 26.0

Lesion	Case number	Gender	Age (years)	Presence or absence of onset	No.	Classification *1	Condition listed by Physician	System Organ Class	Preferred Term	Investigational drug treatment	Other treatment	Causal relationship	Reasons for no causal relationship	Other details	Comment
Diffuse lesion	Osaka-04	Male	25	having	1	4	Postoperative Pain	General and systemic disorders and conditions at the site of administration	Pain	Not applicable	Treatment implementation	Causal relationship exists	-	-	-
Diffuse lesion	Osaka-05	Female	17	having	1	4	Postoperative Pain	General and systemic disorders and conditions at the site of administration	Pain	Not applicable	Treatment implementation	Causal relationship exists	-	-	-
					2	4	Subcutaneous hemorrhage	Skin and subcutaneous tissue disorders	Subcutaneous hemorrhage	Not applicable	No treatment	Causal relationship exists	-	-	-
Diffuse lesion	Tokyo-02	Female	16	having	1	4	Impairment of liver function	Hepatobiliary System Disorders	Abnormal liver function	Not applicable	No treatment	No causal relationship	Due to concomitImplementation medications	-	Because it is thought to be an effect of drugs used for postoperative pain
					2	4	Elevated CK	Clinical examination	Creatine phosphokinase increased	Not applicable	No treatment	Causal relationship exists	-	-	The event has been confirmed to have occurred with the administration of oldamine, and a causal relationship was also considered from the time line.
					3	4	Postoperative Pain	General and systemic disorders and conditions at the site of administration	Pain	Not applicable	Treatment implementation	Causal relationship exists	-	-	-
Diffuse lesion	Tokyo-03	Female	59	having	1	1	Hemoglobinuria	Renal and urinary tract disorders	Hemoglobinuria	Not applicable	Treatment implementation	Causal relationship exists	-	-	-
Diffuse lesion	Shinshu-01	Female	14	having	1	1	Hemoglobinuria	Renal and urinary tract disorders	Hemoglobinuria	Not applicable	Treatment implementation	Causal relationship exists	-	-	-
					2	4	Postoperative Pain	General and systemic disorders and conditions at the site of administration	Pain	Not applicable	Treatment implementation	Causal relationship exists	-	-	-
					3	4	Residual subcutaneous hemorrhage	Skin and subcutaneous tissue disorders	Subcutaneous hemorrhage	Not applicable	No treatment	Causal relationship exists	-	-	Decided that no follow-up was necessary due to follow-up with usual medical care at the last visit (2021.11.9).
Diffuse lesion	Shinshu-03	Female	52	having	1	4	Postoperative Pain	General and systemic disorders and conditions at the site of administration	Pain	Not applicable	Treatment implementation	Causal relationship exists	-	-	-
					2	1	Hemoglobinuria	Renal and urinary tract disorders	Hemoglobinuria	Not applicable	Treatment implementation	Causal relationship exists	-	-	-

All adverse events
Analysis Subject: SAS
*1 1: Hemoglobinuria, 2: Blistering, 3: Swelling, 4: Other
*2 Number of days from the date of the first administration

Lesion	Case number	Gender	Age (years)	Presence or absence of onset	No.	Classification *1	Condition listed by Physician	System Organ Class	Preferred Term	Significant adverse events	Date of onset	Date of confirmation/ disappearance date/death	Number of days*2	Duration	Outcome	Severity	Seriousness
Diffuse lesion	Shinshu-04	Male	10	having	1	4	Urethral injury	Injury, poisoning and treatment complications	Urethral injury	−	2022-08-24	2022-08-26	1 day	3 days	Recovery	Mild	Non-serious
					2	1	Hemoglobinuria	Renal and urinary tract disorders	Hemoglobinuria	−	2022-08-24	2022-08-26	1 day	3 days	Recovery	Mild	Non-serious
					3	4	Postoperative Pain	General and systemic disorders and conditions at the site of administration	Pain	−	2022-08-24	2022-11-15	1 day	84 days	Recovery	Mild	Non-serious
					4	3	Swelling	General and systemic disorders and conditions at the site of administration	Swelling	−	2022-08-25	2022-11-15	2 days	83 days	Recovery	Mild	Non-serious
Diffuse lesion	Shinshu-05	Female	10	having	1	1	Hemoglobinuria	Renal and urinary tract disorders	Hemoglobinuria	−	2022-12-07	2022-12-08	1 day	2 days	Recovery	Mild	Non-serious
					2	4	Postoperative Pain	General and systemic disorders and conditions at the site of administration	Pain	−	2022-12-07	2023-01-17	1 day	42 days	Recovery	Mild	Non-serious
					3	4	Subcutaneous hemorrhage	Skin and subcutaneous tissue disorders	Subcutaneous hemorrhage	−	2022-12-07	2022-12-09	1 day	3 days	Light	Mild	Non-serious
					4	3	Swelling	General and systemic disorders and conditions at the site of administration	Swelling	−	2022-12-07	2023-01-17	1 day	42 days	Recovery	Mild	Non-serious
					5	4	COVID-19 PCR positive	Infectious and parasitic diseases	COVID-19	−	2022-12-29	2023-01-06	23 days	9 days	Recovery	Mild	Non-serious

All adverse events
Analysis Subject: SAS
*1 1: Hemoglobinuria, 2: Blistering, 3: Swelling, 4: Other
*2 Number of days from the date of the first administration

MedDRA/J Ver. 26.0

Lesion	Case number	Gender	Age (years)	Presence or absence of onset	No.	Classification *1	Condition listed by Physician	System Organ Class	Preferred Term	Investigational drug treatment	Other treatment	Causal relationship	Reasons for no causal relationship	Other details	Comment
Diffuse lesion	Shinshu-04	Male	10	having	1	4	Urethral injury	Injury, poisoning and treatment complications	Urethral injury	Not applicable	No treatment	No causal relationship	Other	Resulting from insertion of urinary	–
					2	1	Hemoglobinuria	Renal and urinary tract disorders	Hemoglobinuria	Not applicable	Treatment implementation	Causal relationship exists	–	–	–
					3	4	Postoperative Pain	General and systemic disorders and conditions at the site of administration	Pain	Not applicable	Treatment implementation	Causal relationship exists	–	–	–
					4	3	Swelling	General and systemic disorders and conditions at the site of administration	Swelling	Not applicable	No treatment	Causal relationship exists	–	–	–
Diffuse lesion	Shinshu-05	Female	10	having	1	1	Hemoglobinuria	Renal and urinary tract disorders	Hemoglobinuria	Not applicable	Treatment implementation	Causal relationship exists	–	–	–
					2	4	Postoperative Pain	General and systemic disorders and conditions at the site of administration	Pain	Not applicable	Treatment implementation	Causal relationship exists	–	–	–
					3	4	Subcutaneous hemorrhage	Skin and subcutaneous tissue disorders	Subcutaneous hemorrhage	Not applicable	No treatment	Causal relationship exists	–	–	No treatment is required, and the trial is terminated because the patient is followed up in regular medical care.
					4	3	Swelling	General and systemic disorders and conditions at the site of administration	Swelling	Not applicable	No treatment	Causal relationship exists	–	–	–
					5	4	COVID-19 PCR positive	Infectious and parasitic diseases	COVID-19	Not applicable	Treatment implementation	No causal relationship	Accidental	–	–
