# Supplementary material for: Effect and safety of ethanolamine oleate in sclerotherapy in patients with difficult-to-resect venous malformations: A multicenter, single-arm study
Source: PLoS One. 2025 Jan 31;20(1):e0303130. doi: 10.1371/journal.pone.0303130 (PMC11785324; doi:10.1371/journal.pone.0303130)
Supplement: S5 Table — (PDF) [file pone.0303130.s008.pdf]

Adverse drug reactions
Analysis Subject: SAS
*1 1: Hemoglobinuria, 2: Blistering, 3: Swelling, 4: Other
*2 Number of days from the date of the first administration

Lesion	Case number	Gender	Age (years)	No.	Classification *1	Condition listed by Physician	System Organ Class	Preferred Term	Significant adverse events	Date of onset	Date of confirmation/ disappearance date/death date	Number of days*2	Duration	Outcome	Severity	Seriousness
Cystic lesion	Kyorin-01	Male	6	1	1	Hemoglobinuria	Renal and urinary tract disorders	Hemoglobinuria	-	2021-02-09	2021-02-10	1 day	2 days	Recovery	Mild	Non-serious
				2	3	Swelling	General and systemic disorders and conditions at the site of administration	Swelling	-	2021-02-09	2021-03-12	1 day	32 days	Recovery	Mild	Non-serious
				3	4	Pain	General and systemic disorders and conditions at the site of administration	Pain	-	2021-02-09	2021-02-19	1 day	11 days	Recovery	Mild	Non-serious
				4	4	Rubefaction (reddening of the skin)	Skin and subcutaneous tissue disorders	Erythema	-	2021-02-09	2021-02-19	1 day	11 days	Recovery	Mild	Non-serious
				6	4	CK High	Clinical examination	Creatine phosphokinase increased	-	2021-02-10	2021-02-19	2 days	10days	Recovery	Mild	Non-serious
				7	4	D-dimer high	Clinical examination	Fibrin D-dimer increased	-	2021-02-10	2021-02-19	2 days	10 days	Recovery	Moderate	Non-serious
				8	4	High amylase level	Clinical examination	Amylase increased	-	2021-02-10	2021-02-19	2 days	10 days	Recovery	Mild	Non-serious
				10	4	High FDP	Clinical examination	Fibrin degradation products increased	-	2021-02-10	2021-02-19	2 days	10 days	Recovery	Mild	Non-serious

Adverse drug reactions
Analysis Subject: SAS
*1 1: Hemoglobinuria, 2: Blistering, 3: Swelling, 4: Other
*2 Number of days from the date of the first administration

MedDRA/J Ver. 26.0

Lesion	Case number	Gender	Age (years)	No.	Classification *1	Condition listed by Physician	System Organ Class	Preferred Term	Investigational drug treatment	Other treatment	Causal relationship	Reasons for no causal relationship	Other details	Comment
Cystic lesion	Kyorin-01	Male	6	1	1	Hemoglobinuria	Renal and urinary tract disorders	Hemoglobinuria	Not applicable	Treatment implementation	Causal relationship exists	–	–	–
				2	3	Swelling	General and systemic disorders and conditions at the site of administration	Swelling	Not applicable	No treatment	Causal relationship exists	–	–	–
				3	4	Pain	General and systemic disorders and conditions at the site of administration	Pain	Not applicable	Treatment implementation	Causal relationship exists	–	–	–
				4	4	Rubefaction (reddening of the skin)	Skin and subcutaneous tissue disorders	Erythema	Not applicable	No treatment	Causal relationship exists	–	–	–
				6	4	CK High	Clinical examination	Creatine phosphokinase increased	Not applicable	No treatment	Causal relationship exists	–	–	–
				7	4	D-dimer high	Clinical examination	Fibrin D-dimer increased	Not applicable	No treatment	Causal relationship exists	–	–	–
				8	4	High amylase level	Clinical examination	Amylase increased	Not applicable	No treatment	Causal relationship exists	–	–	–
				10	4	High FDP	Clinical examination	Fibrin degradation products increased	Not applicable	No treatment	Causal relationship exists	–	–	–

Adverse drug reactions
Analysis Subject: SAS
*1 1: Hemoglobinuria, 2: Blistering, 3: Swelling, 4: Other
*2 Number of days from the date of the first administration

Lesion	Case number	Gender	Age (years)	No.	Classification *1	Condition listed by Physician	System Organ Class	Preferred Term	Significant adverse events	Date of onset	Date of confirmation/ disappearance date/death date	Number of days*2	Duration	Outcome	Severity	Seriousness
Cystic lesion	Kyorin-02	Female	31	1	3	Swelling	General and systemic disorders and conditions at the site of administration	Swelling	-	2021-03-26	2021-04-09	2 days	15 days	Recovery	Mild	Non-serious
				2	4	Pain	General and systemic disorders and conditions at the site of administration	Pain	-	2021-03-26	2021-04-22	2 days	28 days	Recovery	Mild	Non-serious
				3	4	Rubefaction (reddening of the skin)	Skin and subcutaneous tissue disorders	Erythema	-	2021-03-26	2021-04-09	2 days	15 days	Recovery	Mild	Non-serious
				4	4	CK High	Clinical examination	Creatine phosphokinase increased	-	2021-03-26	2021-04-09	2 days	15 days	Recovery	Mild	Non-serious
				5	4	CRP increased	Clinical examination	C-reactive protein increased	-	2021-03-27	2021-04-09	3 days	14 days	Recovery	Mild	Non-serious
Cystic lesion	Kyorin-03	Male	17	1	4	Pain	General and systemic disorders and conditions at the site of administration	Pain	-	2021-07-20	2021-08-06	1 day	18 days	Recovery	Mild	Non-serious
				2	4	Serum bilirubin increased	Clinical examination	Serum bilirubin increased	-	2021-07-21	2021-08-06	2 days	17 days	Recovery	Mild	Non-serious
Cystic lesion	Junten-01	Female	3	1	4	CPK increased	Clinical examination	Creatine phosphokinase increased	-	2021-03-18	2021-04-02	2 days	16 days	Recovery	Mild	Non-serious
Cystic lesion	Junten-02	Male	14	1	1	Hemoglobinuria	Renal and urinary tract disorders	Hemoglobinuria	-	2021-09-30	2021-10-01	1 day	2 days	Recovery	Mild	Non-serious

Adverse drug reactions
Analysis Subject: SAS
*1 1: Hemoglobinuria, 2: Blistering, 3: Swelling, 4: Other
*2 Number of days from the date of the first administration

MedDRA/J Ver. 26.0

Lesion	Case number	Gender	Age (years)	No.	Classification *1	Condition listed by Physician	System Organ Class	Preferred Term	Investigational drug treatment	Other treatment	Causal relationship	Reasons for no causal relationship	Other details	Comment
Cystic lesion	Kyorin-02	Female	31	1	3	Swelling	General and systemic disorders and conditions at the site of administration	Swelling	Not applicable	No treatment	Causal relationship exists	–	–	–
				2	4	Pain	General and systemic disorders and conditions at the site of administration	Pain	Not applicable	Treatment implementation	Causal relationship exists	–	–	–
				3	4	Rubefaction (reddening of the skin)	Skin and subcutaneous tissue disorders	Erythema	Not applicable	No treatment	Causal relationship exists	–	–	–
				4	4	CK High	Clinical examination	Creatine phosphokinase increased	Not applicable	No treatment	Causal relationship exists	–	–	–
				5	4	CRP increased	Clinical examination	C-reactive protein increased	Not applicable	No treatment	Causal relationship exists	–	–	–
Cystic lesion	Kyorin-03	Male	17	1	4	Pain	General and systemic disorders and conditions at the site of administration	Pain	Not applicable	Treatment implementation	Causal relationship exists	–	–	–
				2	4	Serum bilirubin increased	Clinical examination	Serum bilirubin increased	Not applicable	No treatment	Causal relationship exists	–	–	–
Cystic lesion	Junten-01	Female	3	1	4	CPK increased	Clinical examination	Creatine phosphokinase increased	Not applicable	No treatment	Causal relationship exists	–	–	–
Cystic lesion	Junten-02	Male	14	1	1	Hemoglobinuria	Renal and urinary tract disorders	Hemoglobinuria	Not applicable	Treatment implementation	Causal relationship exists	–	–	–

Adverse drug reactions
Analysis Subject: SAS
*1 1: Hemoglobinuria, 2: Blistering, 3: Swelling, 4: Other
*2 Number of days from the date of the first administration

Lesion	Case number	Gender	Age (years)	No.	Classification *1	Condition listed by Physician	System Organ Class	Preferred Term	Significant adverse events	Date of onset	Date of confirmation/ disappearance date/death date	Number of days*2	Duration	Outcome	Severity	Seriousness
Cystic lesion	Kobe-01	Female	78	1	4	Postoperative Pain	General and systemic disorders and conditions at the site of administration	Pain	-	2021-03-03	2021-03-05	1 day	3 days	Recovery	Mild	Non-serious
				3	3	Swelling	General and systemic disorders and conditions at the site of administration	Swelling	-	2021-03-03	2021-03-16	1 day	14 days	Recovery	Mild	Non-serious
Cystic lesion	Kobe-03	Male	5	1	4	Fever	General and systemic disorders and conditions at the site of administration	Fever	-	2021-08-12	2021-08-20	2 days	9 days	Recovery	Mild	Non-serious
				2	4	Pain	General and systemic disorders and conditions at the site of administration	Pain	-	2021-08-11	2021-08-20	1 day	10 days	Recovery	Mild	Non-serious
Cystic lesion	Kobe-09	Male	8	1	4	Pain	General and systemic disorders and conditions at the site of administration	Pain	-	2022-08-10	2022-08-15	1 day	6 days	Recovery	Mild	Non-serious
				2	4	Urine occult blood positive	Clinical examination	Urine occult blood positive	-	2022-08-11	2022-08-12	2 days	2 days	Recovery	Mild	Non-serious
				4	4	Fever	General and systemic disorders and conditions at the site of administration	Fever	-	2022-08-10	2022-08-12	1 day	3 days	Recovery	Mild	Non-serious
Cystic lesion	Kobe-11	Male	44	1	4	Pain	General and systemic disorders and conditions at the site of administration	Pain	-	2023-01-25	2023-02-01	1 day	eight days	Recovery	Mild	Non-serious

Adverse drug reactions
Analysis Subject: SAS
*1 1: Hemoglobinuria, 2: Blistering, 3: Swelling, 4: Other
*2 Number of days from the date of the first administration

MedDRA/J Ver. 26.0

Lesion	Case number	Gender	Age (years)	No.	Classification *1	Condition listed by Physician	System Organ Class	Preferred Term	Investigational drug treatment	Other treatment	Causal relationship	Reasons for no causal relationship	Other details	Comment
Cystic lesion	Kobe-01	Female	78	1	4	Postoperative Pain	General and systemic disorders and conditions at the site of administration	Pain	Not applicable	Treatment implementation	Causal relationship exists	–	–	–
				3	3	Swelling	General and systemic disorders and conditions at the site of administration	Swelling	Not applicable	No treatment	Causal relationship exists	–	–	–
Cystic lesion	Kobe-03	Male	5	1	4	Fever	General and systemic disorders and conditions at the site of administration	Fever	Not applicable	Treatment implementation	Causal relationship exists	–	–	–
				2	4	Pain	General and systemic disorders and conditions at the site of administration	Pain	Not applicable	Treatment implementation	Causal relationship exists	–	–	–
Cystic lesion	Kobe-09	Male	8	1	4	Pain	General and systemic disorders and conditions at the site of administration	Pain	Not applicable	Treatment implementation	Causal relationship exists	–	–	–
				2	4	Urine occult blood positive	Clinical examination	Urine occult blood positive	Not applicable	No treatment	Causal relationship exists	–	–	–
				4	4	Fever	General and systemic disorders and conditions at the site of administration	Fever	Not applicable	No treatment	Causal relationship exists	–	–	–
Cystic lesion	Kobe-11	Male	44	1	4	Pain	General and systemic disorders and conditions at the site of administration	Pain	Not applicable	Treatment implementation	Causal relationship exists	–	–	–

Adverse drug reactions
Analysis Subject: SAS
*1 1: Hemoglobinuria, 2: Blistering, 3: Swelling, 4: Other
*2 Number of days from the date of the first administration

Lesion	Case number	Gender	Age (years)	No.	Classification *1	Condition listed by Physician	System Organ Class	Preferred Term	Significant adverse events	Date of onset	Date of confirmation/ disappearance date/death date	Number of days*2	Duration	Outcome	Severity	Seriousness
Cystic lesion	Osaka-03	Female	50	1	3	Swelling	General and systemic disorders and conditions at the site of administration	Swelling	-	2021-10-19	2021-11-17	1 day	30 days	Recovery	Moderate	Non-serious
				2	4	Pain	General and systemic disorders and conditions at the site of administration	Pain	-	2021-10-19	2022-01-12	1 day	86 days	Recovery	Mild	Non-serious
Cystic lesion	Osaka-06	Male	5	1	1	Hemoglobinuria	Renal and urinary tract disorders	Hemoglobinuria	-	2022-05-24	2022-05-27	1 day	4 days	Recovery	Moderate	Non-serious
				2	4	Subcutaneous hemorrhage	Skin and subcutaneous tissue disorders	Subcutaneous hemorrhage	-	2022-05-25	2022-06-08	2 days	15 days	Recovery	Mild	Non-serious
				3	4	Pain	General and systemic disorders and conditions at the site of administration	Pain	-	2022-05-24	2022-05-25	1 day	2 days	Recovery	Mild	Non-serious
Cystic lesion	Tokyo-01	Female	26	1	1	Hemoglobinuria	Renal and urinary tract disorders	Hemoglobinuria	-	2021-03-10	2021-03-10	1 day	1 day	Recovery	Mild	Non-serious
Cystic lesion	Tokyo-04	Male	33	1	1	Hemoglobinuria	Renal and urinary tract disorders	Hemoglobinuria	-	2022-05-27	2022-05-27	1 day	1 day	Recovery	Mild	Non-serious
Cystic lesion	Tokyo-05	Male	11	2	4	Postoperative Pain	General and systemic disorders and conditions at the site of administration	Pain	-	2022-10-21	2022-10-30	3 days	10 days	Recovery	Mild	Non-serious

Adverse drug reactions
Analysis Subject: SAS
*1 1: Hemoglobinuria, 2: Blistering, 3: Swelling, 4: Other
*2 Number of days from the date of the first administration

MedDRA/J Ver. 26.0

Lesion	Case number	Gender	Age (years)	No.	Classification *1	Condition listed by Physician	System Organ Class	Preferred Term	Investigational drug treatment	Other treatment	Causal relationship	Reasons for no causal relationship	Other details	Comment
Cystic lesion	Osaka-03	Female	50	1	3	Swelling	General and systemic disorders and conditions at the site of administration	Swelling	Not applicable	No treatment	Causal relationship exists	–	–	–
				2	4	Pain	General and systemic disorders and conditions at the site of administration	Pain	Not applicable	Treatment implementation	Causal relationship exists	–	–	–
Cystic lesion	Osaka-06	Male	5	1	1	Hemoglobinuria	Renal and urinary tract disorders	Hemoglobinuria	Not applicable	Treatment implementation	Causal relationship exists	–	–	–
				2	4	Subcutaneous hemorrhage	Skin and subcutaneous tissue disorders	Subcutaneous hemorrhage	Not applicable	No treatment	Causal relationship exists	–	–	–
				3	4	Pain	General and systemic disorders and conditions at the site of administration	Pain	Not applicable	No treatment	Causal relationship exists	–	–	–
Cystic lesion	Tokyo-01	Female	26	1	1	Hemoglobinuria	Renal and urinary tract disorders	Hemoglobinuria	Not applicable	Treatment implementation	Causal relationship exists	–	–	–
Cystic lesion	Tokyo-04	Male	33	1	1	Hemoglobinuria	Renal and urinary tract disorders	Hemoglobinuria	Not applicable	Treatment implementation	Causal relationship exists	–	–	–
Cystic lesion	Tokyo-05	Male	11	2	4	Postoperative Pain	General and systemic disorders and conditions at the site of administration	Pain	Not applicable	Treatment implementation	Causal relationship exists	–	–	–

Adverse drug reactions
Analysis Subject: SAS
*1 1: Hemoglobinuria, 2: Blistering, 3: Swelling, 4: Other
*2 Number of days from the date of the first administration

Lesion	Case number	Gender	Age (years)	No.	Classification *1	Condition listed by Physician	System Organ Class	Preferred Term	Significant adverse events	Date of onset	Date of confirmation/ disappearance date/death date	Number of days*2	Duration	Outcome	Severity	Seriousness
Cystic lesion	Shinshu-02	Female	37	1	1	Hemoglobinuria	Renal and urinary tract disorders	Hemoglobinuria	–	2021-08-18	2021-09-14	1 day	28 days	Recovery	Mild	Non-serious
				2	4	Postoperative Pain	General and systemic disorders and conditions at the site of administration	Pain	–	2021-08-18	2021-09-14	1 day	28 days	Recovery	Severe	Non-serious
				3	3	Swelling	General and systemic disorders and conditions at the site of administration	Swelling	–	2021-08-19	2021-08-31	2 days	13 days	Recovery	Mild	Non-serious
				4	4	Subcutaneous hemorrhage	Skin and subcutaneous tissue disorders	Subcutaneous hemorrhage	–	2021-08-19	2021-08-31	2 days	13 days	Recovery	Mild	Non-serious
Cystic lesion	Keio-01	Female	27	1	1	Hemoglobinuria	Renal and urinary tract disorders	Hemoglobinuria	–	2021-04-08	2021-04-09	1 day	2 days	Recovery	Mild	Non-serious
Cystic lesion	Keio-02	Male	39	1	1	Hemoglobinuria	Renal and urinary tract disorders	Hemoglobinuria	–	2021-11-17	2021-11-17	1 day	1 day	Recovery	Mild	Non-serious
				2	4	Nnumbness	Nervous System Disorders	Sensory paralysis	–	2021-11-17	2021-11-17	1 day	1 day	Recovery	Mild	Non-serious
Cystic lesion	Keio-03	Female	16	1	1	Hemoglobinuria	Renal and urinary tract disorders	Hemoglobinuria	–	2021-12-22	2022-01-05	1 day	15 days	Recovery	Mild	Non-serious
				2	4	Pain	General and systemic disorders and conditions at the site of administration	Pain	–	2021-12-22	2022-03-23	1 day	92 days	Recovery	Mild	Non-serious

Adverse drug reactions
Analysis Subject: SAS
*1 1: Hemoglobinuria, 2: Blistering, 3: Swelling, 4: Other
*2 Number of days from the date of the first administration

MedDRA/J Ver. 26.0

Lesion	Case number	Gender	Age (years)	No.	Classification *1	Condition listed by Physician	System Organ Class	Preferred Term	Investigational drug treatment	Other treatment	Causal relationship	Reasons for no causal relationship	Other details	Comment
Cystic lesion	Shinshu-02	Female	37	1	1	Hemoglobinuria	Renal and urinary tract disorders	Hemoglobinuria	Not applicable	Treatment implementation	Causal relationship exists	–	–	–
				2	4	Postoperative Pain	General and systemic disorders and conditions at the site of administration	Pain	Not applicable	Treatment implementation	Causal relationship exists	–	–	–
				3	3	Swelling	General and systemic disorders and conditions at the site of administration	Swelling	Not applicable	No treatment	Causal relationship exists	–	–	–
				4	4	Subcutaneous hemorrhage	Skin and subcutaneous tissue disorders	Subcutaneous hemorrhage	Not applicable	No treatment	Causal relationship exists	–	–	–
Cystic lesion	Keio-01	Female	27	1	1	Hemoglobinuria	Renal and urinary tract disorders	Hemoglobinuria	Not applicable	Treatment implementation	Causal relationship exists	–	–	–
Cystic lesion	Keio-02	Male	39	1	1	Hemoglobinuria	Renal and urinary tract disorders	Hemoglobinuria	Not applicable	Treatment implementation	Causal relationship exists	–	–	–
				2	4	Nnumbness	Nervous System Disorders	Sensory paralysis	Not applicable	No treatment	Causal relationship exists	–	–	–
Cystic lesion	Keio-03	Female	16	1	1	Hemoglobinuria	Renal and urinary tract disorders	Hemoglobinuria	Not applicable	Treatment implementation	Causal relationship exists	–	–	–
				2	4	Pain	General and systemic disorders and conditions at the site of administration	Pain	Not applicable	Treatment implementation	Causal relationship exists	–	–	–

Adverse drug reactions
Analysis Subject: SAS
*1 1: Hemoglobinuria, 2: Blistering, 3: Swelling, 4: Other
*2 Number of days from the date of the first administration

Lesion	Case number	Gender	Age (years)	No.	Classification *1	Condition listed by Physician	System Organ Class	Preferred Term	Significant adverse events	Date of onset	Date of confirmation/ disappearance date/death date	Number of days*2	Duration	Outcome	Severity	Seriousness
Cystic lesion	Keio-04	Male	22	2	4	Headache	Nervous System Disorders	Headache	–	2023-01-12	2023-01-13	2 days	2 days	Recovery	Mild	Non-serious
				3	4	Fever	General and systemic disorders and conditions at the site of administration	Fever	–	2023-01-12	2023-01-22	2 days	11 days	Recovery	Mild	Non-serious
Diffuse lesion	Kyorin-04	Male	24	1	1	Hemoglobinuria	Renal and urinary tract disorders	Hemoglobinuria	–	2021-11-25	2021-11-26	1 day	2 days	Recovery	Mild	Non-serious
				2	4	Pain	General and systemic disorders and conditions at the site of administration	Pain	–	2021-11-25	2021-11-27	1 day	3 days	Recovery	Mild	Non-serious
				3	4	CK High	Clinical examination	Creatine phosphokinase increased	–	2021-11-26	2021-12-10	2 days	15 days	Recovery	Moderate	Non-serious
Diffuse lesion	Kyorin-05	Female	22	1	1	Hemoglobinuria	Renal and urinary tract disorders	Hemoglobinuria	–	2022-03-17	2022-03-17	1 day	1 day	Recovery	Mild	Non-serious
				3	4	Pain Exacerbation	General and systemic disorders and conditions at the site of administration	Pain	–	2022-03-17	2022-03-19	1 day	3 days	Recovery	Mild	Non-serious
				4	4	Numbness	Nervous system disorders	Sensory paralysis	–	2022-03-17	2022-04-15	1 day	30 days	Recovery	Mild	Non-serious

Adverse drug reactions
Analysis Subject: SAS
*1 1: Hemoglobinuria, 2: Blistering, 3: Swelling, 4: Other
*2 Number of days from the date of the first administration

MedDRA/J Ver. 26.0

Lesion	Case number	Gender	Age (years)	No.	Classification *1	Condition listed by Physician	System Organ Class	Preferred Term	Investigational drug treatment	Other treatment	Causal relationship	Reasons for no causal relationship	Other details	Comment
Cystic lesion	Keio-04	Male	22	2	4	Headache	Nervous System Disorders	Headache	Not applicable	Treatment implementation	Causal relationship exists	–	–	–
				3	4	Fever	General and systemic disorders and conditions at the site of administration	Fever	Not applicable	Treatment implementation	Causal relationship exists	–	–	–
Diffuse lesion	Kyorin-04	Male	24	1	1	Hemoglobinuria	Renal and urinary tract disorders	Hemoglobinuria	Not applicable	Treatment implementation	Causal relationship exists	–	–	–
				2	4	Pain	General and systemic disorders and conditions at the site of administration	Pain	Not applicable	Treatment implementation	Causal relationship exists	–	–	–
				3	4	CK High	Clinical examination	Creatine phosphokinase increased	Not applicable	No treatment	Causal relationship exists	–	–	–
Diffuse lesion	Kyorin-05	Female	22	1	1	Hemoglobinuria	Renal and urinary tract disorders	Hemoglobinuria	Not applicable	Treatment implementation	Causal relationship exists	–	–	–
				3	4	Pain Exacerbation	General and systemic disorders and conditions at the site of administration	Pain	Not applicable	Treatment implementation	Causal relationship exists	–	–	Inflammation due to local injection of the sclerosing agent, which is Causal relationship exists.
				4	4	Numbness	Nervous system disorders	Sensory paralysis	Not applicable	No treatment	Causal relationship exists	–	–	–

Adverse drug reactions
Analysis Subject: SAS
*1 1: Hemoglobinuria, 2: Blistering, 3: Swelling, 4: Other
*2 Number of days from the date of the first administration

Lesion	Case number	Gender	Age (years)	No.	Classification *1	Condition listed by Physician	System Organ Class	Preferred Term	Significant adverse events	Date of onset	Date of confirmation/ disappearance date/death date	Number of days*2	Duration	Outcome	Severity	Seriousness
Diffuse lesion	Kyorin-06	Male	14	1	1	Hemoglobinuria	Renal and urinary tract disorders	Hemoglobinuria	-	2022-03-29	2022-03-31	1 day	3 days	Recovery	Mild	Non-serious
				2	4	Pain	General and systemic disorders and conditions at the site of administration	Pain	-	2022-03-29	2022-06-24	1 day	88 days	Recovery	Moderate	Non-serious
				3	4	Rubefaction (reddening of the skin)	Skin and subcutaneous tissue disorders	Erythema	-	2022-03-30	2022-04-15	2 days	17 days	Recovery	Mild	Non-serious
				4	3	Swelling	General and systemic disorders and conditions at the site of administration	Swelling	-	2022-03-30	2022-04-15	2 days	17 days	Recovery	Mild	Non-serious
				5	4	Numbness	Nervous system disorders	Sensory paralysis	-	2022-03-30	2022-06-24	2 days	87 days	Light	Mild	Non-serious
Diffuse lesion	Kyorin-07	Female	6	1	4	Pain	General and systemic disorders and conditions at the site of administration	Pain	-	2022-07-26	2022-08-12	1 day	18 days	Recovery	Mild	Non-serious
				2	3	Swelling	General and systemic disorders and conditions at the site of administration	Swelling	-	2022-07-27	2022-08-12	2 days	17 days	Recovery	Mild	Non-serious
				3	4	Numbness	Nervous system disorders	Sensory paralysis	-	2022-07-27	2022-08-12	2 days	17 days	Recovery	Mild	Non-serious

Adverse drug reactions
Analysis Subject: SAS
*1 1: Hemoglobinuria, 2: Blistering, 3: Swelling, 4: Other
*2 Number of days from the date of the first administration

MedDRA/J Ver. 26.0

Lesion	Case number	Gender	Age (years)	No.	Classification *1	Condition listed by Physician	System Organ Class	Preferred Term	Investigational drug treatment	Other treatment	Causal relationship	Reasons for no causal relationship	Other details	Comment
Diffuse lesion	Kyorin-06	Male	14	1	1	Hemoglobinuria	Renal and urinary tract disorders	Hemoglobinuria	Not applicable	Treatment implementation	Causal relationship exists	–	–	–
				2	4	Pain	General and systemic disorders and conditions at the site of administration	Pain	Not applicable	Treatment implementation	Causal relationship exists	–	–	–
				3	4	Rubefaction (reddening of the skin)	Skin and subcutaneous tissue disorders	Erythema	Not applicable	Treatment implementation	Causal relationship exists	–	–	–
				4	3	Swelling	General and systemic disorders and conditions at the site of administration	Swelling	Not applicable	Treatment implementation	Causal relationship exists	–	–	–
				5	4	Numbness	Nervous system disorders	Sensory paralysis	Not applicable	Treatment implementation	Causal relationship exists	–	–	Since the Numbness is clearly improving, it is judged that the patient will fully recover with the remaining months of oral
Diffuse lesion	Kyorin-07	Female	6	1	4	Pain	General and systemic disorders and conditions at the site of administration	Pain	Not applicable	Treatment implementation	Causal relationship exists	–	–	–
				2	3	Swelling	General and systemic disorders and conditions at the site of administration	Swelling	Not applicable	Treatment implementation	Causal relationship exists	–	–	Kalonal fine granules 20%.
				3	4	Numbness	Nervous system disorders	Sensory paralysis	Not applicable	No treatment	Causal relationship exists	–	–	–

Adverse drug reactions
Analysis Subject: SAS
*1 1: Hemoglobinuria, 2: Blistering, 3: Swelling, 4: Other
*2 Number of days from the date of the first administration

Lesion	Case number	Gender	Age (years)	No.	Classification *1	Condition listed by Physician	System Organ Class	Preferred Term	Significant adverse events	Date of onset	Date of confirmation/ disappearance date/death date	Number of days*2	Duration	Outcome	Severity	Seriousness
Diffuse lesion	Kyorin-08	Female	26	1	1	Hemoglobinuria	Renal and urinary tract disorders	Hemoglobinuria	-	2022-10-27	2022-10-28	1 day	2 days	Recovery	Mild	Non-serious
				2	4	CK High	Clinical examination	Creatine phosphokinase increased	-	2022-10-28	2022-11-25	2 days	29 days	Recovery	Mild	Non-serious
Diffuse lesion	Kobe-02	Female	16	1	4	Pain	General and systemic disorders and conditions at the site of administration	Pain	-	2021-05-12	2021-08-02	1 day	83 days	Recovery	Mild	Non-serious
				3	3	Swelling	General and systemic disorders and conditions at the site of administration	Swelling	-	2021-05-13	2021-08-02	2 days	82 days	Recovery	Mild	Non-serious
				4	4	Urine occult blood positive	Clinical examination	Urine occult blood positive	-	2021-05-13	2021-05-14	2 days	2 days	Recovery	Mild	Non-serious
Diffuse lesion	Kobe-04	Male	32	1	1	Hemoglobinuria	Renal and urinary tract disorders	Hemoglobinuria	-	2021-08-18	2021-08-19	1 day	2 days	Recovery	Mild	Non-serious
				2	4	Uulnar nerve palsy	Nervous System Disorders	Uulnar nerve palsy	-	2021-08-19	2021-11-08	2 days	82 days	Light	Moderate	Non-serious
				3	4	Pain	General and systemic disorders and conditions at the site of administration	Pain	-	2021-08-18	2021-09-14	1 day	28 days	Recovery	Mild	Non-serious
Diffuse lesion	Kobe-05	Female	56	1	4	Pain	General and systemic disorders and conditions at the site of administration	Pain	-	2021-10-13	2021-10-26	1 day	14 days	Recovery	Mild	Non-serious

Adverse drug reactions
Analysis Subject: SAS
*1 1: Hemoglobinuria, 2: Blistering, 3: Swelling, 4: Other
*2 Number of days from the date of the first administration

MedDRA/J Ver. 26.0

Lesion	Case number	Gender	Age (years)	No.	Classification *1	Condition listed by Physician	System Organ Class	Preferred Term	Investigational drug treatment	Other treatment	Causal relationship	Reasons for no causal relationship	Other details	Comment
Diffuse lesion	Kyorin-08	Female	26	1	1	Hemoglobinuria	Renal and urinary tract disorders	Hemoglobinuria	Not applicable	Treatment implementation	Causal relationship exists	-	-	-
				2	4	CK High	Clinical examination	Creatine phosphokinase increased	Not applicable	No treatment	Causal relationship exists	-	-	-
Diffuse lesion	Kobe-02	Female	16	1	4	Pain	General and systemic disorders and conditions at the site of administration	Pain	Not applicable	Treatment implementation	Causal relationship exists	-	-	-
				3	3	Swelling	General and systemic disorders and conditions at the site of administration	Swelling	Not applicable	No treatment	Causal relationship exists	-	-	-
				4	4	Urine occult blood positive	Clinical examination	Urine occult blood positive	Not applicable	No treatment	Causal relationship exists	-	-	-
Diffuse lesion	Kobe-04	Male	32	1	1	Hemoglobinuria	Renal and urinary tract disorders	Hemoglobinuria	Not applicable	Treatment implementation	Causal relationship exists	-	-	-
				2	4	Uulnar nerve palsy	Nervous System Disorders	Uulnar nerve palsy	Not applicable	Treatment implementation	Causal relationship exists	-	-	No further follow-up is needed as the patient will be followed up on an outpatient basis from now
				3	4	Pain	General and systemic disorders and conditions at the site of administration	Pain	Not applicable	Treatment implementation	Causal relationship exists	-	-	-
Diffuse lesion	Kobe-05	Female	56	1	4	Pain	General and systemic disorders and conditions at the site of administration	Pain	Not applicable	Treatment implementation	Causal relationship exists	-	-	-

Adverse drug reactions
Analysis Subject: SAS
*1 1: Hemoglobinuria, 2: Blistering, 3: Swelling, 4: Other
*2 Number of days from the date of the first administration

Lesion	Case number	Gender	Age (years)	No.	Classification *1	Condition listed by Physician	System Organ Class	Preferred Term	Significant adverse events	Date of onset	Date of confirmation/ disappearance date/death date	Number of days*2	Duration	Outcome	Severity	Seriousness
Diffuse lesion	Kobe-06	Female	53	1	4	Pain	General and systemic disorders and conditions at the site of administration	Pain	-	2022-01-19	2022-02-10	1 day	23 days	Recovery	Mild	Non-serious
				2	4	Urine latent blood positivity	Clinical examination	Urine latent blood positivity	-	2022-01-20	2022-02-01	2 days	13 days	Recovery	Mild	Non-serious
Diffuse lesion	Kobe-08	Female	12	1	4	Pain	General and systemic disorders and conditions at the site of administration	Pain	-	2022-07-27	2022-07-29	1 day	3 days	Recovery	Mild	Non-serious
				2	2	blister	Skin and subcutaneous tissue disorders	blister	-	2022-07-29	2022-08-08	3 days	11 days	Recovery	Mild	Non-serious
				3	4	Urine occult blood positive	Clinical examination	Urine occult blood positive	-	2022-07-28	2022-07-29	2 days	2 days	Recovery	Mild	Non-serious
Diffuse lesion	Kobe-10	Male	13	1	4	Pain	General and systemic disorders and conditions at the site of administration	Pain	-	2022-09-07	2022-09-16	1 day	10 days	Recovery	Mild	Non-serious
				2	1	Hemoglobinuria	Renal and urinary tract disorders	Hemoglobinuria	-	2022-09-07	2022-09-07	1 day	1 day	Recovery	Mild	Non-serious
				3	4	Ddecreased urine output	Clinical examination	Ddecreased urine output	-	2022-09-07	2022-09-08	1 day	2 days	Recovery	Mild	Non-serious
Diffuse lesion	Seiiku-01	Male	5	3	1	Hemoglobinuria	Renal and urinary tract disorders	Hemoglobinuria	-	2022-01-18	2022-01-19	1 day	2 days	Recovery	Mild	Non-serious

Adverse drug reactions
Analysis Subject: SAS
*1 1: Hemoglobinuria, 2: Blistering, 3: Swelling, 4: Other
*2 Number of days from the date of the first administration

MedDRA/J Ver. 26.0

Lesion	Case number	Gender	Age (years)	No.	Classification *1	Condition listed by Physician	System Organ Class	Preferred Term	Investigational drug treatment	Other treatment	Causal relationship	Reasons for no causal relationship	Other details	Comment
Diffuse lesion	Kobe-06	Female	53	1	4	Pain	General and systemic disorders and conditions at the site of administration	Pain	Not applicable	Treatment implementation	Causal relationship exists	−	−	−
				2	4	Urine latent blood positivity	Clinical examination	Urine latent blood positivity	Not applicable	No treatment	Causal relationship exists	−	−	−
Diffuse lesion	Kobe-08	Female	12	1	4	Pain	General and systemic disorders and conditions at the site of administration	Pain	Not applicable	Treatment implementation	Causal relationship exists	−	−	−
				2	2	blister	Skin and subcutaneous tissue disorders	blister	Not applicable	Treatment implementation	Causal relationship exists	−	−	−
				3	4	Urine occult blood positive	Clinical examination	Urine occult blood positive	Not applicable	No treatment	Causal relationship exists	−	−	−
Diffuse lesion	Kobe-10	Male	13	1	4	Pain	General and systemic disorders and conditions at the site of administration	Pain	Not applicable	Treatment implementation	Causal relationship exists	−	−	−
				2	1	Hemoglobinuria	Renal and urinary tract disorders	Hemoglobinuria	Not applicable	Treatment implementation	Causal relationship exists	−	−	−
				3	4	Ddecreased urine output	Clinical examination	Ddecreased urine output	Not applicable	Treatment implementation	Causal relationship exists	−	−	−
Diffuse lesion	Seiiku-01	Male	5	3	1	Hemoglobinuria	Renal and urinary tract disorders	Hemoglobinuria	Not applicable	Treatment implementation	Causal relationship exists	−	−	Haptoglobin was administered.

Adverse drug reactions
Analysis Subject: SAS
*1 1: Hemoglobinuria, 2: Blistering, 3: Swelling, 4: Other
*2 Number of days from the date of the first administration

Lesion	Case number	Gender	Age (years)	No.	Classification *1	Condition listed by Physician	System Organ Class	Preferred Term	Significant adverse events	Date of onset	Date of confirmation/ disappearance date/death date	Number of days*2	Duration	Outcome	Severity	Seriousness
Diffuse lesion	Osaka-01	Male	25	1	4	Pain	General and systemic disorders and conditions at the site of administration	Pain	-	2021-02-02	2021-04-28	1 day	86 days	Recovery	Moderate	Non-serious
				2	1	Hemoglobinuria	Renal and urinary tract disorders	Hemoglobinuria	-	2021-02-02	2021-02-05	1 day	4 days	Recovery	Mild	Non-serious
				3	4	Urinary sugar	Clinical examination	Urinary sugar	-	2021-02-03	2021-02-05	2 days	3 days	Recovery	Mild	Non-serious
				4	4	Subcutaneous hemorrhage	Skin and subcutaneous tissue disorders	Subcutaneous hemorrhage	-	2021-02-03	2021-04-28	2 days	85 days	Recovery	Mild	Non-serious
				5	3	Swelling	General and systemic disorders and conditions at the site of administration	Swelling	-	2021-02-02	2021-03-03	1 day	30 days	Recovery	Mild	Non-serious
Diffuse lesion	Osaka-02	Male	6	2	1	Hemoglobinuria	Renal and urinary tract disorders	Hemoglobinuria	-	2021-07-27	2021-07-30	1 day	4 days	Recovery	Moderate	Non-serious
				3	4	Pain	General and systemic disorders and conditions at the site of administration	Pain	-	2021-07-27	2021-08-25	1 day	30 days	Recovery	Mild	Non-serious
Diffuse lesion	Osaka-04	Male	25	1	4	Postoperative Pain	General and systemic disorders and conditions at the site of administration	Pain	-	2021-11-16	2022-02-09	1 day	86 days	Recovery	Mild	Non-serious

Adverse drug reactions
Analysis Subject: SAS
*1 1: Hemoglobinuria, 2: Blistering, 3: Swelling, 4: Other
*2 Number of days from the date of the first administration

MedDRA/J Ver. 26.0

Lesion	Case number	Gender	Age (years)	No.	Classification *1	Condition listed by Physician	System Organ Class	Preferred Term	Investigational drug treatment	Other treatment	Causal relationship	Reasons for no causal relationship	Other details	Comment
Diffuse lesion	Osaka-01	Male	25	1	4	Pain	General and systemic disorders and conditions at the site of administration	Pain	Not applicable	Treatment implementation	Causal relationship exists	–	–	–
				2	1	Hemoglobinuria	Renal and urinary tract disorders	Hemoglobinuria	Not applicable	Treatment implementation	Causal relationship exists	–	–	–
				3	4	Urinary sugar	Clinical examination	Urinary sugar	Not applicable	No treatment	Causal relationship exists	–	–	–
				4	4	Subcutaneous hemorrhage	Skin and subcutaneous tissue disorders	Subcutaneous hemorrhage	Not applicable	No treatment	Causal relationship exists	–	–	–
				5	3	Swelling	General and systemic disorders and conditions at the site of administration	Swelling	Not applicable	No treatment	Causal relationship exists	–	–	–
Diffuse lesion	Osaka-02	Male	6	2	1	Hemoglobinuria	Renal and urinary tract disorders	Hemoglobinuria	Not applicable	Treatment implementation	Causal relationship exists	–	–	–
				3	4	Pain	General and systemic disorders and conditions at the site of administration	Pain	Not applicable	Treatment implementation	Causal relationship exists	–	–	–
Diffuse lesion	Osaka-04	Male	25	1	4	Postoperative Pain	General and systemic disorders and conditions at the site of administration	Pain	Not applicable	Treatment implementation	Causal relationship exists	–	–	–

Adverse drug reactions
Analysis Subject: SAS
*1 1: Hemoglobinuria, 2: Blistering, 3: Swelling, 4: Other
*2 Number of days from the date of the first administration

Lesion	Case number	Gender	Age (years)	No.	Classification *1	Condition listed by Physician	System Organ Class	Preferred Term	Significant adverse events	Date of onset	Date of confirmation/ disappearance date/death date	Number of days*2	Duration	Outcome	Severity	Seriousness
Diffuse lesion	Osaka-05	Female	17	1	4	Postoperative Pain	General and systemic disorders and conditions at the site of administration	Pain	-	2021-12-21	2022-01-05	1 day	16 days	Recovery	Moderate	Non-serious
				2	4	Subcutaneous hemorrhage	Skin and subcutaneous tissue disorders	Subcutaneous hemorrhage	-	2021-12-23	2022-01-05	3 days	14 days	Recovery	Mild	Non-serious
Diffuse lesion	Tokyo-02	Female	16	2	4	Elevated CK	Clinical examination	Creatine phosphokinase increased	-	2021-07-29	2021-08-02	2 days	5 days	Recovery	Mild	Non-serious
				3	4	Postoperative Pain	General and systemic disorders and conditions at the site of administration	Pain	-	2021-07-28	2021-08-09	1 day	13 days	Recovery	Mild	Non-serious
Diffuse lesion	Tokyo-03	Female	59	1	1	Hemoglobinuria	Renal and urinary tract disorders	Hemoglobinuria	-	2021-12-22	2021-12-23	1 day	2 days	Recovery	Mild	Non-serious
Diffuse lesion	Shinshu-01	Female	14	1	1	Hemoglobinuria	Renal and urinary tract disorders	Hemoglobinuria	-	2021-08-11	2021-08-14	1 day	4 days	Recovery	Mild	Non-serious
				2	4	Postoperative Pain	General and systemic disorders and conditions at the site of administration	Pain	-	2021-08-11	2021-08-14	1 day	4 days	Recovery	Mild	Non-serious
				3	4	Residual subcutaneous hemorrhage	Skin and subcutaneous tissue disorders	Subcutaneous hemorrhage	-	2021-08-24	2021-11-09	14 days	78 days	Light	Mild	Non-serious

Adverse drug reactions
Analysis Subject: SAS
*1 1: Hemoglobinuria, 2: Blistering, 3: Swelling, 4: Other
*2 Number of days from the date of the first administration

MedDRA/J Ver. 26.0

Lesion	Case number	Gender	Age (years)	No.	Classification *1	Condition listed by Physician	System Organ Class	Preferred Term	Investigational drug treatment	Other treatment	Causal relationship	Reasons for no causal relationship	Other details	Comment
Diffuse lesion	Osaka-05	Female	17	1	4	Postoperative Pain	General and systemic disorders and conditions at the site of administration	Pain	Not applicable	Treatment implementation	Causal relationship exists	–	–	–
				2	4	Subcutaneous hemorrhage	Skin and subcutaneous tissue disorders	Subcutaneous hemorrhage	Not applicable	No treatment	Causal relationship exists	–	–	–
Diffuse lesion	Tokyo-02	Female	16	2	4	Elevated CK	Clinical examination	Creatine phosphokinase increased	Not applicable	No treatment	Causal relationship exists	–	–	The event has been confirmed to have occurred with the administration of oldamine, and a causal relationship was also considered from the time line.
				3	4	Postoperative Pain	General and systemic disorders and conditions at the site of administration	Pain	Not applicable	Treatment implementation	Causal relationship exists	–	–	–
Diffuse lesion	Tokyo-03	Female	59	1	1	Hemoglobinuria	Renal and urinary tract disorders	Hemoglobinuria	Not applicable	Treatment implementation	Causal relationship exists	–	–	–
Diffuse lesion	Shinshu-01	Female	14	1	1	Hemoglobinuria	Renal and urinary tract disorders	Hemoglobinuria	Not applicable	Treatment implementation	Causal relationship exists	–	–	–
				2	4	Postoperative Pain	General and systemic disorders and conditions at the site of administration	Pain	Not applicable	Treatment implementation	Causal relationship exists	–	–	–
				3	4	Residual subcutaneous hemorrhage	Skin and subcutaneous tissue disorders	Subcutaneous hemorrhage	Not applicable	No treatment	Causal relationship exists	–	–	Decided that no follow-up was necessary due to follow-up with usual medical care at the last visit (2021.11.9).

Adverse drug reactions
Analysis Subject: SAS
*1 1: Hemoglobinuria, 2: Blistering, 3: Swelling, 4: Other
*2 Number of days from the date of the first administration

Lesion	Case number	Gender	Age (years)	No.	Classification *1	Condition listed by Physician	System Organ Class	Preferred Term	Significant adverse events	Date of onset	Date of confirmation/ disappearance date/death date	Number of days*2	Duration	Outcome	Severity	Seriousness
Diffuse lesion	Shinshu-03	Female	52	1	4	Postoperative Pain	General and systemic disorders and conditions at the site of administration	Pain	-	2022-02-18	2022-03-08	3 days	19 days	Recovery	Mild	Non-serious
				2	1	Hemoglobinuria	Renal and urinary tract disorders	Hemoglobinuria	-	2022-02-16	2022-02-18	1 day	3 days	Recovery	Mild	Non-serious
Diffuse lesion	Shinshu-04	Male	10	2	1	Hemoglobinuria	Renal and urinary tract disorders	Hemoglobinuria	-	2022-08-24	2022-08-26	1 day	3 days	Recovery	Mild	Non-serious
				3	4	Postoperative Pain	General and systemic disorders and conditions at the site of administration	Pain	-	2022-08-24	2022-11-15	1 day	84th day	Recovery	Mild	Non-serious
				4	3	Swelling	General and systemic disorders and conditions at the site of administration	Swelling	-	2022-08-25	2022-11-15	2 days	83 days	Recovery	Mild	Non-serious
Diffuse lesion	Shinshu-05	Female	10	1	1	Hemoglobinuria	Renal and urinary tract disorders	Hemoglobinuria	-	2022-12-07	2022-12-08	1 day	2 days	Recovery	Mild	Non-serious
				2	4	Postoperative Pain	General and systemic disorders and conditions at the site of administration	Pain	-	2022-12-07	2023-01-17	1 day	42 days	Recovery	Mild	Non-serious
				3	4	Subcutaneous hemorrhage	Skin and subcutaneous tissue disorders	Subcutaneous hemorrhage	-	2022-12-07	2022-12-09	1 day	3 days	Light	Mild	Non-serious
				4	3	Swelling	General and systemic disorders and conditions at the site of administration	Swelling	-	2022-12-07	2023-01-17	1 day	42 days	Recovery	Mild	Non-serious

Adverse drug reactions
Analysis Subject: SAS
*1 1: Hemoglobinuria, 2: Blistering, 3: Swelling, 4: Other
*2 Number of days from the date of the first administration

MedDRA/J Ver. 26.0

Lesion	Case number	Gender	Age (years)	No.	Classification *1	Condition listed by Physician	System Organ Class	Preferred Term	Investigational drug treatment	Other treatment	Causal relationship	Reasons for no causal relationship	Other details	Comment
Diffuse lesion	Shinshu-03	Female	52	1	4	Postoperative Pain	General and systemic disorders and conditions at the site of administration	Pain	Not applicable	Treatment implementation	Causal relationship exists	−	−	−
				2	1	Hemoglobinuria	Renal and urinary tract disorders	Hemoglobinuria	Not applicable	Treatment implementation	Causal relationship exists	−	−	−
Diffuse lesion	Shinshu-04	Male	10	2	1	Hemoglobinuria	Renal and urinary tract disorders	Hemoglobinuria	Not applicable	Treatment implementation	Causal relationship exists	−	−	−
				3	4	Postoperative Pain	General and systemic disorders and conditions at the site of administration	Pain	Not applicable	Treatment implementation	Causal relationship exists	−	−	−
				4	3	Swelling	General and systemic disorders and conditions at the site of administration	Swelling	Not applicable	No treatment	Causal relationship exists	−	−	−
Diffuse lesion	Shinshu-05	Female	10	1	1	Hemoglobinuria	Renal and urinary tract disorders	Hemoglobinuria	Not applicable	Treatment implementation	Causal relationship exists	−	−	−
				2	4	Postoperative Pain	General and systemic disorders and conditions at the site of administration	Pain	Not applicable	Treatment implementation	Causal relationship exists	−	−	−
				3	4	Subcutaneous hemorrhage	Skin and subcutaneous tissue disorders	Subcutaneous hemorrhage	Not applicable	No treatment	Causal relationship exists	−	−	No treatment is required, and the trial is terminated because the patient is followed up in regular medical care.
				4	3	Swelling	General and systemic disorders and conditions at the site of administration	Swelling	Not applicable	No treatment	Causal relationship exists	−	−	−
