# Supplementary material for: Effect and safety of ethanolamine oleate in sclerotherapy in patients with difficult-to-resect venous malformations: A multicenter, single-arm study
Source: PLoS One. 2025 Jan 31;20(1):e0303130. doi: 10.1371/journal.pone.0303130 (PMC11785324; doi:10.1371/journal.pone.0303130)
Supplement: Attachment — Submitted filename: (0906)Response_to_Reviewers.docx [file pone.0303130.s010.docx]

**To academic editor**

Thank you for providing the necessary information for this paper. I have uploaded the information as indicated below for your review.

1. The protocol has been uploaded to ‘protocols.io’.
2. In response to one of the journal requirements to make the foundational data underlying the manuscript available, we have uploaded five files as supplementary information. These files include baseline data on lesion volume reduction and alterations, pain changes, adverse events, and adverse effects. Additionally, the description names have been listed under the 'Supporting Information' section.

**To Reviewer #1**

Thank you for reviewing our manuscript. We have carefully considered your valuable feedback and have provided point-by-point responses for your kind review.

• Have the authors made all data underlying the findings in their manuscript fully available?

(response) We have provided the foundational data underlying this manuscript as Supporting information.

• Please go through the manuscript and correct for the use of the word efficacy. Usually "efficacy" is associated estimation of the treatment effect in a comparative trial. Here the word "effect" - meaning estimation of the effect of a treatment - should be used.

(response) We have changed all instances of the term “efficacy” to “effect” in the manuscript.

• The term "open label" is rather obvious in a single arm trial and can be deleted.

(response) We have removed the term “open label” from all instances where 'single arm trial' is mentioned, including in the title.

• There are two groups considered resulting in the same sample size. This seem to be surprising! Please make clear whether a stratified sampling in the two groups (cystic type or diffuse) is applied. Figure 1 has to be modified in this case.

(response) We have treated the two groups as separate cohorts; however, the primary and secondary endpoints are common to both. We apologize for any confusion this may have caused. The sample size is the same for both groups based on the recommendation from the Pharmaceuticals and Medical Devices Agency (PMDA) of Japan. To clarify, we have separated the groups in Figure 1.

• I assume the logic of the design is to estimated the overall (pooled) effect across both groups. Please explain the logic of the study design.

(response) It is clinically evident that the treatment effects of sclerotherapy differ between cystic and diffuse lesions. Therefore, this study prioritized evaluating the reduction effect on cystic lesions. It was known during the planning phase of the study that the reduction effect for diffuse lesions would be inferior to that for cystic lesions. However, the Pharmaceuticals and Medical Devices Agency (PMDA) required that if this drug was to be used for diffuse lesions as well, it should be evaluated in this study. Consequently, we established two separate cohorts for this purpose. Given the nature of this content, we may be difficult to include it in the main text.

• Please clarify whether this is a pediatric trial or a trial in an adult and pediatric population.

(response) This study involves research on both adults and pediatric populations. We have also added this information to the text.

• The term "changes" is unclear at this place of the paper.

(response)The term “difference” was used for changes in volume and pain.

• please delete open-label and include single arm
(response) We have made the appropriate revisions, including adjustments to the title.

• The information of the sample size justification is somewhat confusing. Please provide some text.

(response) We have revised the information regarding the sample size accordingly.

• Definition of the primary endpoint is not aligned with the text in L70/71/72 where two endpoints are mentioned.

(response) We have added and revised the relevant text to address the inconsistencies regarding the primary endpoints.

• Analysis set description does not need extensive text. This paragraph should be summarized in one sentence.

(response) As stated in line 311, FAS, PPS, and SAS represent the same population, although we had defined FAS and PPS separately in the study design, as stated in line 282.

Despite the slight redundancy, we prefer to retain the current description in the Statistical analysis of endpoints section, where the study design is described.

• I do not like layman expressions like "relatively limited sample size". The term "relatively" is confusing.

(response) We have removed the term “relatively.”

• Please argue why a comparative study of ethanolamine against placebo is not possible or considered here.

(response) The reasons for not including a placebo are as follows: Sclerotherapy for venous malformations is well-established and recognized as effective in several guidelines and textbooks and the medical community. However, there has been a lack of high level evidence in clinical research reports, which is why this study was conducted. Additionally, when initiating this study, we consulted with the Pharmaceuticals and Medical Devices Agency (PMDA) in Japan regarding the protocol and were advised that a placebo was not necessary. Furthermore, because this treatment requires hospitalization and general anesthesia and the condition is progressive, it was deemed ethically challenging to include a placebo group. This information has been added to the main text.

• Please elaborate the influence of bias (allocation bias, detection bias, performance bias) on the study results.

(response) This is not a randomized controlled trial. The term “allocation bias” is reminiscent of a randomized controlled trial; thus, we have used “selection bias” instead.

We have examined the impact of potential biases. Regarding selection bias, although some cases with predominantly diffuse lesions may have been included in the cystic group if their target lesions were cystic, pain assessments in the cystic group could still be influenced by diffuse lesions. However, we consider the likelihood of selection bias to be low.

Regarding detection bias, the evaluation of lesion volumes was conducted blindly by two external assessors, which minimizes potential bias. Concerning performance bias, although the same technique was used for injecting the sclerosant in both cystic and diffuse lesions, there might be variability in the amount of sclerosant injected in diffuse lesions owing to uncertain areas where the sclerosant may leak out. However, because this study is not a comparative trial, the impact of this bias on the study results is not considered a major issue. This information has been added to the main text.

**To Reviewer #2**

Thank you for reviewing our manuscript. We have carefully considered your valuable feedback and have provided point-by-point responses here for your review. This study is a non-comparative trial and involves two separate cohorts, which we have specifically explained.

• How were these lesions determined to be "difficult to resect"? Was this based on an interdisciplinary team decision or a single provider's gestalt?

(response) Thank you for your suggestion. The determination of “difficult to resect” is made by the “investigator” or “sub-investigator.” The criteria for this judgment are described in the “Participant Inclusion Criteria” section, specifically on lines L198–200. We apologize for any confusion.

• Is Ethanolamine oleate FDA approved for this indication? If not, this will be difficult to gain any traction clinically in the United States.

(response) Ethanolamine oleate is not currently approved by the FDA for treating venous malformations. The lack of approval is owing to the prevalence of low level evidence of clinical studies. This clinical trial was conducted with the aim of generating high level evidence in research results, and we plan to use the results to obtain regulatory approval in Japan. We are currently in the process of applying for regulatory approval, which will hopefully be granted.

• The patient demographics should have statistical analyses performed to demonstrate that there were no significant differences between the cystic and diffuse cohort.

(response) We considered cystic and diffuse types as different disease states and believed that the patient background and efficacy of treatment may differ depending on the disease state. Thus, we designed a study to evaluate the response rate for each disease type as a separate cohort with different populations and did not plan to compare the response rate between disease types. The study was planned as a separate cohort with different populations to evaluate the response rate by lesion type. If the objective had been to compare response rates between lesion types, the *p*-value or standardized mean difference (SMD) might be included as an indicator for comparison of patient backgrounds to explain comparability. However, because this is not the case in our study, such indicators were not included. Particularly, including the *p*-value of the test in the patient background table has traditionally been performed in randomized controlled trials, implying that the randomization confirms that the distribution of patient backgrounds is aligned across the groups to be compared. In this study, we are concerned that the inclusion of *p*-values for the test of comparison of cystic and diffuse type patient backgrounds may mislead the reader into believing that this is a randomized controlled trial. In addition, as stated in the ASA statement [1], the abuse of *p*-values has recently been discouraged, and SMDs are being used instead of *p*-values for the comparison of background items. However, to reiterate, this study is not intended to compare lesion types; therefore, we would not describe the measure of comparison between lesion types.

A patient background table with *p*-values and SMDs is presented in this letter as a reference.

1) Wasserstein RL, Lazar NA. Editorial: The ASA’s statement on p-values: Context, process, and purpose. The American Statistician 2016; 70: 129-133.

|  |  | **All**  **n = 44** | **Cystic Type**  **n = 22** | **Diffuse Type**  **n = 22** | ***p*-value*** | **SMD**  **(Ref. Diffuse Type)** |
| --- | --- | --- | --- | --- | --- | --- |
| **Age (year)** | Mean | 22.9 | 22.5 | 23.3 | 0.887 | -0.044 |
|  | SD | 17.6 | 18.8 | 16.9 |  |  |
|  | Min | 3 | 3 | 5 |  |  |
|  | Median | 16.5 | 16.5 | 16.5 |  |  |
|  | Max | 78 | 78 | 59 |  |  |
| **Sex (%)** | Male | 22 (50.0) | 13 (59.1) | 9 (40.9) | 0.366 | 0.370 |
|  | Female | 22 (50.0) | 9 (40.9) | 13 (59.1) |  |  |
| **Body weight (kg)** | Mean | 47.37 | 45.73 | 49.02 | 0.596 | -0.164 |
|  | SD | 20.26 | 24.32 | 15.6 |  |  |
|  | Min | 14.7 | 14.7 | 20.3 |  |  |
|  | Median | 49.1 | 49.1 | 50.5 |  |  |
|  | Max | 92 | 92 | 75.2 |  |  |
| **Location of venous malformations (%)** | Trunk | 9 (20.5) | 5 (22.7) | 4 (18.2) | 0.014 | 1.033 |
|  | Upper limb | 11 (25.0) | 3 (13.6) | 8 (36.4) |  |  |
|  | Lower limb | 15 (34.1) | 5 (22.7) | 10 (45.5) |  |  |
|  | Face | 5 (11.4) | 5 (22.7) | 0 (0.0) |  |  |
|  | Oral | 1 (2.3) | 1 (4.5) | 0 (0.0) |  |  |
|  | Neck | 3 (6.8) | 3 (13.6) | 0 (0.0) |  |  |
| **Previous treatment for venous malformations (%)** | Yes | 22 (50.0) | 10 (45.5) | 12 (54.5) | 0.764 | 0.182 |
|  | No | 22 (50.0) | 12 (54.5) | 10 (45.5) |  |  |
| **Treatment history of sclerotherapy for target lesion (%)** | Initial treatment | 30 (68.2) | 17 (77.3) | 13 (59.1) | 0.332 | 0.364 |
|  | Retreatment | 14 (31.8) | 5 (22.7) | 9 (40.9) |  |  |

• Did any patients require resection following the ethanolamine injection? How many required repeated injections to maintain these results?

(response) Because the study primarily targets patients with lesions that were difficult to resect, none of the patients in the follow-up range underwent resection. Additionally, the study was designed to allow for additional treatment if pain was not sufficiently alleviated after a single sclerotherapy session; however, no patients required additional treatment. Information regarding additional treatment has been added to the “secondary endpoint” section.

• A 50% adverse effect rate is quite high for any sort of medication/therapy, how do the authors propose this could be avoided or is there another way to preemptively avoid these adverse effects?

(response) The study observed a high rate of adverse effects; however, most of these were due to temporary inflammation resulting from the pharmacological effects of sclerotherapy. Overall, the safety of the treatment is considered to be within acceptable limits. The side effect that requires attention is hemoglobinuria; however, renal damage due to hemolysis has been completely prevented with the administration of haptoglobin. Therefore, the safety of the treatment is deemed to be adequately ensured. We have revised the text to emphasize these points.

• The authors need to include controls - patients with difficult to resect AVMs that only receive saline injection and demonstrate that a similar effect is not seen with saline injections alone. Perhaps the tumescence from the volume of the injection is enough to cause the AVM to vasospasm and shrink.

(response) The reasons for not including a placebo are as follows: Sclerotherapy for venous malformations is well-established and recognized as effective in several guidelines and textbooks and the medical community. However, there has been a lack of high level evidence in clinical research reports, which is why this study was conducted. Additionally, when initiating this study, we consulted with the Pharmaceuticals and Medical Devices Agency (PMDA) in Japan regarding the protocol and were advised that a placebo was not necessary. Furthermore, because this treatment requires hospitalization and general anesthesia and the condition is progressive, it was deemed ethically challenging to include a placebo group. This information has been added to the main text. Additionally, the pharmacological effect of ethanolamine oleate as a sclerosing agent has been demonstrated in the treatment of esophageal varices and gastric varices, where it acts on vascular endothelial cells to induce lesion shrinkage. Regulatory approvals for these conditions are well-established. Therefore, it is evident that a similar pharmacological effect would impair lesions in venous malformations.
